# Genetic variation and pesticide exposure influence blood DNA methylation signatures in females with early-stage Parkinson’s disease

**DOI:** 10.1038/s41531-024-00704-3

**Published:** 2024-05-07

**Authors:** S. L. Schaffner, W. Casazza, F. Artaud, C. Konwar, S. M. Merrill, C. Domenighetti, J. M. Schulze-Hentrich, S. Lesage, A. Brice, J. C. Corvol, S. Mostafavi, J. K. Dennis, A. Elbaz, M. S. Kobor, DIGPD Study Group

**Affiliations:** 1 Edwin S. H. Leong Centre for Healthy Aging, Faculty of Medicine, University of British Columbia, Vancouver, BC Canada; 2 Centre for Molecular Medicine and Therapeutics, BC Children’s Hospital, Vancouver, BC Canada; 3 Department of Medical Genetics, University of British Columbia, Vancouver, BC Canada; 4 Bioinformatics Graduate Program, University of British Columbia, Vancouver, BC Canada; 5 Université Paris-Saclay, UVSQ, Inserm, Gustave Roussy, CESP, 94805 Villejuif, France; 6 Department of Genetics/Epigenetics, Faculty NT, Saarland University, 66041 Saarbrücken, Germany; 7 Sorbonne Université, Institut du Cerveau-Paris Brain Institute-ICM, INSERM, CNRS, Assistance Publique Hôpitaux de Paris, Paris, France; 8 Sorbonne University, Assistance Publique Hôpitaux de Paris, Paris Brain Insitute – ICM, Inserm, CNRS, Department of Neurology and CIC Neurosciences, Pitié-Salpêtrière Hospital, Paris, France; 9 Paul Allen School of Computer Science and Engineering, University of Washington, Seattle, WA USA

**Keywords:** Epigenetics, Parkinson's disease

## Abstract

Although sex, genetics, and exposures can individually influence risk for sporadic Parkinson’s disease (PD), the joint contributions of these factors to the epigenetic etiology of PD have not been comprehensively assessed. Here, we profiled sex-stratified genome-wide blood DNAm patterns, SNP genotype, and pesticide exposure in agricultural workers (71 early-stage PD cases, 147 controls) and explored replication in three independent samples of varying demographics (*n* = 218, 222, and 872). Using a region-based approach, we found more associations of blood DNAm with PD in females (69 regions) than in males (2 regions, Δβ_adj_| ≥0.03, *p*_adj_ ≤ 0.05). For 48 regions in females, models including genotype or genotype and pesticide exposure substantially improved in explaining interindividual variation in DNAm (*p*_adj_ ≤ 0.05), and accounting for these variables decreased the estimated effect of PD on DNAm. The results suggested that genotype, and to a lesser degree, genotype-exposure interactions contributed to variation in PD-associated DNAm. Our findings should be further explored in larger study populations and in experimental systems, preferably with precise measures of exposure.

## Introduction

Parkinson’s disease (PD) is a neurodegenerative disorder, the prevalence of which is increasing worldwide, affecting an estimated 9.4 million individuals as of 2020^1,2^. PD is associated with aggregations in the brain known as Lewy bodies, composed primarily of α-synuclein protein, which are confirmed upon post-mortem examination and begin accumulating years to decades prior to the onset of motor symptoms and clinical diagnosis^3^. Beyond the brain, inflammatory changes are also associated with PD, as reduced lymphocyte levels can be detected prior to motor symptom onset^4^. As clinical PD progresses over an approximately 14-year period from diagnosis to the most advanced disease stage (Hoehn and Yahr stage V), studying living individuals at the earliest stage of the disease may provide insight into its molecular etiology at a time when sufficient symptoms have appeared for diagnosis and similar pathology to the pre-symptomatic stage is assumed to be present^5^. While approximately 10% of PD cases show monogenic inheritance, the remaining 90% are considered “sporadic,” with common genetic variations and environment/lifestyle factors influencing the etiology^6^. The complex etiology, late-life, gradual onset of symptoms, and the presence of inflammatory changes and Lewy bodies prior to clinical diagnosis of sporadic PD highlights the need for a heightened understanding of early-stage disease pathogenesis, using peripheral tissue samples such as blood, saliva, and cerebrospinal fluid (CSF) that can be obtained in a minimally invasive manner in living patients^7^. Improving our understanding of molecular changes associated with early PD could also present new opportunities for early interventions, that ideally might slow the course of disease progression^8^.

DNA methylation (DNAm), the most commonly studied epigenetic mark in human populations, may embed genetic and environmental contributions to PD risk, and when measured in blood, it can also reflect immune-based changes observed in early PD pathogenesis^4,9^. Blood DNAm changes have been detected in prodromal PD and in patients with a disease duration of 4 years or less (Hoehn and Yahr stage ≤3), suggesting that DNAm could be used to assess molecular changes in patients with early- to mid-stage PD^10,11^. However, although several studies reported PD-associated changes in blood DNAm at site-specific and regional levels, the number of associations replicated between studies decreased as sample size increased, with the largest blood-based PD epigenome-wide association study (EWAS) to date reporting that only two cytosine-guanine (CpG) sites remained after a meta-analysis consisting of over 2000 individuals of European descent (1132 cases, 999 controls)^10,12–15^. This resembles the “Winner’s Curse” effect commonly discussed in genetic analysis, where genome-wide association (GWAS) studies show low replication across independent studies^16^. The “Winner’s Curse” in PD EWAS may be explained by considerable heterogeneity in study design, population structure, disease presentation, pathology, genome, individual lifestyle factors, and exposure history across individuals diagnosed with PD. As such, a balance between achieving sufficient power for detection of possible PD-associated DNAm differences while addressing the wide range of variation in PD and its risk factors is desirable for the next generation of PD EWAS studies.

In addition, most PD EWAS to date have been performed in patients with advanced PD, and the majority used single-CpG approaches, which do not easily capture the spatially correlated nature of DNAm. A few studies applied region-based approaches including comb-p, DMRcate, and BumpHunter, which can address this spatial correlation and reduce the multiple test correction burden in high-dimensional EWAS; however, replication of results between these studies was limited^10,17,18^. Other region-based approaches such as the recently developed CoMeBack have built upon DMRcate by identifying correlated DNAm patterns within individuals in an unsupervised manner, which has the potential to improve reproducibility between studies^19^. Taken together, the extent to which regional changes in DNAm early in the course of PD progression can be consistently detected across different populations remains to be elucidated.

At a conceptual level, DNAm patterns are associated with many of the same genetic and environmental factors which underpin PD risk, presenting additional challenges in epigenetic studies of PD. For instance, single nucleotide polymorphisms (SNPs) contribute to approximately 20% of variation in population blood DNAm levels^20,21^. Sporadic PD also has a genetic component, as evidenced by twin studies indicating an overall heritability rate of 27%, with 90 common genetic variants accounting for roughly 22% of this risk^22–29^. In attempts to explain the substantial remainder of PD risk not attributed to genetic variation, a number of environmental and lifestyle-related factors have also been shown to be associated with either increased (e.g., pesticides, dairy intake, solvents, head trauma) or decreased (e.g., smoking, caffeine intake, physical activity, ibuprofen use) risk of PD at a population level^8,30^. Among these, pesticide exposure is one of the most well studied environmental risk factors for the development of PD, with support from both epidemiological and experimental research. Case-control and cohort studies in different countries have shown replicable associations between occupational or environmental exposure to pesticides and PD incidence^31,32^. Early- and mid-life exposure to environmental toxicants has been hypothesized to initiate the abnormal accumulation of α-synuclein in some individuals, possibly to a different extent in those with genetic risk factors for sporadic PD^8,29^. Occupational and ambient pesticide exposure also influence blood DNAm levels in PD patients and in adults without PD, possibly in a sex-specific manner^33–35^. However, the mechanisms by which these environmental insults interact with the genome are not well understood.

The joint impacts of SNP genotype and exposure on DNAm patterns represent one potential mechanism for the contribution of such gene-exposure interactions to PD risk, highlighting the importance of obtaining genotyping and DNAm data from the same individuals for surveying gene (G) × exposure (E) effects in PD epigenomics^36,37^. While a small number of studies have separately assessed impacts of genotype or pesticide exposure on DNAm patterns in PD patients, further research is needed to replicate these associations and assess G × E interactions. To the best of our knowledge, only two studies examined DNAm patterns in blood from PD patients with regard to genotype, neither of which used genetic data from the same individuals, and instead used publicly available GWAS data to investigate associations between DNAm and genotype in PD^12,38^. With respect to pesticides, whether exposure impacts the association between PD and altered DNAm levels is not yet fully understood^39^. However, exposure to organophosphate (a widely used insecticide) affected DNAm patterns differently in PD patients and healthy controls, suggesting possible interactions between exposure, DNAm, and disease^34^. A recent small EWAS in 20 individuals with PD also reported blood DNAm differences between patients who were or were not exposed to organochlorine insecticides, though, studies with larger sample sizes are required to validate these findings^40^.

Finally, biological sex also affects PD incidence, onset, and presentation, as well as influencing variation in DNAm^41–44^. For example, the incidence of PD is 1.5–2-fold higher in males than in females, and the origin of this difference largely remains to be elucidated^42,43,45^. Males with PD are also more likely to experience cognitive decline and faster progression of difficulties with activities of daily living, while females are more likely to experience dyskinesia^42^. Understanding the impact of PD in each sex is important, particularly as females are more likely to experience side effects of current PD treatments^43^. In addition, the prevalence of several of the exposures associated with PD risk (e.g., smoking, pesticides) is considerably different in men and women^41^. Lastly, sex differences in routes of exposure (e.g., household vs. occupational pesticide use) and mechanisms of exposure (e.g., bio-accumulation in adipose tissue) are often present and may or may not be reflected in exposure measurement^46–48^. However, most genetic and epigenetic studies of PD to date have opted to control for sex as a covariate rather than examining sex-specific aspects of PD etiology, with the exception of a recent sex-stratified EWAS in post-mortem brain^10^^,12,14,15^^,24,25,29,49^.

Overall, the gaps in existing research highlight the opportunity for determining the degree to which findings from DNAm studies in PD may be influenced by differences in sex, genetic background, and/or exposure history, and further research in patients in the early stages of the disease is required to better understand the underlying biology at a time when some interventions might be most impactful. In this study, we leveraged a unique sample of French agricultural workers with early-stage sporadic PD (TERRE) for whom detailed pesticide exposure history, whole-blood DNAm, and genotype data were available to identify possible region-based blood DNAm changes associated with early-stage PD. Individuals from TERRE were stratified by sex due to sex differences in PD, autosomal DNAm, and rates of pesticide exposure^41–45,50^. DNAm signatures of PD were subsequently replicated in three independent samples, and tested for their sensitivity to individual genotype and exposure to pesticides.

## Results

### Predicted immune cell type composition was largely unaltered between PD cases and controls in TERRE

**Fig. 1:**
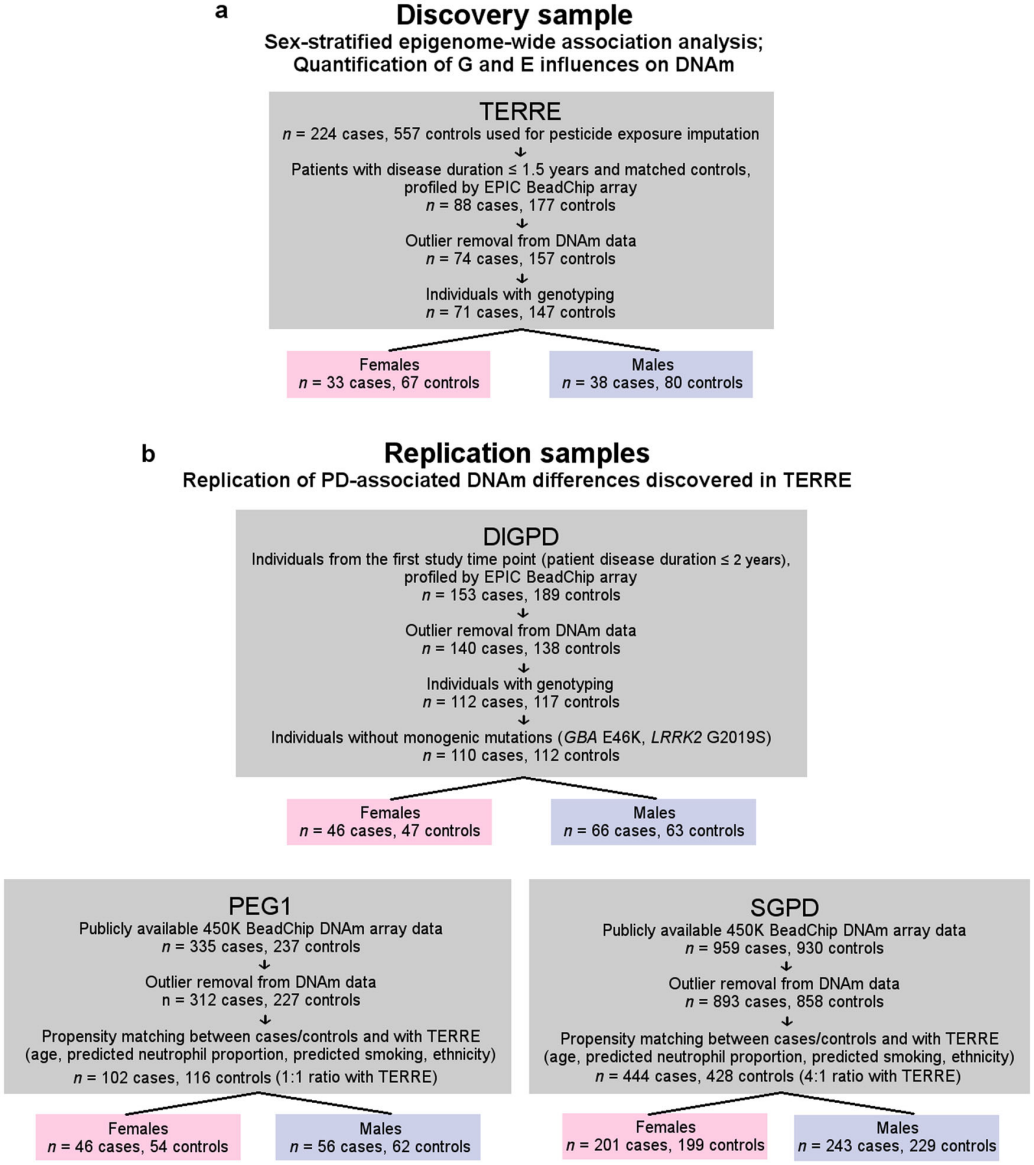
Overview of discovery and replication samples assessed in this study. **a** Discovery sample (TERRE). **b** Replication samples (PEG1, DIGPD, and SGPD). The final numbers of individuals retained after propensity matching on the indicated variables 1) between cases and controls, within each sample, followed by 2) with TERRE are shown.

As immune cell type differences have been reported in PD, we first used DNAm profiles to bioinformatically derive blood cell type proportions in TERRE and test for possible case-control shifts^4,10,12^. For comparison, we also predicted cell type composition from DNAm profiles in three independent samples with differing demographics (Parkinson’s Environment and Genes study, wave 1 (PEG1), including individuals with disease duration ≤3 years recruited from agricultural regions in California, *n* = 539; Drug Interaction With Genes in Parkinson’s Disease (DIGPD), including individuals with disease duration ≤2 years recruited from clinics in France, *n* = 222; and System Genomics of Parkinson’s Disease (SGPD), including individuals of varying disease duration recruited from clinics in Australia and New Zealand, *n* = 1751), two of which (PEG1 and SGPD) were previously published by others^12,15^ (Fig. 1).

**Fig. 2:**
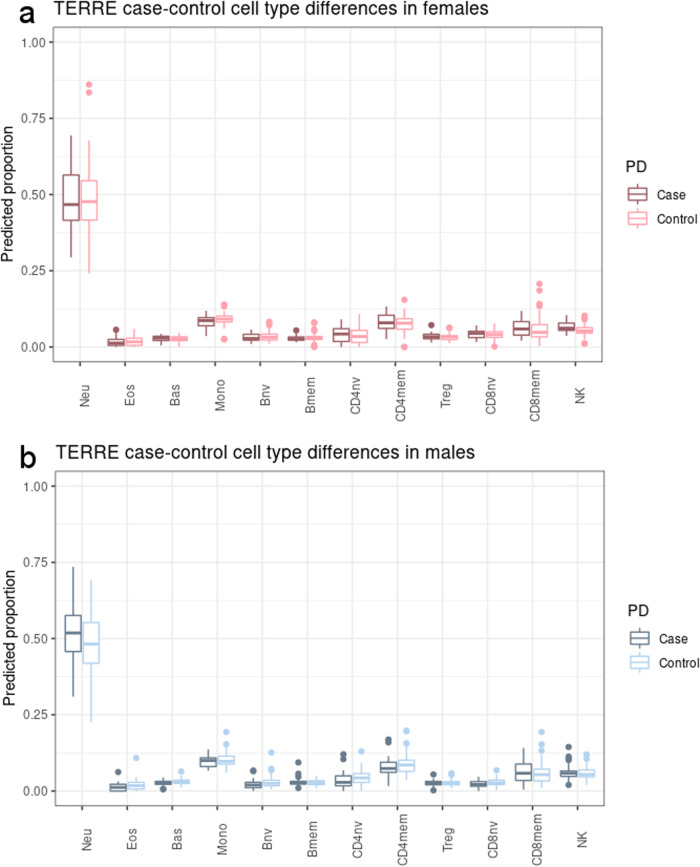
Predicted immune cell composition in PD cases and controls from TERRE, stratified by sex. **a** Predicted cell type proportions in females from TERRE (*n* = 100). Dark pink: PD cases; bright pink: controls. **b** Predicted cell type proportions in males from TERRE (*n* = 118). Dark blue: PD cases; light blue: controls. *p*_adj_ > 0.05 for all case-control comparisons (*t* test with Benjamini–Hochberg adjustment). Centre line: median; box limits: 25th and 75th percentiles; whiskers: 1.5 × interquartile range.

Female PD cases from TERRE showed nonsignificant trends (*p*_adj_ > 0.05) toward lower neutrophil proportion and higher natural killer (NK) cell proportion, in contrast to the decreased NK cells and elevated neutrophils reported previously with PD status and recapitulated in our analysis of DIGPD, PEG1, and SGPD (Fig. 2a, Supplementary Fig. 1A)^4,10,12^. Conversely, males with PD had nonsignificant trends (*p*_adj_ > 0.05) toward decreased CD8^+^ and CD4^+^ naive T cell proportions and decreases in other lymphocytes and increases in neutrophils, more consistent with previously reported immune cell type changes in PD and with the cell type composition profiles we calculated in the three independent samples (Fig. 2b, Supplementary Fig. 1B)^4,10,12^. To reduce the influence of these cell type proportions on DNAm in TERRE, robust principal components (PCs) of cell type composition were included as covariates in all downstream analyses.

### Sex-specific differential DNA methylation was associated with early-stage PD in TERRE in region-based epigenome-wide association analysis

**Fig. 3:**
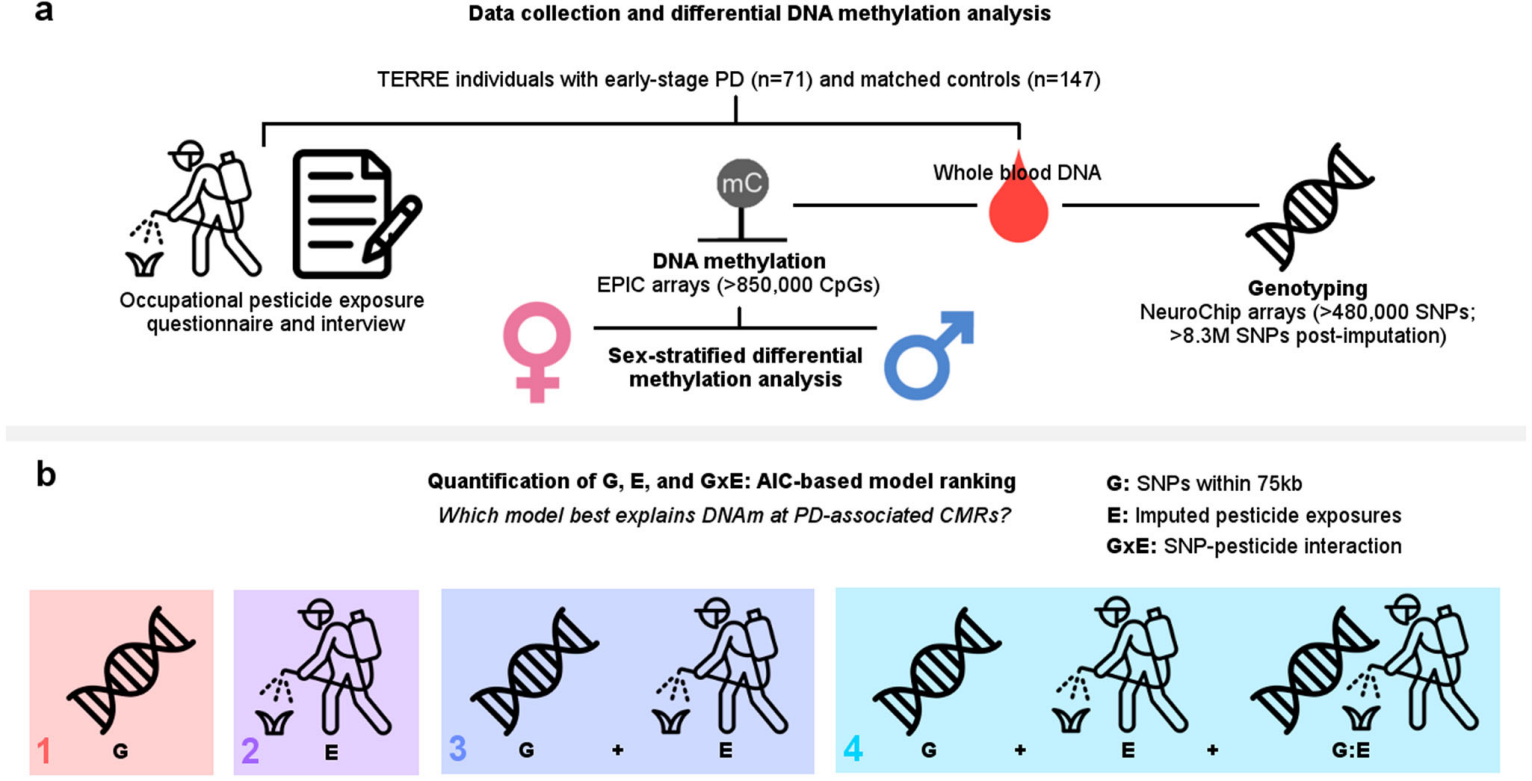
Overview of pesticide exposure, DNA methylation, and genotype data generation and analysis in TERRE. **a** Pipeline for collection of pesticide exposure history, identification of differentially methylated regions, and SNP genotyping. **b** Overview of quantification of G, E, and G × E effects using AIC to rank variance explained by G, E, G + E, and G × E models at each co-methylated region (CMR). Pesticide icon created by Iconjam—Flaticon. Notepad icon created by Freepik—Flaticon. DNA helix icon created by ranksol graphics—Flaticon.

Adopting a reference co-methylated region (CMR)-based method, which can detect regional DNAm patterns correlated within and across individuals, we performed differential DNAm analysis between PD cases and controls on whole-blood reference CMRs with stratification by sex (*n* = 100 females and 118 males) (Fig. 3a). 83% of the 42,776 reference CMRs covered in TERRE (35,561 CMRs) had one or more probes that overlapped with custom CMRs called directly in TERRE using a minimum Spearman correlation of 0.3 and maximum distance cutoff of 1 kb, indicating good correspondence between the reference and our dataset^19^.

**Fig. 4:**
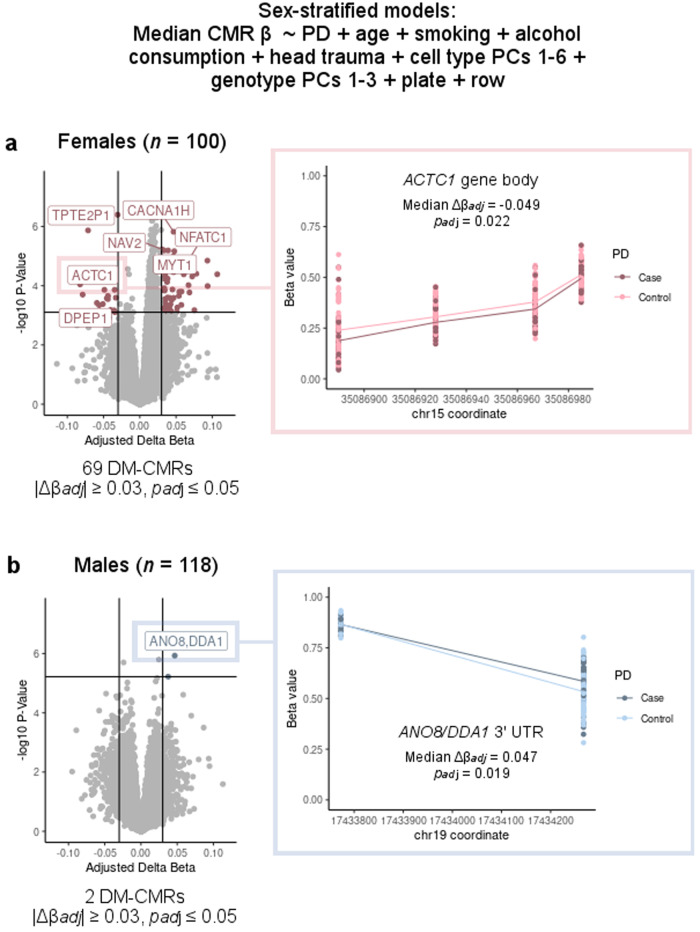
PD-associated differentially methylated CMRs identified with sex stratification. **a** Volcano plot: adjusted PD case-control DNAm differences in females from TERRE (*n* = 100). Colored points pass thresholds of median CMR |Δ*β*_adj_| ≥ 0.03 and *p*_adj_ ≤ 0.05. Inset: the *ACTC1* CMR is shown as a representative example. *y* axis: *β* value (level of DNAm) in female subjects from TERRE. Dark pink: PD cases; bright pink: controls. **b** Volcano plot: Adjusted PD case-control DNAm differences in males from TERRE (*n* = 118). Colored points pass thresholds of median CMR |Δ*β*_adj_| ≥ 0.03 and *p*_adj_ ≤ 0.05. Inset: the *ANO8/DDA1* 3′UTR CMR is shown as a representative example. *y* axis: β value (level of DNAm) in female subjects from TERRE. Dark blue: PD cases; light blue: controls.

With an adjusted *p* value cutoff of 0.05 and an absolute median CMR Δβ_adj_ (PD case-control DNAm difference) cutoff of 0.03, 69 CMRs were differentially methylated in the females-only analysis (median |Δβ_adj_|0.044, IQR 0.035–0.056) (Fig. 4a, Table 1, Supplementary Table 1), and two CMRs were differentially methylated in the males-only analysis (median Δβ_adj_ 0.047 and 0.038) (Fig. 4b, Table 2), all of which had variable DNAm (range >0.05 between the 10th and 90th percentiles of median β values for each CMR) in both sexes except for two CMRs from the females-only analysis (*VSIG1* and chrX:145509155–145509522). Simulations using the pwrEWAS tool indicated approximately 80% power to detect this number of associations in females and 85% power to detect this number of associations in males, using our significance and effect size thresholds (see Methods)^51^. The total number of differentially methylated CMRs passing these thresholds in each sex was higher than expected by chance (female enrichment *p*_adj_ = 0.006, male enrichment *p*_adj_ = 0.022, 1000 permutations).

**Table 1:** Top 25 differentially methylated CMRs in females from TERRE (ranked by adjusted *p* value)

CMR coordinates	CMR length (bp)	Number of EPIC probes in CMR	Gene(s)	Gene feature(s)	*p* value	BH-adjusted *p* value	Adjusted Δβ
chr15:100913934–100913949	15	2			4.02 × 10^−7^	0.003	−0.031
chr13:25506131–25506384	253	4	*TPTE2P1*, *LOC646405*	Body	1.36 × 10^−^^6^	0.003	−0.071
chr16:1217652–1217858	206	2	*CACNA1H*	Body	1.51 × 10^−^^6^	0.003	0.047
chr11:22454152–22454662	510	6			6.46 × 10^−^^6^	0.007	0.039
chr11:19529541–19530094	553	2	*NAV2*, *NAV2–AS5*	TSS1500, Body	5.97 × 10^−^^6^	0.007	0.031
chr20:62865953–62866093	140	3	*MYT1*	Body	6.44 × 10^−^^6^	0.007	0.034
chrX:47419673–47419691	18	2	*ARAF*	TSS1500	6.83 × 10^−^^6^	0.007	0.048
chr7:25608634–25608674	40	2			1.03 × 10^−^^5^	0.009	0.046
chr10:3282437–3282651	214	3			1.42 × 10^−^^5^	0.010	0.093
chr18:77280264–77280587	323	3	*NFATC1*	Body	1.89 × 10^−^^5^	0.010	0.064
chr19:57630202–57630662	460	10	*USP29*	TSS1500	2.34 × 10^−^^5^	0.011	0.054
chr19:57630691–57630711	20	2	*USP29*	TSS1500	3.12 × 10^−^^5^	0.013	0.049
chr3:137228231–137228637	406	3			3.47 × 10^−^^5^	0.013	0.046
chr14:99641017–99641152	135	2	*BCL11B*	Body	3.94 × 10^−^^5^	0.014	0.078
chr6:160023581–160024145	564	6			4.11 × 10^−^^5^	0.014	0.107
chr16:80351660–80351734	74	2	*LOC102724084*	Body	4.13 × 10^−^^5^	0.014	0.061
chrX:145509155–145509522	367	2			4.24 × 10^−^^5^	0.014	0.035
chr7:100701511–100701518	7	2	*MUC17*	3’UTR	4.90 × 10^−^^5^	0.015	0.072
chr10:1531243–1531530	287	4	*ADARB2*	Body	5.73 × 10^−^^5^	0.016	0.034
chr5:34494278–34494484	206	2			5.81 × 10^−^^5^	0.016	0.038
chr11:75142012–75142450	438	2	*KLHL35*	TSS1500	7.51 × 10^−^^5^	0.018	0.038
chr12:95226786–95226994	208	2	*MIR492*	TSS1500	9.03 × 10^−^^5^	0.019	−0.082
chr15:33023237–33023587	350	2	*GREM1*	Body	9.60 × 10^−^^5^	0.019	0.067
chr19:18888081–18889004	923	3	*CRTC1*	Body	1.03 × 10^−^^4^	0.019	0.094
chrX:107306969–107307863	894	3	*VSIG1*	Body	1.20 × 10^−^^4^	0.021	0.034

*n* = 33 cases, 67 controls.

*BH* Benjamini–Hochberg; bp, base pairs, *CMR* comethylated region, *adjusted Δβ* difference in DNAm between PD cases and controls, adjusted for age, head trauma, alcohol consumption, smoking, cell type PCs 1–6, and genotype PCs 1–3.

**Table 2:** Differentially methylated CMRs in males from TERRE

CMR coordinates	CM length (bp)	Number of EPIC probes in CMR	Gene(s)	Gene feature(s)	*p* value	BH-adjusted *p* value	Adjusted Δβ
chr19:17433773–17434268	495	2	*ANO8,DDA1*	3’UTR	1.17 × 10^−^^6^	0.019	0.047
chr6:32294470–32295230	760	5	*C6orf10*	Body	6.04 × 10^−^^6^	0.039	0.038

*n* = 38 cases, 80 controls.

*BH* Benjamini–Hochberg, *bp* base pairs, *CMR* comethylated region, *adjusted Δβ* difference in DNAm between PD cases and controls, adjusted for age, head trauma, alcohol consumption, smoking, cell type PCs 1–6, and genotype PCs 1–3.

In the analysis in females only, CMR size ranged from 7–2606 base pairs (bp), covering 2–13 EPIC probes (average 368 bp in length, 3 probes per CMR) (Table 1, Supplementary Table 1). Differentially methylated CMRs in females mapped to 50 genes, including cell signaling proteins (e.g., *ARAF*, *ST5*, *DLGAP1*), transcription and translation regulators (e.g., *FRX2*, *NFATC1*, *CRTC1*), and ion and nucleotide transporters (*CACNA1H*, *SLC35A1*, *SLC1A7*, and *SLC25A18*) (Table 1, Supplementary Table 1). In the analysis in males only, CMRs were 495 and 760 bp in length, covering 2 and 5 probes, respectively (Table 2). One of the two differentially methylated CMRs in males mapped to the *ANO8* (Cl^−^ transport) and *DDA1* (polyubiquitination) genes, while the other was annotated to *C6orf10* (encoding a multifunctional, ubiquitously expressed protein) (Table 2). No differentially methylated CMRs overlapped between the female- and male-specific analyses, and all CMRs differentially methylated by PD status in females except for three (chr3:137228231–137228637, *SPO11*, and *ASRGL1*) had little or no effect in the opposite sex (Supplementary Fig. 2). When males and females were combined in an additive model, adjusting for the same covariates as well as for sex, only the chr3:137228231–137228637 CMR met significance and effect size thresholds; other CMRs had correlated DNAm patterns between sexes, with median Δβ_adj_ < 0.03 (Supplementary Fig. 3). Overall, these results indicated that more PD-associated differential DNAm was detectable in females from TERRE than in males.

### A subset of PD-associated differentially methylated CMRs in females from TERRE replicated in independent populations

In order to explore the underpinnings of variance in DNAm at PD-associated differentially methylated CMRs, we conducted replication analyses in three independent samples with differing demographics (PEG1, DIGPD, and SGPD). To test for replication and to examine overall concordance in the magnitude of PD-associated DNAm differences at a larger number of regions, we assessed whether median Δβ_adj_ values for CMRs meeting *p*_adj_ ≤ 0.05 in TERRE (508 CMRs in females, 7 CMRs in males) were correlated across samples without imposing an effect size cutoff^52,53^. As the PEG1 study was most similar to TERRE, including individuals with shorter disease duration living in agricultural regions, we expected DNAm patterns in this sample would have the strongest correlation with DNAm patterns observed in TERRE. CMR Δβ_adj_ values were weakly correlated between TERRE and PEG1 females (*r* = 0.14, *p* = 0.075), and uncorrelated between TERRE and SGPD females (*r* = −0.096, *p* = 0.24) (Supplementary Fig. 4).

**Fig. 5:**
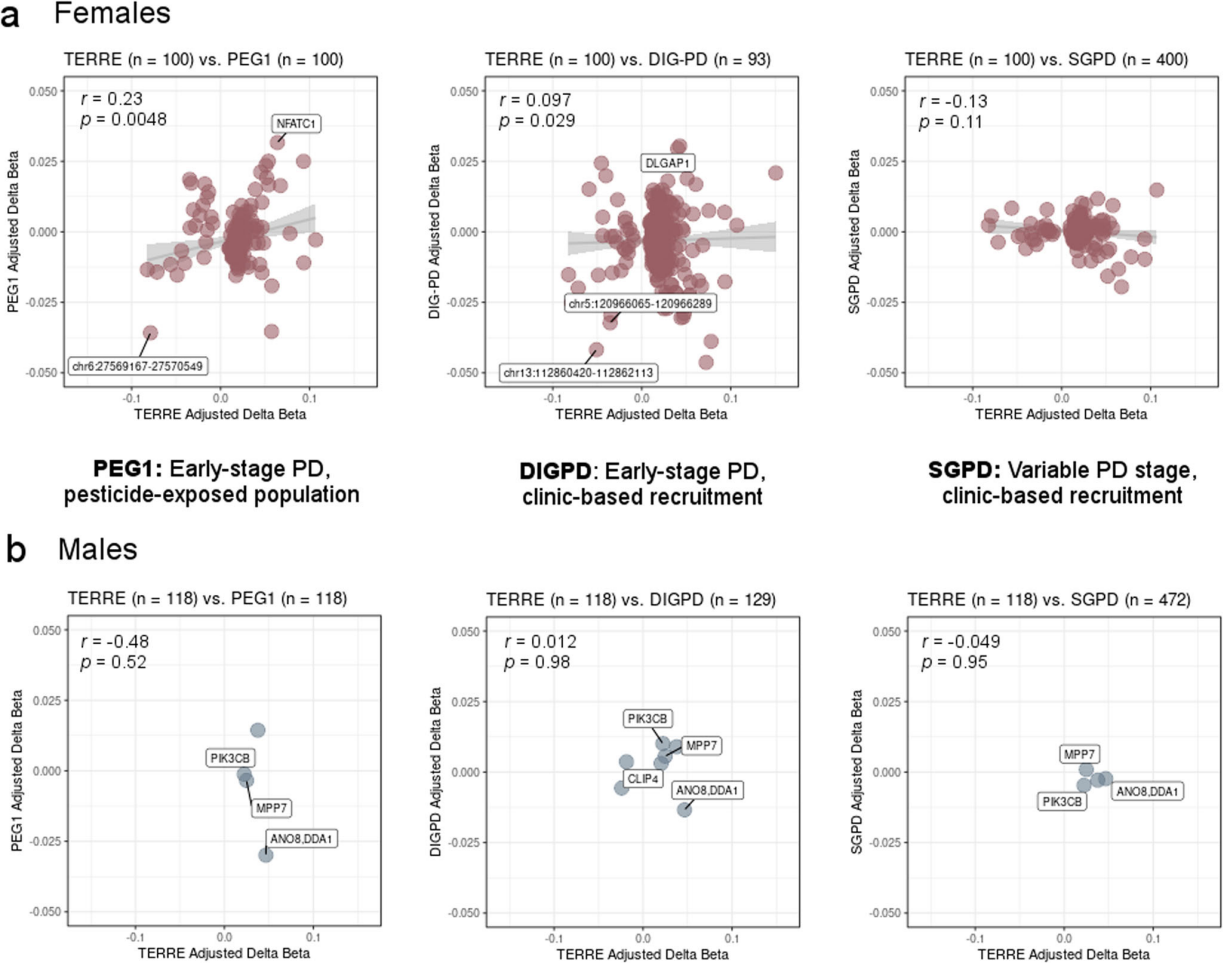
Correlation of PD-associated blood DNA methylation patterns in TERRE and other populations after propensity matching. CMRs that passed *p*_adj_ ≤ 0.05 in TERRE epigenome-wide association analyses in each sex are shown (508 total in females: 155/508 covered in PEG1 and SGPD 450 K array datasets, 506/508 covered in DIGPD; 7 in males: 4/7 covered in PEG1 and SGPD, 7/7 in DIGPD). *x* axis: median CMR |Δβ_*adj*_| in individuals from TERRE; *y* axis: median CMR |Δβ_*adj*_| in individuals from PEG1 (matched for age, predicted smoking, and predicted neutrophil proportion), DIGPD, or SGPD (matched for age, predicted smoking, and predicted neutrophil proportion). Pearson’s correlation coefficients (*r*) and *p* values are shown. **a** Left to right: Δβ_*adj*_ correlations in females from PEG1, DIGPD, and SGPD. **b** Left to right: Δβ_*adj*_ correlations in males from PEG1, DIGPD, and SGPD.

As overall covariate balance (age, predicted smoking, and predicted neutrophil proportion) differed between the PD cases and controls in each replication sample (standardized mean difference (SMD) 0.57–0.86) and between each replication sample and TERRE (SMD 0.79–2.37), we next assessed the effects of propensity matching in order to reduce potential bias related to this imbalance (final *n* = 218 matched individuals in PEG1, *n* = 872 SGPD; SMD 0.54–1.15; Supplementary Table 2). In matched females, Δβ_adj_ values were positively correlated between TERRE and PEG1 (*r* = 0.23, *p* = 0.0048) and between TERRE and DIGPD (*r* = 0.097, *p* = 0.029) and showed no correlation between TERRE and SGPD (*r* = −0.13, *p* = 0.11) (Fig. 5a). Of the 69 CMRs differentially methylated at |Δβ_adj_ | ≥ 0.03 in TERRE, 62% of those also covered by the Illumina 450 K array (26/42) had the same effect direction in PEG1, higher than expected by chance (*p*_adj_ = 0.014, 1000 permutations), as compared with 55% and 46% of CMRs with the same effect direction in either SGPD or DIGPD, no greater than expected by chance (*p*_adj_ > 0.41, 1000 permutations).

Finally, we considered individual CMRs as replicated if they were differentially methylated in the same direction and at a similar magnitude to TERRE (|Δβ_adj_| ≥ 0.03) in at least one other replication sample. Two of the 26 CMRs with the same effect direction in PEG1 had |Δβ_adj_| ≥ 0.03 (*NFATC1*, chr6:27569167–27570549), as did five of the 32 CMRs with the same effect direction in DIGPD, including three EPIC array-specific CMRs (EPIC/450 K shared: *MPDU1*, chr5:2334885–2335317; EPIC-specific: *DLGAP1*, chr5:120966065–120966289, chr13:112860420–112862113; Fig. 5a, Supplementary Table 3). In males, Δβ_adj_ values were uncorrelated between TERRE and all three matched replication samples (*r* = −0.48–0.012, *p* = 0.52–0.98), no CMRs had |Δβ_adj_| ≥ 0.03 in the same direction as TERRE, and either one or neither CMR with |Δβ_adj_| ≥ 0.03 in TERRE had the same sign in TERRE and the replication samples, no greater than expected by chance (Fig. 5b, Supplementary Table 4, *p*_adj_ > 0.72, 1000 permutations).

### Genetic variation explained more variation in DNAm at PD-associated CMRs than exposure to pesticides

To assess additional sources of variation in DNAm at PD-associated CMRs and elucidate potential reasons for the varying concordance of changes in DNAm at CMRs across data sets, we next determined whether adding additional covariates to the models could better explain DNAm at PD-associated CMRs in TERRE. We focused on PD-associated CMRs from the female-stratified analysis, highlighting CMRs that replicated in at least one other study.

Noting that both PD and variation in DNAm are affected by genetics and environment, we explored how accounting for genotype (G), exposure to pesticides (E), their additive effect (G + E), and their interaction (G×E) could explain variation in DNAm at PD-associated CMRs in TERRE, correcting for multiple testing accordingly (Fig. 3b). This can be ascertained by ranking the relative fit of models including G, E, G + E, or G × E terms as covariates explaining variance in DNAm at each CpG or region^36,37^. Of note, this approach is distinct from exploring the overall contributions of genotype and exposure to genome-wide DNAm patterns in TERRE, which can be ascertained by examining each covariate as a main effect and considering all CMRs and/or CpGs passing quality control (QC) as opposed to only PD-associated CMRs (Supplementary Table 5).

**Fig. 6:**
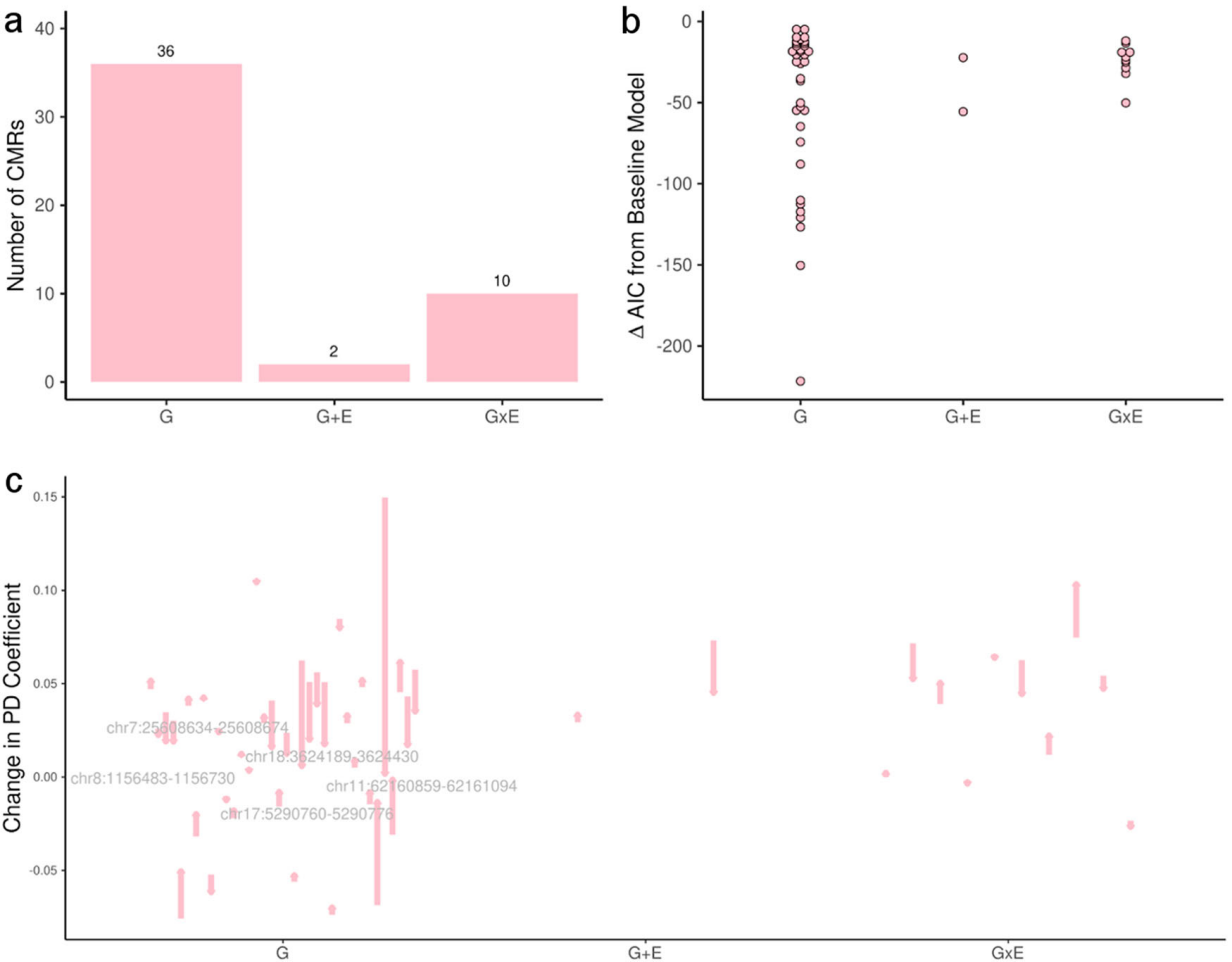
Sensitivity of PD CMR DNAm to genetic and exposure variables. CMRs that passed *p*_adj_ ≤ 0.05 and median CMR |Δ*β*_adj_| ≥ 0.03 in TERRE epigenome-wide association analyses in females were fit to an exposure (E), genetic (G), additive (G + E), or interactive (G×E) model. **a** Number of CMRs with a minimum AIC corresponding to each model. **b** Minimum AIC value corresponding to the minimum AIC model for each CMR. **c** Changes in effect of PD on DNAm (Δβ_adj_) corresponding to the minimum AIC model for each CMR, relative to the base model. Arrows show the direction of change for each PD effect, and CMRs with a PD effect that changed by ≥0.03 are labeled.

For G, we considered each SNP within 75 kb of each PD-associated CMR, and for E, we considered each available pesticide with at least 10% of individuals exposed on average across imputations within each sex. This included insecticides, fungicides, and overall exposure to pesticides during gardening in females (Supplementary Table 6). For each CMR, we ranked all models (baseline, baseline + G, baseline + E, baseline + G + E, or baseline + G + E + G:E) by their Akaike Information Criterion (AIC) value, which explained the goodness of fit for each model adjusting for the number of covariates included in each model (Fig. 6a, b; Table 3; Supplementary Table 7). Of the seven replicated CMRs in females, six showed significant improvement over the baseline, four of which were best explained by a G-only model (*NFATC1*, *DLGAP*, chr13:112860420–112862113, chr5:2334885–2335317) and the two of which were explained by a G × E model with respect to exposure to fungicides (*DLGAP1*, chr18:77280264–77280587) (Table 3). Across all CMRs differentially methylated in females, 48 CMRs (70%) showed significant improvement over the baseline, and were best explained by models including G either alone (36 CMRs, 52%) or including G in an additive or interaction model with pesticide exposure (12 CMRs, 17%; Supplementary Table 7). SNP genotype at the *RARS2*, *LOC286083, LOC646588, ANKLE1, CACNA1H, GRAMD2B, P2RX2, RPS3*, and *TPTE2P1* genes explained DNAm patterns at three or more PD-associated CMRs each in females (Supplementary Table 7). Of the 12 CMRs influenced by an exposure in females, two were best explained by a G + E model (*CRTC1* and chr2:10,637,974–10,638,073 influenced by overall gardening-level exposure; Supplementary Table 7), and ten were best explained by a G×E model with either overall gardening-level exposure (six CMRs), exposure to fungicides (three CMRs), or exposure to insecticides (one CMR) (Supplementary Table 7). To distinguish the overall associations of pesticide exposure with DNAm in TERRE from the contributions of pesticide exposure to PD-associated CMR model fit in TERRE, we also ran sex-stratified EWAS with overall pesticide exposure as the main effect; as expected, more associations of pesticide exposure with DNAm in males than in females were uncovered when considering CMRs and CpGs regardless of their association with PD status (Supplementary Table 5).

**Table 3:** Summary statistics of the minimum AIC G × E models for PD-associated CMRs replicated in females

CMR coordinates	CMR gene (s)	SNP	Nearest gene to SNP	Exposure	Δβ (E)	Δβ (G)	Δβ (G × E)	AIC	ΔAIC vs. base model	Change in |Δβ_adj_|(PD) vs. base model	Adjusted *p* value (*F* test)
chr18:3624189–3624430	*DLGAP1*	rs12604364	*DLGAP1-AS2*	n/a	n/a	0.10	n/a	−300	−110	−0.033	1.41 × 10^−15^
chr18:7728026–77280587	*NFATC1*	rs79426764	*DLGAP1−AS2*	Fungicide	1.27	−0.14	−0.63	−164	−50	−0.018	1.22 × 10^−^^7^
chr6:27569167–27570549	n/a	rs10807026	*RARS2*	Fungicide	0.09	−0.03	−0.14	−261	−29	0.002	6.57 × 10^−^^6^
chr5:2334885–2335317	n/a	rs2628156	*GRAMD2B*	n/a	n/a	−0.04	n/a	−244	−15	0.004	2.65 × 10^−^^3^
chr13:112860420–112862113	n/a	rs200658161	*TPTE2P1*	n/a	n/a	−0.06	n/a	−200	−12	−0.010	2.61 × 10^−^^2^
chr5:120966065–120966289	n/a	rs72793312	*GRAMD2B*	n/a	n/a	0.03	n/a	−334	−10	−0.002	4.30 × 10^−^^2^

*n/a* an effect not estimated in the minimum AIC model for a given CMR, *Δβ (E)* coefficient for E (pesticide exposure) term, *Δβ (G)* coefficient for G (SNP genotype) term, *Δβ (G×E)* coefficient for the interaction between G (SNP genotype) and E (pesticide exposure).

Next, we assessed how the observed contribution of PD status to DNAm level at each CMR changed when this effect was considered independently of underlying G, G + E, or G × E effects. To do so, we selected the model with the minimum AIC for each CMR and assessed the change in PD effect on DNAm when accounting for the SNP or SNP−exposure pair underpinning the model (Fig. 6c; Table 3; Supplementary Table 7). In replicated CMRs, the magnitude of the |Δβ_adj_| with respect to PD status increased for two CMRs (chr5:2334885–2335317, a change of 0.004, and chr6:27569167–27570549, a change of 0.002; Table 3). The remaining five replicated CMRs showed a decrease in |Δβ_adj_|, ranging from 0.002–0.033 (Table 3). Considering all CMRs differentially methylated in females from TERRE, we observed a similar change in |Δβ_adj_| with respect to PD status: this was decreased for the majority of CMRs (30 of 48 CMRs (63%)), albeit with generally larger changes in |Δβ_adj_| (between 0.0004–0.1481 change, average 0.0211 change). Of the CMRs showing an increased |Δβ_adj_| for PD status the increase was modest, with maximum increases of 0.0287 at *MUC17* accounting for a G × E model with the SNP rs112404953 and gardening-level exposure, 0.0163 at *SLITRK5* accounting for the G model of rs954384, 0.0114 at *BBOX1* accounting for a G × E model with the SNP rs10742165 and gardening-level exposure, and 0.0104 at *MIR7-3HG* accounting for a G × E model with the SNP rs7257678 and gardening-level exposure; the remainder of increases were less than 0.01 (Supplementary Table 7). Therefore, G effects generally explained part of the variation in DNAm at CMRs previously attributed to PD, suggesting that these factors contributed to the association between PD and DNAm. Moreover, no SNPs within a 1-Mb window of each of these CMRs showed evidence of association with PD in an independent GWAS, indicating that it is unlikely the genetic effects found here were simply explaining PD status^29^.

### CMR DNAm was not associated with levodopa dosage in female PD patients but showed inconsistent sensitivity to lag time between pesticide exposure and sample collection

To rule out further potential sources of confounding, we performed sensitivity analysis for female PD-associated CMRs in TERRE regarding levodopa daily dosage (LED) and time since pesticide exposure. Twenty-three of 33 female PD patients (70%) were receiving levodopa, and of these, 22 patients had LED information available. For each of the 69 PD-associated CMRs in females, the model CMR median β ~ LED + age + plate + cell type PC1 was run, using data from the 22 patients (Supplementary Methods). No CMRs had a median β associated with LED (all *p*_adj_ > 0.4).

We also assessed whether accounting for the lag time between pesticide exposure and blood sample collection affected which model best explained variation in DNAm for PD-associated CMRs in females. We assessed the effect of exposures less than the median lag time across samples (i.e., recent exposure) along with the effect of exposures greater than or equal to the median lag time across all samples (i.e., past exposure, Supplementary Methods). G models were still top-ranked for explaining CMR DNAm when accounting for lag time in the majority of cases (31 of 36 CMRs where G was originally top-ranked, or 86%). The five remaining CMRs that originally had a top-ranked G model were better explained by G × E (chr10:1531243–1531530, chr13:88328009–88330615, gardening, with respect to different SNPs than in their top G model; chr13:25506131–25506384, chr15:35086890–35086986, fungicide, both with respect to the SNP in their previous top G model) or G + E models (chr6:160023581–160024145, fungicide, with respect to the same SNP in its previous top G model; Supplementary Tables 7 and 8). This likely resulted from better fits of recent or past pesticide exposure with these models. Neither lag time was consistently better at predicting changes in DNAm (Supplementary Table 8).

Of the ten CMRs originally best explained by a G × E model, five were recovered when accounting for lag time, two were better explained by a G only model (chr18:77280264–77280587, rs2330761 and chr7:54955929–54956420, rs2330761), and the remainder no longer significantly improved over the baseline model. Of the recovered G × E models, two were better explained by a different exposure and SNP than in the original analysis (chr11:22454152–22454662, fungicide, rs201451547 and chr11:27076803–27076820, fungicide, rs11499776), and two were better explained by the same exposure and a different SNP than in the original analysis (chr19:57630691–57630711, fungicide, rs28416079, and chr5:34494278–34494484, gardening, rs10051815). Only the chrX:47419673–47419691 CMR remained best explained by the same G × E model as in the original analysis (insecticides and rs3748517). One G + E model, at chr19:18888081–18889004, remained best explained by gardening-level exposure and genotype, but with respect to rs117360667.

## Discussion

The discovery of molecular signatures of early-stage PD is important in order to elucidate interindividual etiology, and to inform prevention and intervention strategies to slow PD onset and/or progression. Our understanding of blood DNAm changes associated with PD is still unclear, as variation in DNAm can be highly population-specific. In this study, we determined regional, sex-specific blood DNAm signatures of early-stage PD in TERRE, and assessed the influences of genotype and pesticide exposure on these PD-associated molecular signatures. We demonstrated a subset of differentially methylated CMRs in females that were robust when examined in demographically matched populations. Additionally, genetic variation *in cis* explained part of the association between DNAm and PD, with some potential contribution of pesticide exposure to this relationship. Collectively, our results illustrate the complex nature of DNAm changes associated with PD, and emphasize the importance of taking genetic variation into consideration in future PD EWAS.

In addition to neurological changes, PD and pesticide exposure are related to changes in immune cell composition and inflammation, which can be observed in blood^4^^,54^. Therefore, blood-based DNAm alterations in early-stage PD could partially reflect immune-based processes associated with disease pathogenesis. However, as predicted immune cell composition was unaltered between cases and controls in TERRE, lifestyle factors or exposures unique to the sample may have also influenced blood cell type composition. Along these lines, one of the CMRs which replicated only in the pesticide-exposed sample, PEG1, mapped to the *NFATC1* gene, which encodes a transcription factor controlling cytokine expression in T cells during immune activation^55^. A second CMR that replicated in DIGPD was annotated to the *DLGAP1* gene, which encodes an adaptor protein found at glutamatergic synapses and may also be of interest for follow-up experiments. Aside from the replicated CMR genes, the full list of PD-associated CMRs in females from TERRE included additional genes which could have relevance for PD: *CRTC1*, which has been reported as differentially methylated in prefrontal cortex (PFC) neurons from PD patients and whose protein product is involved in mitochondrial biogenesis; *SLC1A7*, encoding a glutamate transporter; *FXR2*, whose protein product is involved in DNA damage response; and *ST5*, SNPs of which are associated with PD progression, and whose protein product is involved in endosomal trafficking^56–59^. We note these potential links to PD will require further experimental work to validate, as our study was not designed to causally assess disease mechanisms.

In order to increase the chances of discovering biologically relevant changes in DNAm in this study, we chose to examine PD-associated DNAm patterns at blood-specific reference CMRs, which have spatially correlated DNAm patterns that are consistent within individuals^19^. Focusing on reference CMRs allowed us to assess DNAm patterns at regions that are co-methylated in blood across different populations; however, it should also be noted that the reference CMRs captured only 35% of the total CMRs called uniquely in TERRE (35,907 of 102,723 CMRs with 1 or more probes in a reference CMR). Similarly, the reference CMRs covered approximately 30% of the CpGs and regions previously identified as differentially methylated in the blood of PD patients, none of which were differentially methylated in TERRE^10,12–15,18^. This was not unexpected due to the reduced CMR testing space, low levels of replication reported previously for PD EWAS, and variations in disease duration, cell type composition, and demographics of the samples compared in this study^12^. For instance, obtaining detailed information on demographic variables affecting both blood DNAm and PD risk such as head trauma and alcohol consumption and accounting for these variables in PD epigenetics studies may improve reproducibility. Along these lines, our comparison of CMR effect sizes between samples indicated that similar PD-associated DNAm trends were observed for some regions when these effects were accounted for and when study populations were more closely matched (i.e., PEG1, including early- to mid-stage patients exposed to pesticides, and DIGPD, including early-stage patients also recruited in France). Despite the lack of overlap with previous EWAS in the blood of PD patients, several CMRs differentially methylated in the females analysis mapped to genes previously reported as differentially methylated in PFC neurons from PD patients (*NAV2*, *CRTC1*, *NTSR1*, *ADARB2*), and/or in brain tissue from PD patients (*CACNA1H*, differentially methylated in cingulate gyrus)^17,56^. Of note, both brain-based studies employed region-based approaches to differential methylation analysis, which also may have influenced reproducibility of the findings. Overall, our results and replication analyses indicated that whether specific CpGs and regions are called as differentially methylated in each study is dependent on a variety of factors, including demographics, sample size, statistical model, and approach to differential DNAm analysis (including site-specific vs. regional, and criteria used to define regions).

Sex and gender also influence DNAm patterns and may impact findings of PD-associated DNAm changes, through biological and/or sociocultural mechanisms^44,60–63^. Considering that the incidence of PD in men is nearly twice that in women, it was unexpected that more DNAm changes were found in the blood of female patients (69 CMRs) than male patients (2 CMRs) and at similar magnitudes in the TERRE sample^64^. However, this was consistent with a recent sex-stratified EWAS in the parietal cortex of PD patients where 3 PD-associated CpGs were reported in men, and 87 in women^49^. Similarly, it was somewhat surprising that pesticide exposure, and in particular low-dose exposure, contributed to CMR model fit in females, considering that male TERRE subjects had higher levels of pesticide exposure (i.e., occupational). This can partly be explained by our restriction of the G × E analysis to only PD-associated CMRs, a greater number of which were observed in females. When PD status and genotype were not considered, i.e. when pesticide exposure was used as the variable of interest in a separate EWAS, exposure effects were indeed detected in males, suggesting that the effects of pesticide exposure as a whole and the effects of exposure converging with PD and genotype were distinct. The three-category pesticide exposure variable also does not capture all routes of exposure that could be common in females in this study, including exposures related to household tasks (e.g. handling contaminated objects and clothes, inhalation through dust), drift-related environmental exposure, and accumulation of prior exposures in adipose tissue^46–48^. These routes of exposure are challenging to quantify and often under-estimated. Along these lines, associations of low-dose (ambient) pesticide exposure with blood DNAm levels have been reported in PD patients and healthy controls, as well as female-specific associations of overall pesticide exposure with blood DNAm levels in women^33–35^. Taken together, a range of biological, lifestyle, and exposure factors influence sex- and gender-related DNAm patterns and sex and gender differences in PD susceptibility, presentation, and progression. Our results supported existing calls to consider both biological underpinnings of sex as well as the interactions between sex/gender and lifestyle/exposure in epigenetic studies of PD and of other phenotypes^44,65^^,66^.

To better understand our ability to detect G × E effects in this study regardless of sex, we looked to recent expression quantitative trait loci (eQTL) literature, which would more accurately reflect the associations we expected to find. Several eQTL studies have detected G × E effects with sample sizes close to the number of female subjects in this study^67–69^. Exposure to the specific pesticides underpinning the G × E effects observed in TERRE (insecticides and fungicides) has also been associated with PD risk in several epidemiological and experimental studies^50,70–74^. Although our overall G × E results in females from TERRE were consistent with these studies, our lag time sensitivity results alluded to the importance of accurately measuring pesticide exposure with respect to both recency of exposure and overall level of exposure in future epigenetic studies of PD.

Finally, genetic variation was a major contributor to variation in DNAm at PD-associated CMRs in this study, as G models frequently out-ranked G + E and G × E models, and no models including E alone showed improvement over the baseline. This was consistent with previous G × E studies in cord blood and cord tissue, where E effects were also observed primarily when included with genotype in either additive or interaction models, and with the reported contribution of SNP genotype to DNAm (estimated to influence 20–80% of overall DNAm variation)^20,36,37,75^^,76^. In the specific context of pesticides, impacts of common genetic variation (*GSTP1*, *ABCB1*) and PD-associated mutations (*LRRK2* G2019S) on response to herbicide, organochlorine, and paraquat exposure have been identified in both human and model organism studies^77–80^. While we identified different SNPs associated with pesticide exposure in this study, it is still possible they may have influenced response to exposure, which could be resolved with more precise accounting of exposure and supported by experimental studies^73^. On the whole, the lack of association between SNPs explaining DNAm at PD-associated CMRs in TERRE and SNPs associated with genetic risk for PD in populations of European ancestry suggested that SNP genotype may impact DNAm levels at the given CMRs independently of PD status (for instance, if PD cases and controls had different genotypes by chance), and/or that genotype and PD could both be associated with additional unmeasured factors, such as population-specific G × G or G × E interactions^29^^,76^. This is important to consider when designing and interpreting genetic and epigenetic association studies for PD, as common genetic variation may confound associations between disease status and DNAm. Genetic variation can be incorporated into EWAS of PD through several approaches, such as examining whether CpGs differentially methylated in PD patients have associated methylation quantitative trait loci (mQTL), conducting Mendelian randomization analysis on candidate CpGs to assess potential causal relationships between DNAm and phenotypic outcomes mediated by genetic variation, or quantifying the contribution of genotype to PD-associated DNAm patterns when matched genetic and epigenetic data is available^12,24,81^. Obtaining detailed measurements of environments, lifestyle factors, and exposures influencing DNAm and PD risk, ideally in a quantitative manner, will also facilitate exploration of G × E effects on DNAm and PD, an area which would benefit from further methodological development.

In this study, we showed that DNAm changes were detectable in early-stage PD, and were largely influenced by demographics and *in cis* genetic variation. As epigenetic associations were seen so early in disease progression, blood DNAm could potentially reflect residual immune cell composition changes remaining after adjustment, and/or be one mechanism relating common genetic variation to early molecular changes associated with sporadic PD^82^. Despite these insights, several limitations of our study should be taken into consideration. First, while the sample size was small for detecting G × E interactions, the TERRE sample presented a unique opportunity to assess the effects of genotype and pesticide exposure on DNAm in the same individuals^83^. Second, the level of exposure and manner in which it was measured differed between TERRE and PEG1. Published studies assessing the effects of pesticide exposure on DNAm in PEG1 focused on ambient exposures (organophosphate, pyrethroid) assessed via geographic information systems, which were not measured here, and could have a different impact on DNAm than gardening or occupational pesticide exposure^34,35^. Additionally, recall bias may have affected pesticide exposure ascertainment in TERRE and the comparison with PEG1, as household and occupational exposures were self-reported in both samples^70^. However, our study adds to the literature on how pesticide exposure impacts DNAm, whether this differs by sex, and the degree to which it is associated with PD. Third, due to the differences in exposures between males and females in TERRE and since all individuals were cisgender, we cannot conclude definitively whether the sex-specific DNAm patterns observed in this study were due to biological sex differences or were related to exposure history or other sociodemographic discrepancies. Finally, we were unable to test the joint impacts of genotype and pesticide exposure on DNAm in the replication samples, as none had detailed information on both of these variables collected for cases and controls. Despite this, we observed improved replication of results upon propensity matching, illuminating the population specificity of PD-associated regional DNAm changes.

The results presented here support an association of genetic variation with DNAm patterns in early-stage sporadic PD in a unique agricultural population with detailed pesticide exposure history. By characterizing relevant genetic and exposure-related sources of variation in PD-associated blood DNAm, we highlighted the population specificity of PD-related DNAm patterns and the need for careful consideration of confounders in PD EWAS. On the whole, our results emphasize the importance of assessing epigenetic variation in the context of genetic background in future investigations.

## Methods

### Study samples and design

Identification of sex-specific DNAm signatures of PD was performed in the TERRE case-control study consisting of individuals enrolled in the Mutualité Sociale Agricole (MSA) health insurance system for French agricultural workers and their spouses, as described previously^50,84^ (Figs. 1 and 3). Cases were selected from MSA-enrolled individuals who developed PD. Controls were selected from individuals free of parkinsonism and enrolled in the MSA who applied for healthcare reimbursements between February 1998 and 2000. A maximum of three controls per patient were selected randomly from groups of the same sex, age (±2 years), and MSA affiliation office. Of a total of 224 PD patients and 557 controls, 71 patients with a short PD duration (≤1.5 years) and 147 controls for whom both genotyping and whole blood DNAm data were available and passed quality control checks were included in this study (*n* = 33 female PD cases, 67 female controls; 38 male PD cases, 80 male controls; 99% (215 of 218 participants) self-reported White; 1% (3 participants) self-reported North African) (Fig. 1, Supplementary Table 9). This represented a sample of patients with very short disease duration, as the median survival in PD is estimated to be of 13.4 years^85^.

A replication of sex-stratified differential DNAm analysis in whole blood was conducted in DIGPD, a longitudinal sample of 411 French patients with PD (duration ≤5 years at baseline) followed up annually for 7 years (Fig. 1)^28,86,87^. To examine the replicability of DNAm changes associated with early-stage PD identified in the TERRE sample in patients with the earliest stages of PD from DIGPD, individuals with disease duration ≤2 years at baseline and age- and sex-matched PD-free controls were selected for DNAm profiling on the EPIC BeadChip array^86^. After excluding individuals with monogenic PD mutations (5 with GBA E326K and 2 with LRRK2 G2019S), a total of 110 patients were included in the analysis (94% (104 of 110 patients) self-reported White; 3% (3 patients) self-reported North African; 3% (3 patients) self-reported Black, Asian, or Latino). These patients were compared to 112 controls, for whom extended demographic information beyond age and sex was not available. Considering PD patients only, the male:female ratio, proportion of smokers, and self-reported race distribution was similar between TERRE and DIGPD (Supplementary Table 10). However, PD patients from DIGPD had less pesticide exposure, fewer individuals on levodopa or dopamine agonist medications, and more education on average compared to PD patients from TERRE (Supplementary Table 11). Detailed information on levodopa and dopamine agonist treatment for PD patients from DIGPD was previously published^87^.

Additional replication analysis was conducted with publicly available whole blood DNAm data from PEG1, a population-based study of PD patients (*n* = 335) and controls (*n* = 237) recruited from agricultural areas in central California between 2000 and 2007 (89% (508 participants) self-reported White; 11% (64 participants) self-reported Hispanic; Fig. 1)^15,88^ (GSE111629). Participants were recruited by neurologists, public service announcements, and local medical centers, and had lived in California for at least 5 years prior to recruitment. Only PD patients with a disease duration of 3 years or less were included. The PEG1 study had a higher proportion of smokers, and a similar male:female ratio and pesticide exposure level to TERRE (Supplementary Table 10).

Replication was also assessed using publicly available whole blood DNAm data from the SGPD sample (*n* = 1292 cases, 1041 controls), composed of participants of European descent from three studies conducted in Australia and New Zealand: the Queensland Parkinson’s Project (QPP), the New Zealand Brain Research Institute PD case-control study (NZBRI), and the Sydney PD case-control study (SYD) (Fig. 1)^12^. These studies recruited PD patients of variable disease duration (2–40+ years), with community-based volunteers as controls. The GEO data set consists of 959 PD patients and 930 controls, excluding subjects that failed QC (GSE145361). The final subset has a similar male:female ratio, lower average pesticide exposure, and higher average smoking duration than TERRE (Supplementary Table 10).

### Measures

In the TERRE sample, PD was diagnosed by a neurologist specializing in movement disorders, and was defined as the presence of parkinsonism with exclusion of drug-induced phenotypes or further nervous system involvement^89^. No PD patients had known monogenic mutations.

In the DIGPD sample, patients were diagnosed by movement disorder neurologists according to the UK Parkinson’s Disease Society Brain Bank criteria^90^. In PEG1, PD was diagnosed using the UK Brain Bank and Gelb diagnostic criteria, while in SPGD, PD was diagnosed with the Calne criteria^91–93^.

Occupational pesticide exposure in TERRE was assessed with a two-phase procedure, including a self-report questionnaire designed for this study, followed by occupational health interviews conducted at the homes of individuals exposed to pesticides via their profession to obtain detailed information on history of pesticide exposure, including the number and type of farms where the individuals had worked; which pesticides they had personally sprayed; the frequency, duration, and method of spraying; and number of years that they were exposed^50,94,95^. An overall pesticide exposure variable with 3 levels (never exposed, gardening/household exposure, or occupational exposure) was created based on self-reports and interviews with occupational health physicians. Binary (never/ever) occupational exposure variables for individual pesticides were created based on the occupational health interviews during site visits by physicians. Chemical composition was determined and coded using a pesticide dictionary (http://www.alanwood.net/pesticides). Missing values for individuals exposed to pesticides were imputed as described previously, using models including crop/animal, time period, disease status, sex, age, and mini-mental state examination (MMSE) as covariates^50^. Missingness across the 781 TERRE subjects originally analyzed ranged from 0–16% across all exposures. The average rate of exposure across these imputations in the subset of TERRE subjects with shared DNAm and genotyping measures is presented in Supplementary Table 6 (3 exposures in females: insecticides, fungicides, and gardening-level exposure).

In the TERRE sample, each participant was interviewed by a physician to record demographic data and administer the MMSE (Supplementary Table 9)^96^. Data was collected for cases and controls via questionnaires on education level, self-reported race, smoking, head trauma, and alcohol consumption, and for medication use and disease duration for PD cases. In the DIGPD sample, the above information was recorded for PD cases, and was unavailable for controls^86^. In the PEG1 sample, only participant age, sex, and ethnicity were publicly available, while in SGPD, only age and sex were available. Smoking scores were estimated in DIGPD, PEG1, and SGPD using the SSc method in the EpiSmokEr package^97,98^. For comparison, smoking scores were also estimated in TERRE with this approach, with current and former self-reported smokers scoring higher than never-smokers (Supplementary Fig. 5).

### Biological sample and data processing

Here, we conducted region-based epigenome-wide association analysis using new EPIC array whole blood DNAm data from the TERRE sample, focusing on whole blood reference CMRs discovered with the CoMeBack approach, which have correlated DNAm patterns in blood both within and across individuals^19^. To further test the replicability of CMRs showing differential DNAm between cases and controls in TERRE, we also assessed whole blood DNAm patterns in new EPIC array data from the DIGPD sample, and downloaded raw idat files for the PEG1 and SGPD whole blood DNAm data sets from GEO^12,15^.

Whole blood was collected in tubes containing ethylenediaminetetraacetic acid (EDTA), and DNA was extracted using a saline (Qiagen® kit) or phenol (LockGel® tube)-based protocol in accordance with the respective manufacturer’s recommendations. DNA was then precipitated with ethanol, washed, and resuspended in Tris-EDTA buffer. The concentration and purity were determined spectrophotometrically, and after adjusting to a concentration of 200 ng/μl the DNA was stored at −20 °C.

For the TERRE and DIGPD samples, DNAm was assessed at 853,307 CpGs using the Illumina HumanMethylationEPIC BeadChip array. The PEG1 and SGPD data sets from GEO each included DNAm data from the Illumina HumanMethylation450 BeadChip array at 482,421 CpGs (GSE111629, GSE145361).

Quality control and preprocessing of DNAm data was conducted separately in each sample assessed in this study (TERRE, DIGPD, PEG1, and SGPD). Raw idat files for all individuals in each sample were read into R 3.6 for data analysis using the minfi package^99^. As part of subject-level quality control, we confirmed clinically reported sex using principal component analysis (PCA) on X chromosome β values, percentage of missing values on the Y chromosome, and the *getSex* function from the minfi package (Supplementary Fig. 6, Supplementary Table 12)^99^. For subjects flagged for potential sex mismatches after these checks, X/Y chromosome copy number was additionally confirmed using the conumee package, and any subjects with copy number mismatches were removed (3–6 individuals per sample; Supplementary Fig. 6, Supplementary Table 12)^100^. After applying additional subject filtering procedures (summarized in Supplementary Table 12), 34 subjects were removed from TERRE (231 remaining), 64 subjects were removed from the first time point of DIGPD (278 remaining), 28 subjects were removed from PEG1 (539 remaining), and 138 subjects were removed from SGPD (1751 remaining) (Supplementary Table 12)^101^. The TERRE and DIGPD data sets were later subset to individuals with complete self-reported smoking data in TERRE, matched genotyping data, individuals from the first study time point of DIGPD (PD duration ≤2 years), and individuals without familial mutations (71 cases, 147 controls in TERRE; 110 cases, 112 controls in DIGPD; after normalization and prior to epigenome-wide association analysis) (Fig. 1). To reduce effects of unwanted sources of technical variation, functional normalization was performed in each study using *adjustedFunnorm* from the wateRmelon package^102^.

After normalization, low-quality probes, cross-hybridizing probes, and polymorphic probes were removed separately from each sample to ensure quality control at the individual CpG level (summarized in Supplementary Table 13)^103^. X and Y chromosome probes were retained to allow for the possibility of assessing sex chromosome DNAm in sex-stratified analysis. This left 803,777 probes in TERRE, 803,734 probes in DIGPD, 424,263 probes in PEG1, and 424,699 probes in SGPD.

In the TERRE and DIGPD samples, subjects were initially randomized on the EPIC BeadChip arrays by disease status but not by sex. Batch variables (plate, chip, and sample position on the chip) were correlated with sex in TERRE and DIGPD, and were thus accounted for as covariates in downstream linear regression analysis rather than adjusting the DNAm data during preprocessing, so as not to remove sex-associated variation during batch correction (Supplementary Figs. 7 and 8). For the PEG1 and SGPD samples, these batch variables could be adjusted for prior to replication analysis using the *ComBat* function from the sva package^104^. In PEG1, *ComBat* was applied to remove chip position effects; chip was not corrected for as samples were not balanced by disease status across chips. In SGPD, *ComBat* was used to correct chip and chip position effects. Plate information was not publicly available for PEG1 and SGPD.

Full details of genotyping data preprocessing are included in the Supplementary Methods. Briefly, the Illumina NeuroChip array was used to profile cases and controls. PLINK was used for preimputation QC^105^. The following preimputation steps were applied: removal of strand-ambiguous SNPs, mismatch between recorded sex and sex chromosome complement as determined on the array, a 0.05 call rate filter for SNPs, an individual missingness filter of 0.02, a minor allele frequency (MAF) filter of 0.01, and finally a heterozygosity filter removing individuals with *F*_het_ > 0.2^106^. At this stage, we computed genotype PCs, noting that 240 of 245 (98%) individuals with genotyping data were self-reported as White (Supplementary Fig. 9, Supplementary Methods). The first three PCs explained 16% of variation in total. Variants with Hardy–Weinberg equilibrium *p* < 1 × 10^−10^ were then removed. We included the X chromosome using the default PLINK encoding for X chromosome genotypes in males^75^. SNPs were imputed to the 1000 Genomes Phase 3 European reference panel using the Michigan imputation server (Supplementary Fig. 10). We kept SNPs with imputation quality *R*^2^ > 0.3, resulting in 8,354,189 SNPs for analysis.

### Statistical analysis

To assess and account for the effects of differing immune cell proportions on DNAm levels in the TERRE sample for region-based EWAS, cell type proportions were predicted in raw DNAm data using the extended EPIC array blood cell type reference, which can be used to predict proportions of 12 immune cell types (as compared with the 6 cell types included in the original IDOL library)^107^. Robust PCs of cell type were generated with the pcaCoDa function from the robCompositions package to create non-compositional variables for use in downstream linear regression^108^. The first six PCs of cell type explained 89% of the variance in cell type proportions in TERRE and were uncorrelated with PD status (*p* > 0.05, ANOVA; Supplementary Fig. 11). Due to legal requirements, the DIGPD data were analyzed on a different server without licensing to use the extended IDOL library. Therefore, for comparison between the discovery and replication samples, the original IDOL library including 6 immune cell types was used to predict cell type proportions in TERRE, DIGPD, PEG1, and SGPD^109^.

We selected covariates for EWAS in TERRE using a combination of data-driven and literature-informed approaches. In order to evaluate potential contributors to variance in DNAm in TERRE, we applied PCA to pre-processed DNAm data and assessed the correlation of demographic variables, predicted cell type proportions, genotype PCs, and batch variables (plate, chip, and position) with the first 10 DNAm PCs (Supplementary Fig. 12). Age, smoking, head trauma, alcohol consumption, overall pesticide exposure, cell type proportions, and batch variables were each associated with the first 2 DNAm PCs, while genotype PCs 1–3 were associated with DNAm PCs 6 and 10 (*p*_adj_ ≤ 0.05, ANOVA). We considered age, cell type PCs 1–6, plate, chip position, and genotype PCs 1–3 as core covariates for differential methylation analysis due their contribution to variance in the TERRE data as well as known contributions of these factors to DNAm variance in general, based on literature^30,110,111^. As the second stage of our analysis was focused on contributions of pesticide exposure to model fit at PD-associated CMRs, we adjusted CMR regression models for additional non-exposure variables in our PD EWAS. The fits of models also including smoking, head trauma, and alcohol consumption were evaluated in a stepwise manner within each sex using the variance inflation factor (VIF), AIC, *p*-value histograms, and quantile-quantile (Q-Q) plots (Supplementary Figs. 13 and 14, Supplementary Tables 14,15). For comparison, we additionally ran sex-stratified EWAS where pesticide exposure was the main effect, evaluating covariates via the same metrics (VIF, AIC, *p*-value histograms, and Q-Q plots; Supplementary Table 5).

Due to known sex-related differences in PD, autosomal DNAm, and PD-associated exposures between males and females, we employed a sex-stratified approach for differential DNAm analysis. Regional DNAm levels were calculated separately in each sex using the median β value across 42,776 reference autosomal CMRs for whole blood from the 450 K and EPIC arrays (excluding 881 reference CMRs where one or more probes did not pass quality control)^19^. Prior to differential DNAm analysis, a variability filter was applied to select reference CMRs with a range of >0.05 between the 10th and 90th percentiles of median β values for each CMR^103^, which yielded 29,363 variable reference CMRs in females, 29,190 variable reference CMRs in males, and 29,708 variable reference CMRs in the sex-combined sample (Supplementary Fig. 15). For comparison, CMRs were also called using the “cmr” function in CoMeBack, with cell-type-corrected β values as input, and employing the same parameters used to construct the reference (minimum Spearman correlation of 0.3, maximum distance 1 kb)^19^.

Propensity matching with the “full” method as implemented in the MatchIt package was used to calculate sample weights for regression, in order to improve covariate balance between PD cases and controls and to achieve more accurate effect estimations^112^. Weights were calculated separately in each sex based on smoking history, alcohol consumption, head trauma, and age with generalized linear regression and a probit link function, using a caliper width of 0.2 (Supplementary Fig. 16)^112^. We additionally adjusted for matching covariates in regression analysis, as this can reduce residual confounding and limit estimation bias^113^. Robust linear regression was performed with the model CMR median β ~ PD + age + smoking + alcohol consumption + head trauma + cell type PCs 1–6 + genotyping PCs 1–3 + plate + row, separately in each sex. Chip was not included, as TERRE subjects were not balanced by sex across chips, and some chips had few individuals from TERRE because TERRE and DIGPD samples were run together. CMRs were considered statistically significant at a minimum *p*_adj_ ≤ 0.05 and absolute median Δβ_adj_ ≥ 0.03 (β coefficient for PD from linear regression analysis adjusted for all above covariates). This effect size threshold was chosen to exceed the root mean square error (RMSE) between technical replicates (maximum 0.024 in TERRE).

Power calculations for sex-stratified EWAS were implemented using the pwrEWAS R package^51^. For females, we performed 1000 simulations with the following input parameters: 100 individuals (33% cases, 67% controls); target Δβ 0.03; 29,363 CpGs tested for differential methylation; 69 differentially methylated CpGs; detection β limit 0.03; “limma” method; FDR critical value 0.05; and adult blood tissue. For males, we performed 1000 simulations with the same parameters using 118 individuals (32% cases, 68% controls); 29,190 CpGs tested for differential methylation; and 2 differentially methylated CpGs. We also simulated the number of differentially methylated CMRs (*p*_adj_ ≤ 0.05, |median Δβ_adj_ | ≥0.03) in each sex under the null by shuffling PD case-control labels and repeating EWAS using the shuffled disease status, for a total of 1000 permutations. Adjusted *p*-values were calculated based on how many simulations had a larger or small number of differentially methylated CMRs than the real number under the null, divided by the number of permutations.

Propensity matching can improve effect estimation when comparing studies, and covariate adjustment following this matching reduces residual confounding and minimizes bias^113,114^. In order to assess the influence of demographic differences on replicability of PD-associated DNAm patterns across populations, we matched TERRE and the larger replication samples (PEG1, SGPD) on age, predicted smoking, and predicted neutrophil proportion prior to replication analysis, and adjusted all effect sizes for these factors (Fig. 1b, Supplementary Figs. 17, 18, Supplementary Methods)^19,115,116^. As DIGPD was of a similar size to TERRE (*n* = 222 after QC), this sample was not subset for replication analysis; however, effect sizes were adjusted for relevant covariates (Supplementary Methods).

The median CMR Δβ_adj_ was compared between TERRE and each replication sample for CMRs differentially methylated in TERRE at *p*_adj_ ≤ 0.05 (508 in females, 7 in males) and also covered in each data set, which varied according to array platform (EPIC versus 450 K) and probes removed during data quality control (506 female CMRs covered in DIGPD; 7 male CMRs covered in DIGPD; 155 female CMRs covered in PEG1 and SGPD; 4 male CMRs covered in PEG1 and SGPD). Pearson’s correlations were computed between TERRE case-control Δβ_adj_ values and DIGPD, PEG1, or SGPD case-control Δβ_adj_ values^52,53^. CMRs were considered replicable at median |Δβ_adj_| ≥0.03 in the same direction as in TERRE.

For each CMR median β found to be significantly associated with PD (*p*_adj_ ≤ 0.05, |Δβ_adj_| ≥0.03) in female subjects in TERRE, we performed sensitivity analysis to test the fit of a genotype (G) model, exposure (E) model, an additive genotype plus exposure (G + E) model, and a genotype-by-exposure (G × E) model (Fig. 3b), accounting for both PD status and the covariates included in our region-based EWAS. For G, we considered SNPs within a 75-kb window centered on each median CMR β associated with PD. For E, we considered pesticides with at least 10% of subjects exposed on average across imputations (3 in females; Supplementary Table 6, Supplementary Fig. 19); these included an overall exposure variable (never/gardening use) and exposure variables for occupational use of fungicides and insecticides (never/ever exposed). The E model results were combined across 10 imputations of pesticide exposure using the mice package, accounting for intersample differences by weighted regression (as described in EWAS section)^112,117^. Models were fit using “rlm” from the MASS package, Huber M-estimator, 150 iterations in R version 3.6.2^118^.

The full set of covariates for each model was as follows:

- G: CMR median β ~ **genotype** + PD + age + smoking + alcohol consumption + head trauma + cell type PCs 1–6 + genotype PCs 1–3 + plate + row, for all SNPs within 75 kb of each CMR
- E: CMR median β ~ **pesticide exposure status** + PD + age + smoking + alcohol consumption + head trauma + cell type PCs 1–6 + genotype PCs 1–3 + plate + row, for each pesticide
- G + E: CMR median β ~ **genotype** + **pesticide exposure status** + PD + age + smoking + alcohol consumption + head trauma + cell type PCs 1–6 + genotype PCs 1–3 + plate + row, for each pesticide and all SNPs within 75 kb of each CMR
- G × E: CMR median β ~ **genotype** + **pesticide exposure status** + **genotype:pesticide exposure status** + PD + age + smoking + alcohol consumption + head trauma + cell type PCs 1–6 + genotype PCs 1–3 + plate + row, for each pesticide and all SNPs within 75 kb of each CMR

In the first stage of our analysis, we computed the change in AIC of each model relative to a base model without G, E, or G × E terms, and assessed the significance of this change in model fit using the F-test, accounting for multiple testing within each pesticide exposure (Fig. 3b)^119^. Of the four tested, the model with the lowest AIC for each CpG was selected as best explaining DNAm^36,37,111^. In the second stage, we quantified the extent to which G, E, and G × E terms impacted the contribution of PD to changes in DNAm at each CMR. To do so, we fit the best model for each PD-associated CMR, and compared the relative change in effect of PD on DNAm. In addition, we examined whether genetic variants associated with DNAm at each CMR were within 1 Mb of any of those associated with PD status in a large independent GWAS^29^.

### Ethics and inclusion statement

This study was co-lead by researchers based in France, the country of recruitment for the TERRE and DIGPD samples. The French team was responsible for the study design, implementation, and molecular profiling of samples and was collaboratively involved in data analysis and manuscript writing, as agreed among study authors ahead of the research. The research protocol of the TERRE study was approved by the ethics committee of Hôpital du Kremlin-Bicêtre, and all subjects provided written informed consent. The research protocol of the DIGPD study was approved by the ethics committee of the University of Paris VI, and all subjects provided written informed consent. All data storage and analysis was conducted on French servers with the exception of some calculations using TERRE DNA methylation data, where a legal agreement was in place to transfer this data to Canada. The results are relevant for studying epigenetic patterns in the specific population of French agricultural workers focused on in this work, and similar epidemiological and genetic studies conducted in French PD patients, including the specific samples used in this work, are cited throughout.

### Reporting summary

Further information on research design is available in the Nature Research Reporting Summary linked to this article.

### Supplementary information

Supplementary File 1

Supplementary Table 8

Reporting Summary

## Data Availability

The TERRE and DIGPD DNAm and genotyping data analyzed in the present study are subject to access restrictions via the European Union General Data Protection Regulation (GDPR) and to maintain participant privacy. Requests for access can be directed to alexis.elbaz@inserm.fr, including the proposed purpose for data use, and are subject to governance constraints and privacy restrictions. The PEG1 and SGPD DNAm data analyzed in this study are available on GEO (GSE111629, GSE145361).

## Code availability

The code used in this manuscript is publicly available at github.com/samschaf/TERRE_GxE.
