# Supplementary material for: Genetic variation and pesticide exposure influence blood DNA methylation signatures in females with early-stage Parkinson’s disease
Source: NPJ Parkinsons Dis. 2024 May 7;10:98. doi: 10.1038/s41531-024-00704-3 (PMC11076573; doi:10.1038/s41531-024-00704-3)
Supplement: Supplementary file 1 [file 41531_2024_704_MOESM1_ESM.pdf]

DIGPD Study Group

Steering committee: Jean-Christophe Corvol, MD, PhD (Pitié-Salpêtrière Hospital, Paris, principal investigator of DIGPD), Alexis Elbaz, MD, PhD (CESP, Villejuif, member of the steering committee), Marie Vidailhet, MD (Pitié-Salpêtrière Hospital, Paris, member of the steering committee), Alexis Brice, MD (Pitié-Salpêtrière Hospital, Paris, member of the steering committee and PI for genetic analysis).
Statistical analyses: Alexis Elbaz, MD, PhD (CESP, Villejuif, PI for statistical analyses), Fanny Artaud, PhD (CESP, Villejuif, statistician).
Principal investigators for sites (alphabetical order): Frédéric Bourdain, MD (CH Foch, Suresnes, PI for site), Jean-Philippe Brandel, MD (Fondation Rothschild, Paris, PI for site), Jean-Christophe Corvol, MD, PhD (Pitié-Salpêtrière Hospital, Paris, PI for site), Pascal Derkinderen, MD, PhD (CHU Nantes, PI for site), Franck Durif, MD (CHU Clermont-Ferrand, PI for site), Richard Levy, MD, PhD (CHU Saint-Antoine, Paris, PI for site), Fernando Pico, MD (CH Versailles, PI for site), Olivier Rascol, MD (CHU Toulouse, PI for site).
Co-investigators (alphabetical order): Anne-Marie Bonnet, MD (Pitié-Salpêtrière Hospital, Paris, site investigator), Cecilia Bonnet, MD, PhD (Pitié-Salpêtrière Hospital, Paris, site investigator), Christine Brefel-Courbon, MD (CHU Toulouse, site investigator), Florence Cormier-Dequaire, MD (Pitié-Salpêtrière Hospital, Paris, site investigator), Bérangère Debilly, MD (CHU Clermont-Ferrand, site investigator), Bertrand Degos, MD, PhD (Pitié-Salpêtrière Hospital, site investigator), Alexis Elbaz, MD, PhD (Pitié-Salpêtrière Hospital, Paris, site investigator), Monique Galitsky (CHU de Toulouse, site investigator), David Grabli, MD, PhD (Pitié-Salpêtrière Hospital, Paris, site investigator), Andreas Hartmann, MD, PhD (Pitié-Salpêtrière Hospital, Paris, site investigator), Stephan Klebe, MD (Pitié-Salpêtrière Hospital, Paris, site investigator), Julia Kraemmer, MD (Pitié-Salpêtrière Hospital, site investigator), Lucette Lacomblez, MD (Pitié-Salpêtrière Hospital, Paris, site investigator), Sara Leder, MD (Pitié-Salpêtrière Hospital, Paris, site investigator), Graziella Mangone, MD, PhD (Pitié-Salpêtrière Hospital, Paris, site investigator), Louise-Laure Mariani, MD (Pitié-Salpêtrière Hospital, Paris, site investigator), Ana-Raquel Marques, MD (CHU Clermont Ferrand, site investigator), Valérie Mesnage, MD (CHU Saint Antoine, Paris, site investigator), Julia Muellner, MD (Pitié-Salpêtrière Hospital, Paris, site investigator), Fabienne Ory-Magne, MD (CHU Toulouse, site investigator), Violaine Planté-Bordeneuve, MD (Henri Mondor Hospital, Créteil, site investigator), Emmanuel Roze, MD, PhD (Pitié-Salpêtrière Hospital, Paris, site investigator), Melissa Tir, MD (CH Versailles, site investigator), Marie Vidailhet, MD (Pitié-Salpêtrière Hospital, Paris, site investigator), Hana You, MD (Pitié-Salpêtrière Hospital, Paris, site investigator).

Neuropsychologists: Eve Benchetrit, MS (Pitié-Salpêtrière Hospital, Paris, neuropsychologist), Julie Socha, MS (Pitié-Salpêtrière Hospital, Paris, neuropsychologist), Fanny Pineau, MS (Pitié-Salpêtrière Hospital, Paris, neuropsychologist), Tiphaine Vidal, MS (CHU Clermont-Ferrand,

neuropsychologist), Elsa Pomies (CHU de Toulouse, neuropsychologist), Virginie Bayet (CHU de Toulouse, neuropsychologist).
Genetics core: Alexis Brice (Pitié-Salpêtrière Hospital, Paris, PI for genetic studies), Suzanne Lesage, PhD (INSERM, ICM, Paris, genetic analyses), Khadija Tahiri, PhD (INSERM, ICM, Paris, lab technician) Hélène Bertrand, MS (INSERM, ICM, Paris, lab technician), Graziella Mangone, MD, PhD (Pitié-Salpêtrière Hospital, Paris, genetic analyses).
Sponsor activities and clinical research assistants: Alain Mallet, PhD (Pitié-Salpêtrière Hospital, Paris, sponsor representative), Coralie Villeret (Hôpital Saint Louis, Paris, Project manager), Merry Mazmanian (Pitié-Salpêtrière Hospital, Paris, project manager), Hakima Manseur (Pitié-Salpêtrière Hospital, Paris, clinical research assistant), Mostafa Hajji (Pitié-Salpêtrière Hospital, Paris, data manager), Benjamin Le Toullec, MS (Pitié-Salpêtrière Hospital, Paris, clinical research assistant), Vanessa Brochard, PhD (Pitié-Salpêtrière Hospital, Paris, project manager), Monica Roy, MS (CHU de Nantes, clinical research assistant), Isabelle Rieu, PhD (CHU Clermont-Ferrand, clinical research assistant), Stéphane Bernard (CHU Clermont-Ferrand, clinical research assistant), Antoine Faurie-Grepon (CHU de Toulouse, clinical research assistant).

Supplementary Methods

Study inclusion and exclusion criteria

For the TERRE study, individuals aged 18-75 who applied for healthcare through the Mutualité Sociale Agricole and who were not receiving free health care for dementia were eligible (1). PD patients were defined as having parkinsonism (≥ 2 of rest tremor, bradykinesia, rigidity, impaired postural reflexes) with exclusion of more prominent nervous system involvement and of drug-induced parkinsonism (2). Controls were randomly selected from individuals affiliated with the Mutualité Sociale Agricole and not receiving healthcare for PD or dementia.

For the DIGPD study, patients were recruited between May 2009 and July 2013 at 8 French hospitals, and were eligible if diagnosed with PD according to the UK Brain Bank criteria, and if disease duration was 5 years or less at time of recruitment (3,4). Controls were selected from friends and relatives of PD patients, and were required to be free of PD according to the UK Brain Bank criteria (4).

Individuals recruited for the PEG1 study were residents of Fresno, Kern, or Tulare Counties in California, USA, and had lived in California for ≥ 5 years (5). PD patients were included if they were diagnosed within 3 years of recruitment, and satisfied the UK Brain Bank and Gelb criteria (4,6). Controls free of PD and aged 65 and older were recruited from Medicare lists, and additional controls free of PD and aged 35 and older were recruited from randomly selected tax assessor residential units in each county. One control per household was eligible for the study (7).

The SGPD study included individuals from the QPP, NZBRI, and SYD cohorts (8). For the QPP cohort, PD patients were included if they satisfied the Calne diagnostic criteria, and controls were either spouses of patients, siblings of patients, or community volunteers from the same area as patients, with the same ethnicity, and of similar age (8,9). PD patients from the NZBRI cohort were recruited by the NZBRI, and were excluded if they had a history of learning disability, severe head injury, stroke, or other neurological impairment, or major psychiatric complications. Finally, patients and controls from the SYD cohort were recruited at the Parkinson's Disease Research Clinic, Brain and Mind Research Institute at the University of Sydney, with PD diagnosed according to the UK Brain Bank criteria (4).

Genotyping data generation and preprocessing

The Illumina NeuroChip array was used to generate genotype data for cases and controls from TERRE starting at 486,137 SNPs. Genotype data were processed using the RICOPILI pipeline (10). Preimputation quality control (QC) first consisted of a set of cleaning filters applied using PLINK 1.9 (11): removal of strand-ambiguous SNPs, mismatch between recorded sex and sex determined on the array, a 0.05 call rate filter for SNPs, an individual missingness filter of 0.02, a minor allele frequency (MAF) filter of 0.01, and finally a heterozygosity filter removing individuals with F > 0.2. Variants on sex chromosomes and variants with Hardy–Weinberg equilibrium $p < 1 \times 10^{-10}$ were then removed.

To estimate genotype PCs, we used a set of SNPs pruned in the following manner. Samples were processed to remove SNPs in linkage by two rounds of pruning of SNPs within 200 kb and in linkage disequilibrium (squared Pearson's correlation between SNPs [r^2] > 0.2) in a pairwise manner (i.e., --indep-pairwise 200 0.2 in PLINK 1.9 was run twice). We next removed SNPs with MAF < 0.05, and SNPs in highly recombinant regions. The samples were then filtered to ensure that identity by descent did not exceed $|F_{ST}| > 0.2$ between samples within each ancestry group. Ten genotype PCs were computed using FastPCA (Supplementary Fig. 9) (12).

SNPs were then aligned to 1000 Genomes Phase 3 using BCFtools (13), and imputed to the 1000 Genomes Phase 3 European reference panel using the Michigan Imputation Server. Variants were filtered to have an imputation quality $R2$ (squared Pearson's correlation between imputed and true genotypes for a given SNP) > 0.3 and MAF within 0.1 of the 1000 Genomes Phase 3 European population. Finally, imputed SNPs associated with their genotype batch were removed, and inflation of the association between genotype and PD was checked by logistic regression accounting for sex, age at collection, and three genotyping PCs (with genomic inflation factor, $\lambda_{GC} = 1.06$ in TERRE) (Supplementary Fig. 10), yielding 8,354,189 SNPs for analysis.

Propensity matching and effect size adjustment in PEG1, DIGPD, and SGPD (replication samples)

For the PEG1 sample (n = 539 after QC), two sets of matching were performed with the MatchIt package, using the optimal method with glm distance estimation, a probit link function, and caliper width of 0.2: 1) between female (or male) PD cases and controls, and 2) across females (or males) from both samples (14,15). For both sets, individuals were matched on age, predicted neutrophil proportion, smoking score, and ethnicity (retaining subjects of European ancestry in each sample; Supplementary Figs. 17,18). This resulted in 100 females from PEG1 (45 cases, 55 controls) matched to 100 females from TERRE (33 cases, 67 controls), and 118 males from PEG1 (59 cases, 59 controls) matched to 118 males from TERRE (38 cases, 80 controls). CMR median β values were estimated separately in PEG1 females and males based on these subsets of individuals, and PD case-control CMR $\Delta\beta_{adj}$ values were calculated by regressing each CMR median β on disease status, age, ethnicity, smoking score, and three cell type PCs (explaining 86% of variance in cell type composition; using "rlm" from the MASS package, Huber M-estimator, 500 iterations) (16).

For SGPD (n = 1751 after QC), two sets of matching were performed as stated above, excluding ethnicity as all individuals were of European descent (Supplementary Figs. 17,18). When matching between SGPD and TERRE, a 4:1 ratio was used, in order to take advantage of the large sample size in SGPD. The matched datasets consisted of 400 females from SGPD (202 cases, 198 controls) matched to 100 females from TERRE, and 472 males from SGPD (241 cases, 231 controls) matched to 118 males from TERRE. The model median CMR β ~ disease status + age + smoking score + cell type PCs 1-3 (explaining 83% of variance in cell type composition) was used to calculate $\Delta\beta_{adj}$ values, separately in each sex.

Finally, as DIGPD was of a similar size to TERRE (n = 222 after QC), this sample was not subset for replication analysis. In both sexes, chip was confounded with age, and was thus not included in linear regression models, and in males, row was also excluded due to a mild association with disease status (r = 0.16, p = 0.07). Thus, the model median CMR β ~ disease status + age + smoking score + cell type PCs 1-4 (explaining 89% of variance in cell type composition) + genotype PCs 1-3 + plate + row was used to calculate $\Delta\beta_{adj}$ values in females, and the same model without row was used in males.

Sensitivity analysis for levodopa daily dosage and time since pesticide exposure in TERRE

22 of 23 female PD patients receiving levodopa had recorded information on levodopa equivalent daily dosage (LED). The following model was applied to CMR median beta values from the 22 patients: CMR median β ~ LED + age + plate + cell type PC 1. Additional covariates used in EWAS were excluded due to high model variance inflation factors when used in the subset of 22 patients. The variance inflation factors in the reduced model were 1.16 (LED), 1.48 (age), 1.56 (plate), and 1.13 (cell type PC 1), respectively.

Additionally, the lag time between last known pesticide exposure and DNAm measurement in each subject was recorded. In this sensitivity analysis, we assessed genetic and exposure related influences on CMRs across all females using the same linear model described in Methods, coding each exposure variable based on this lag time. Specifically, we constructed a variable for recent exposure as reported exposure at less than the median lag time across samples, and a variable for past exposure as a reported exposure at greater than or equal to the median lag time across samples. We then assessed E, G+E, and G×E models including both of these variables, accounting for each covariate present in our original analysis (See Methods).

Supplementary Figures

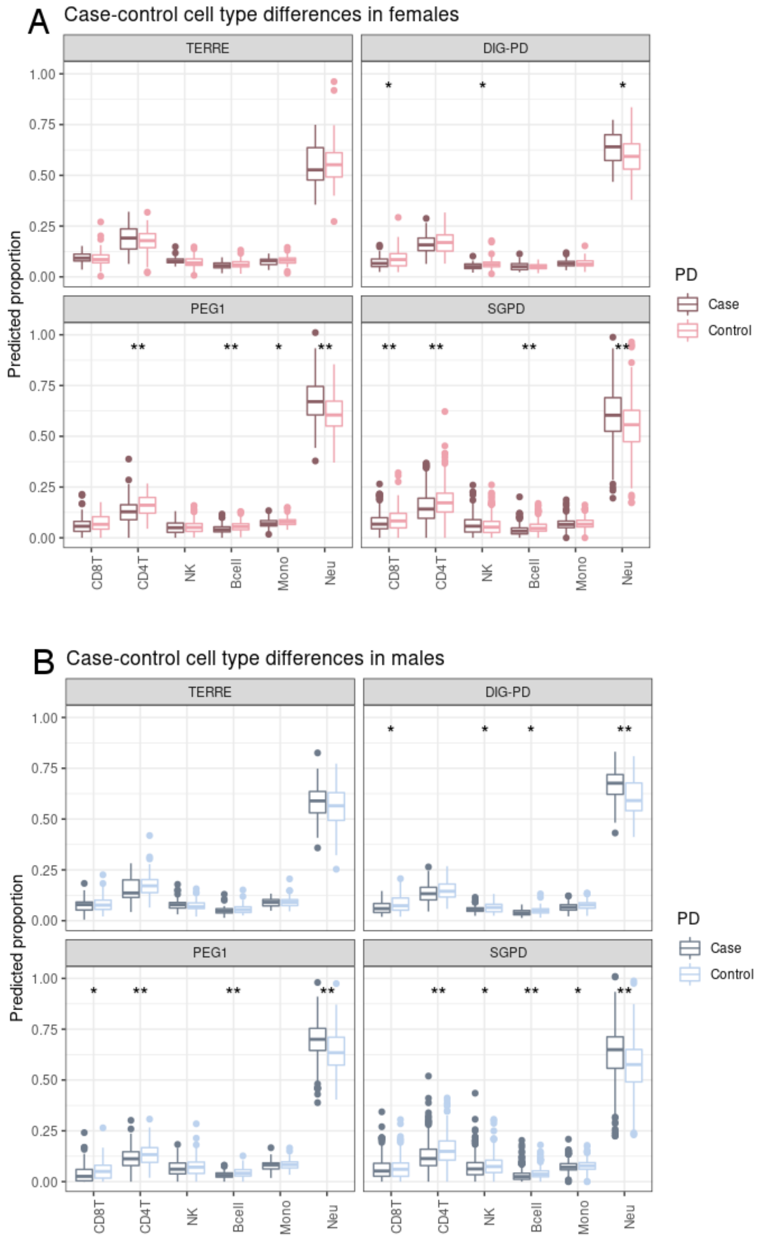

Supplementary Figure 1. Predicted immune cell composition in PD cases and controls from TERRE, DIGPD, and SGPD, stratified by sex. For comparison across samples, pictured cell type proportions in TERRE are predicted using the 6-cell type IDOL library (17).* $p_{adj} < 0.05$, ** $p_{adj} < 0.01$, *** $p_{adj} < 0.001$ (*t* test with Benjamini–Hochberg adjustment). (A) Predicted cell type proportions in females from TERRE, DIGPD, PEG1, and SGPD. Dark pink: PD cases; bright pink: controls. (B) Predicted cell type proportions in males from TERRE, DIGPD, PEG1, and SGPD. Dark blue: PD cases; light blue: controls.

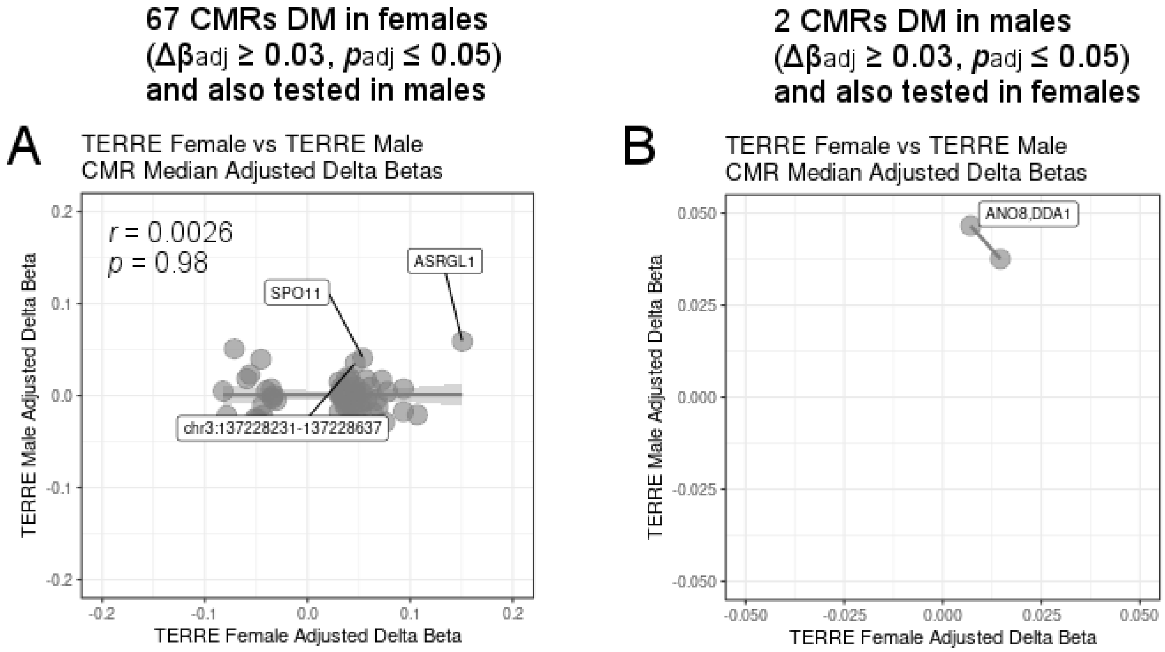

Supplementary Figure 2. Correlation of PD-associated blood DNA methylation patterns in females and males from TERRE. CMRs that passed median $|\Delta\beta_{adj}| \geq 0.03$ and $p_{\text{adj}} \leq 0.05$ in TERRE epigenome-wide association analyses in each sex are shown. x-axis: median CMR $|\Delta\beta_{adj}|$ in females from TERRE (n = 100); y-axis: median CMR $|\Delta\beta_{adj}|$ in males from TERRE (n = 118). (A) $\Delta\beta_{adj}$ correlations for 67 CMRs differentially methylated in females and also tested for differential DNAm in males. Pearson's correlation coefficient (r) and correlation p-value are shown. Labeled CMRs had median $|\Delta\beta_{adj}| \geq 0.03$ in the same direction in both sexes. (B) $\Delta\beta_{adj}$ correlations for 2 CMRs differentially methylated in females and also tested for differential DNAm in males. DM: differentially methylated.

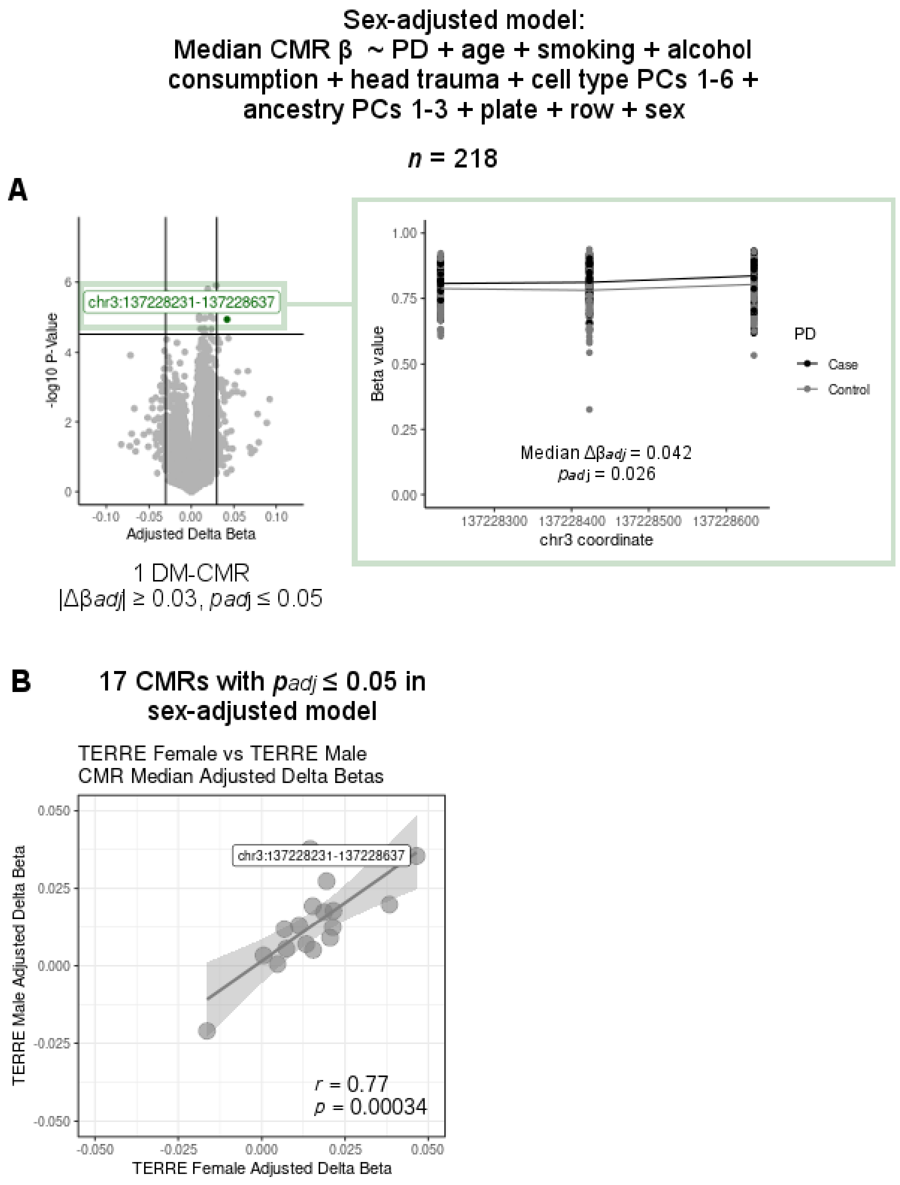

Supplementary Figure 3. PD-associated differentially methylated CMR identified with sex-adjusted model. (A) Volcano plot: Adjusted PD case-control DNAm differences in TERRE (sex-combined sample, *n* = 218). Colored point passed thresholds of median CMR $|\Delta\beta_{adj}| \geq 0.03$ and $p_{adj} \leq 0.05$. Inset: the chr3:137228231-137228637 CMR is shown. y-axis: β value (level of DNAm) in subjects from TERRE. Black: PD cases; grey: controls. (B) $\Delta\beta_{adj}$ correlations, Pearson's correlation coefficient (*r*), and correlation *p*-value for 17 CMRs that passed $p_{adj} \leq 0.05$ in the sex-adjusted analysis are shown. x-axis: median CMR $|\Delta\beta_{adj}|$ in females from TERRE (*n* = 100); y-axis: median CMR $|\Delta\beta_{adj}|$ in males from TERRE (*n* = 118). DM: differentially methylated.

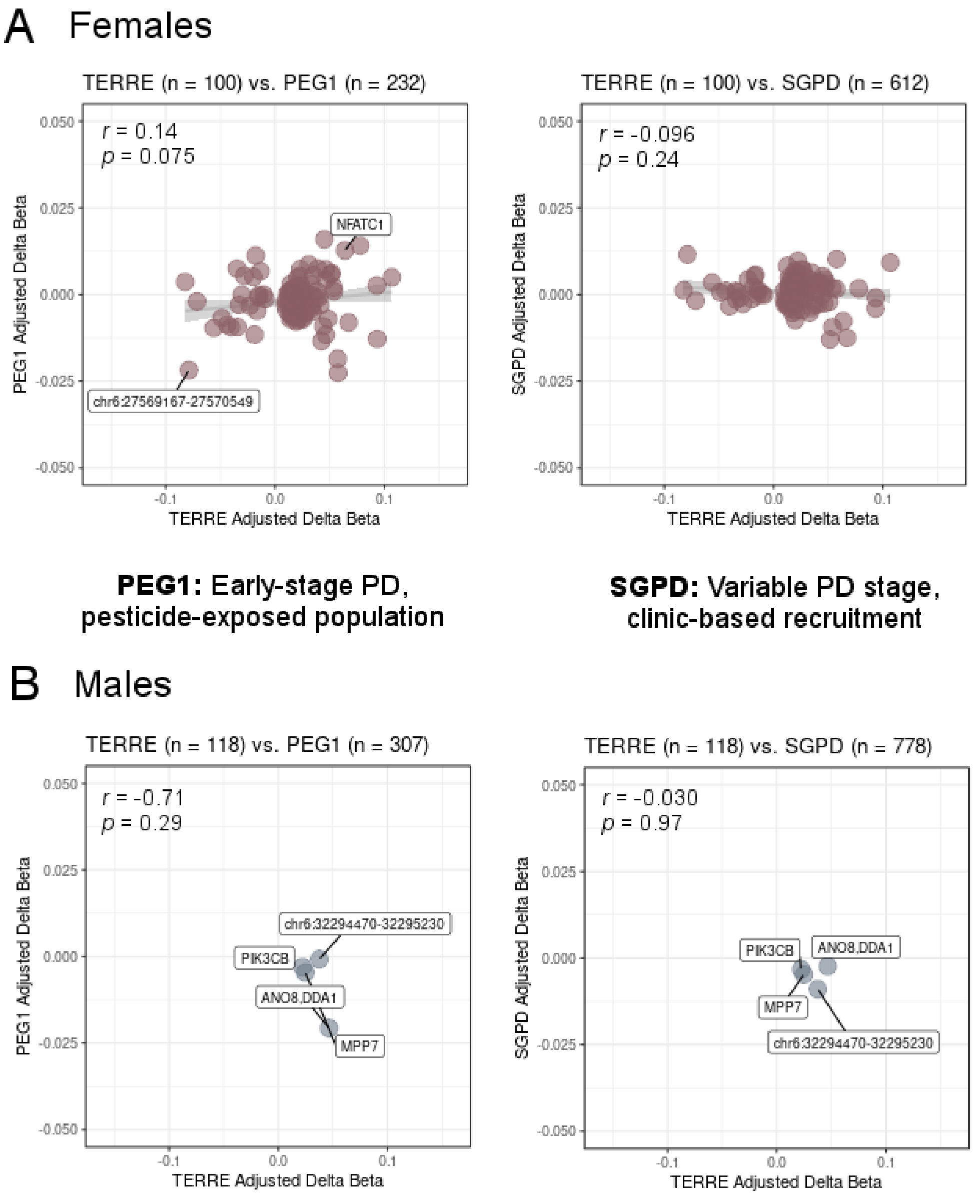

Supplementary Figure 4. Correlation of PD-associated blood DNA methylation patterns in TERRE and other populations before propensity matching. CMRs that passed $p_{\text{adj}} \leq 0.05$ in TERRE epigenome-wide association analyses in each sex are shown (508 total in females: 155/508 covered in PEG1 and SGPD 450K array datasets, 7 in males: 4/7 covered in PEG1 and SGPD). x-axis: median CMR $|\Delta\beta_{adj}|$ in individuals from TERRE; y-axis: median CMR $|\Delta\beta_{adj}|$ in individuals from PEG1 or SGPD (individuals with complete age data). Pearson's correlation coefficients (*r*) and *p*-values are shown. (A) Left to right: $\Delta\beta_{adj}$ correlations in females from PEG1 and SGPD. (B) Left to right: $\Delta\beta_{adj}$ correlations in males from PEG1 and SGPD.

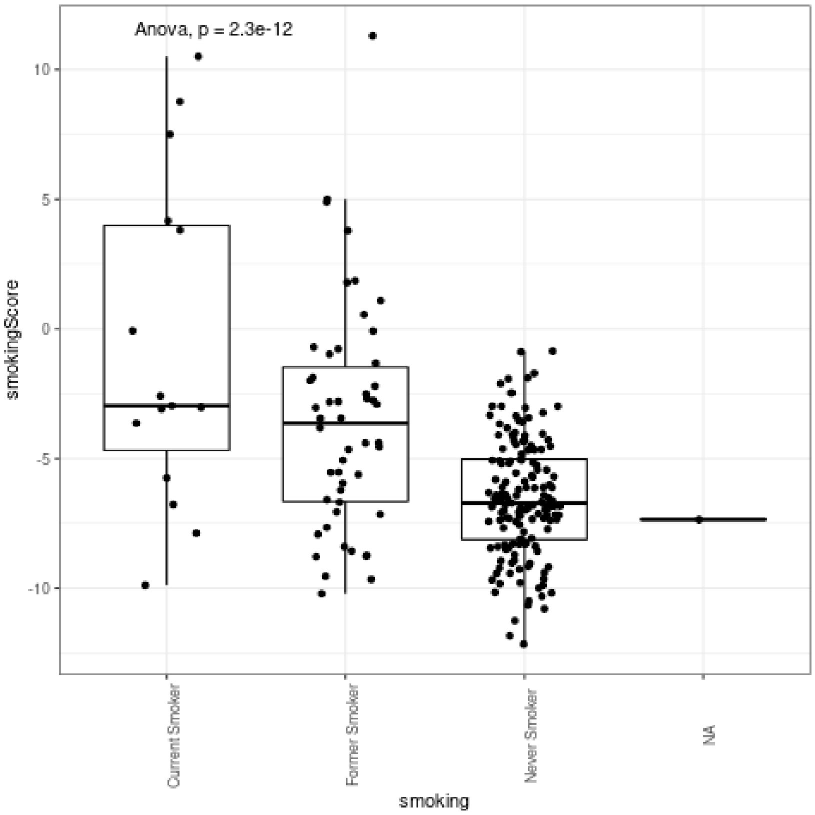

Supplementary Figure 5. Self-reported current and former smokers had higher median smoking scores than never-smokers in TERRE. x-axis: self-reported smoking status; y-axis: smoking score, predicted by the "SSc" method in the EpiSmokEr R package (18). NA: smoking information unavailable. *n* = 119.

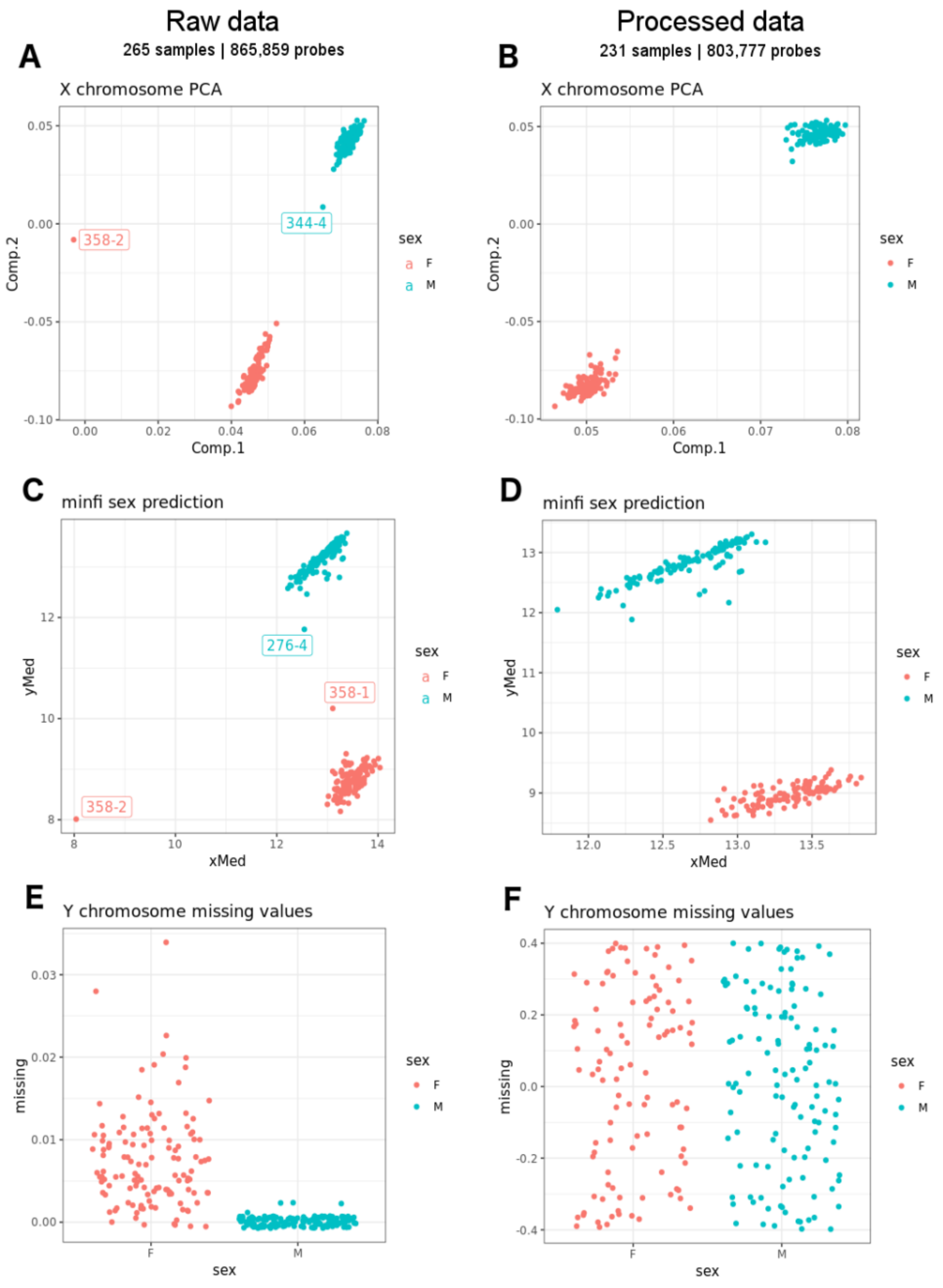

Supplementary Figure 6. Confirmation of biological sex in TERRE. Raw methylation data are shown on the left; processed methylation data (following outlier removal, normalization, and probe filtering) are shown on the right. Red: individuals self-reported as female; blue: individuals self-reported as male. (A, B) PCA of X chromosome β values. (C, D) X and Y chromosome intensities as calculated using *getSex* from the minfi R package. (E, F) Proportion of missing β values on the Y chromosome (expected to be near zero for males and above zero for females before data processing, indicating detection of the Y chromosome in males only).

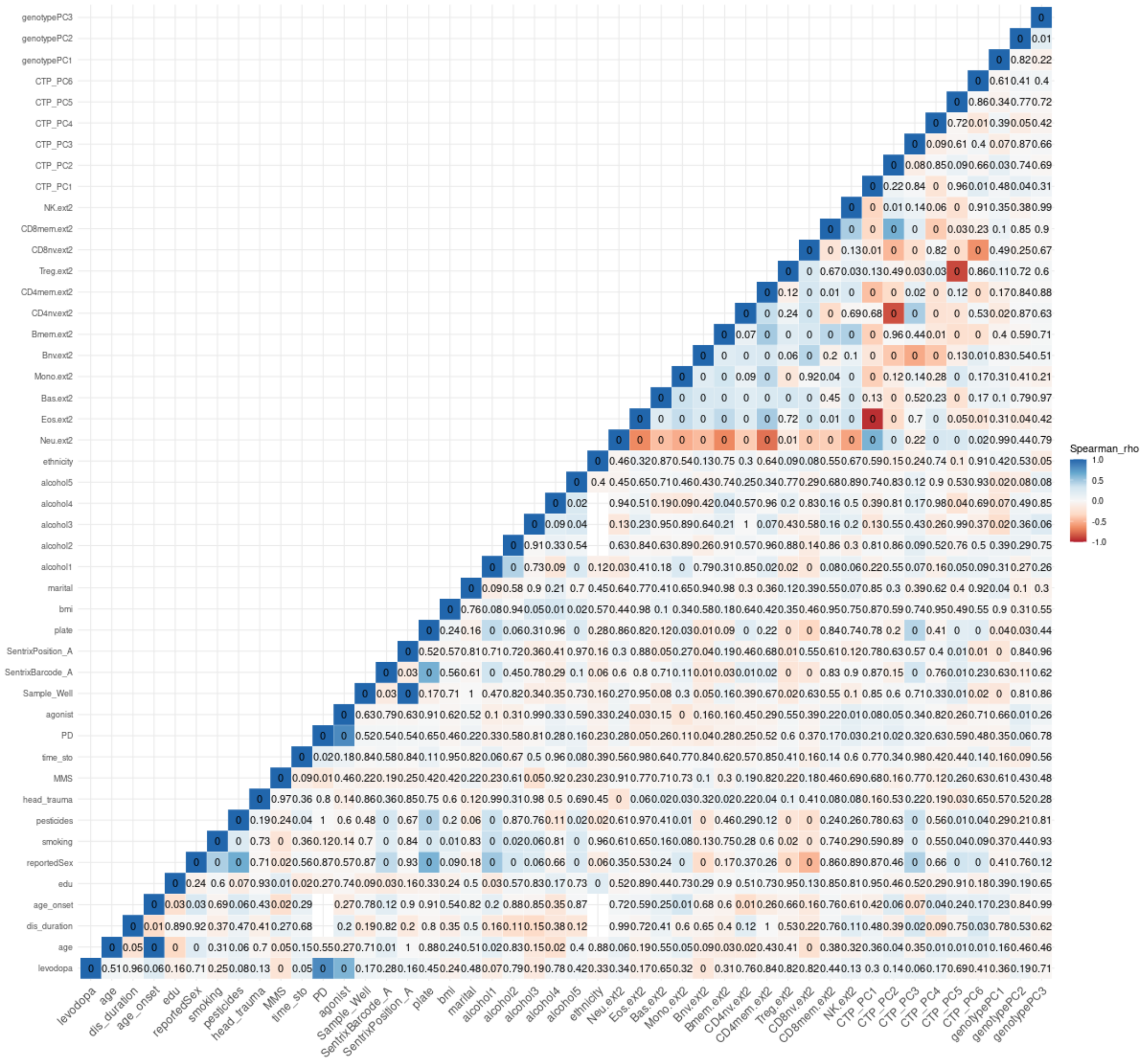

Supplementary Figure 7. Correlation between demographic variables, predicted cell type proportions, genotype PCs, and batch variables in TERRE. $n = 218$. Cell fill color: Spearman's ρ. Cell text: Spearman correlation p-value ("0": $p < 0.01$).

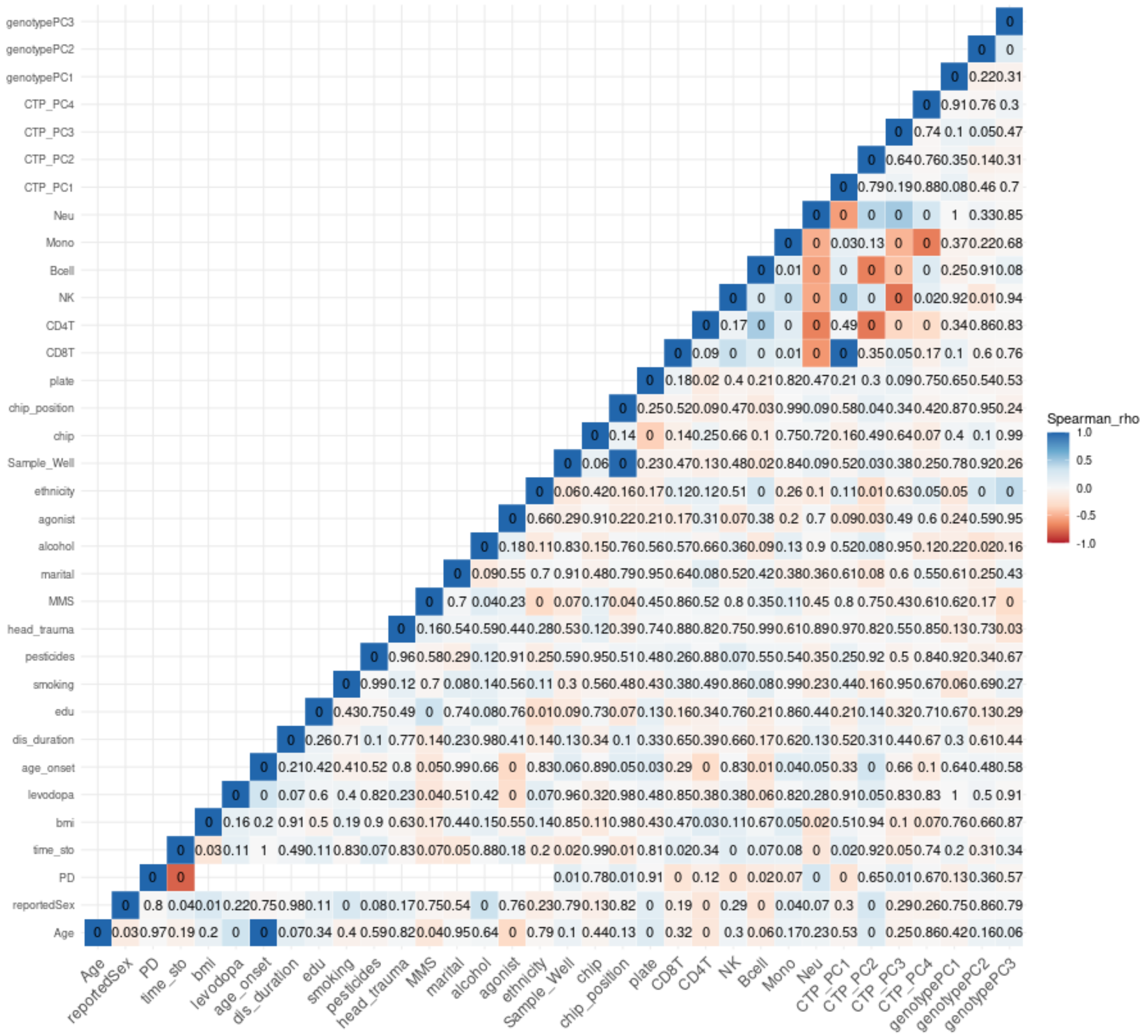

Supplementary Figure 8. **Correlation between demographic variables, predicted cell type proportions, genotype PCs, and batch variables in DIGPD**. $n = 222$. Cell fill color: Spearman's ρ. Cell text: Spearman correlation p-value ("0": $p < 0.01$).

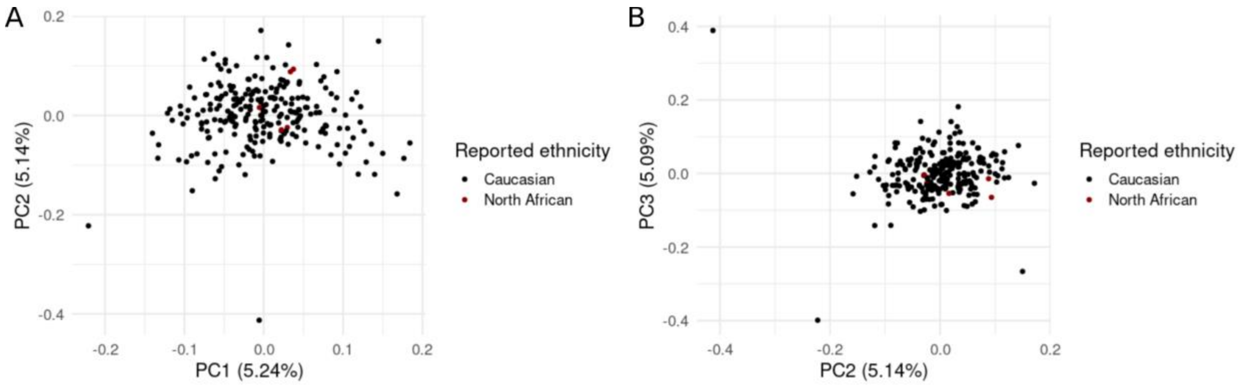

Supplementary Figure 9. TERRE participants plotted on their first three genotype PCs. Genotype PCs were computed prior to imputation after linkage disequilibrium (LD) pruning. (A) PC1 and PC2. (B) PC2 and PC3.

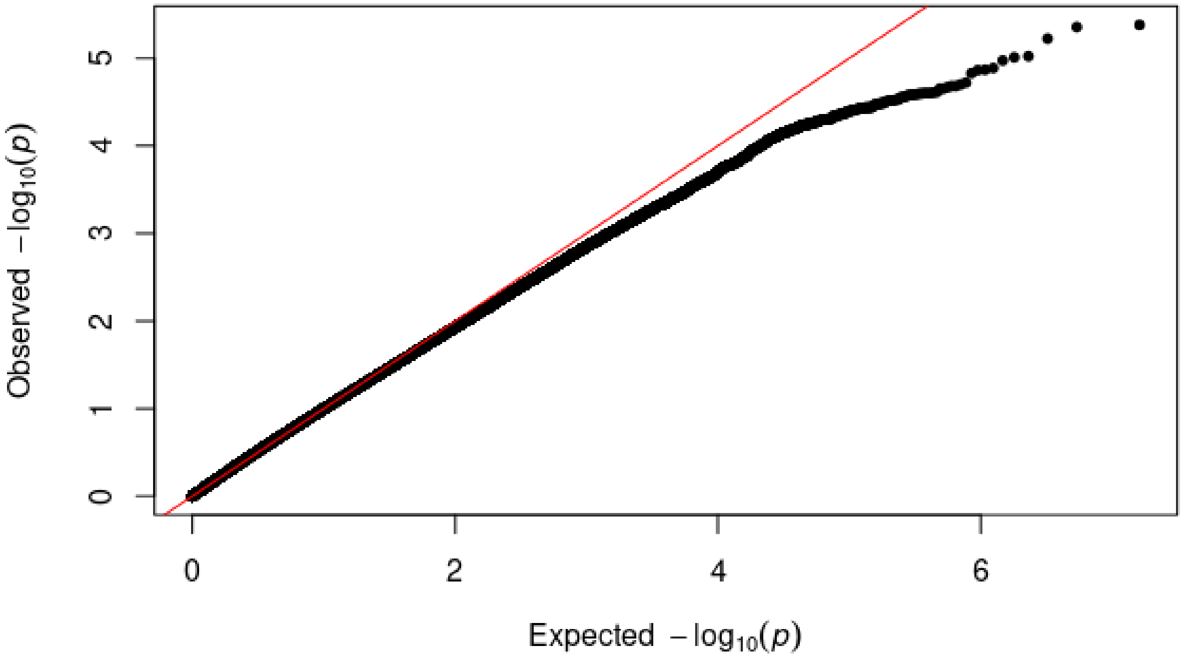

Supplementary Figure 10. TERRE Q-Q for PD GWAS. Q-Q Plot for association between imputed genotypes in TERRE and PD status, accounting for sex, age at collection, and three genotype PCs.

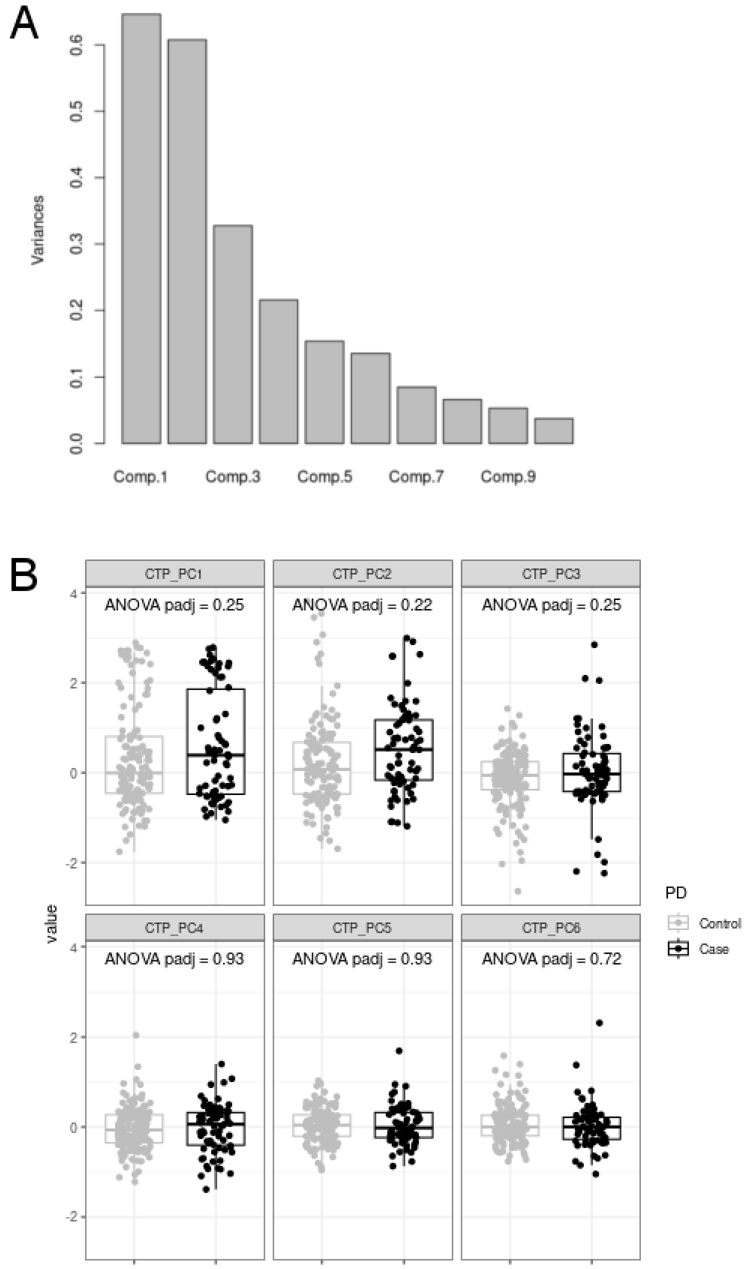

Supplementary Figure 11. Robust principal components of cell type composition in TERRE and their correlation with disease status. (A) The first 10 PCs representing the variance in cell type composition among the 218 TERRE participants included in this study. x-axis: principal component of cell type; y-axis: proportion of variance in cell type composition explained. (B) Case-control differences in loadings for cell type PCs 1-6, explaining 89% of variance in cell type composition in TERRE. x-axis: PD status; y-axis: PCA loadings. *p*-values were adjusted for multiple comparisons using the Benjamini-Hochberg method.

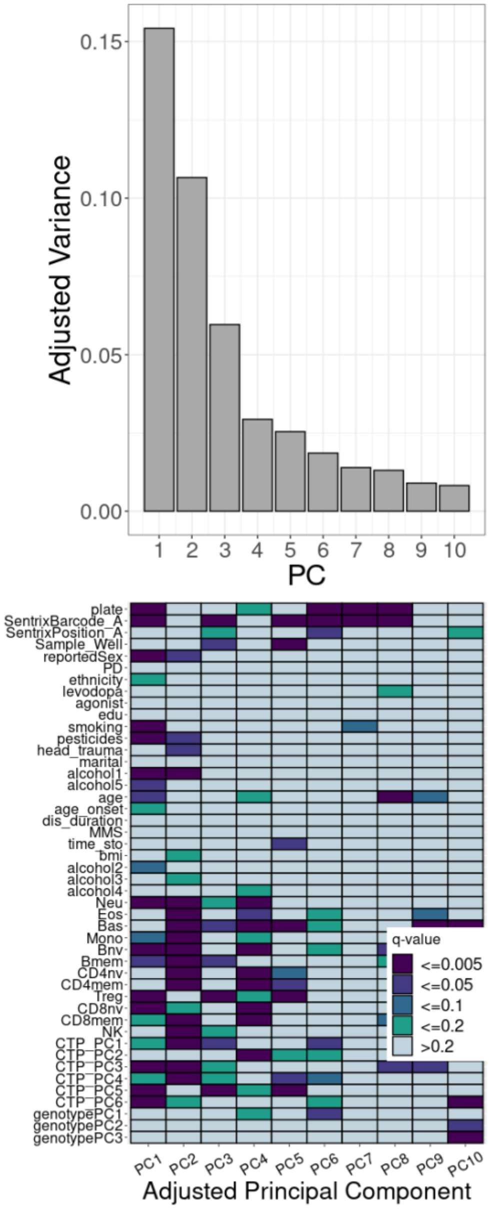

Supplementary Figure 12. Correlation between principal components (PCs) of normalized and probe-filtered β values in TERRE, demographic data, technical variables, predicted cell type proportions, and genotype PCs. PCA on β values for 803,777 EPIC probes and 219 subjects from TERRE passing quality control is shown. 1 subject was later removed due to missing self-reported smoking status. SentrixBarcode_A: sample chip; SentrixPosition_A: position on chip; PD: Parkinson's disease status; alcohol1: alcohol consumption; alcohol2: weekly wine consumption; alcohol3: weekly beer consumption; alcohol4: weekly aperitif consumption; alcohol5: change in alcohol consumption over time; MMS: Mini-Mental State Examination score; time_sto: sample storage time; bmi: body mass index; CTP: cell type proportion; PC: principal component.

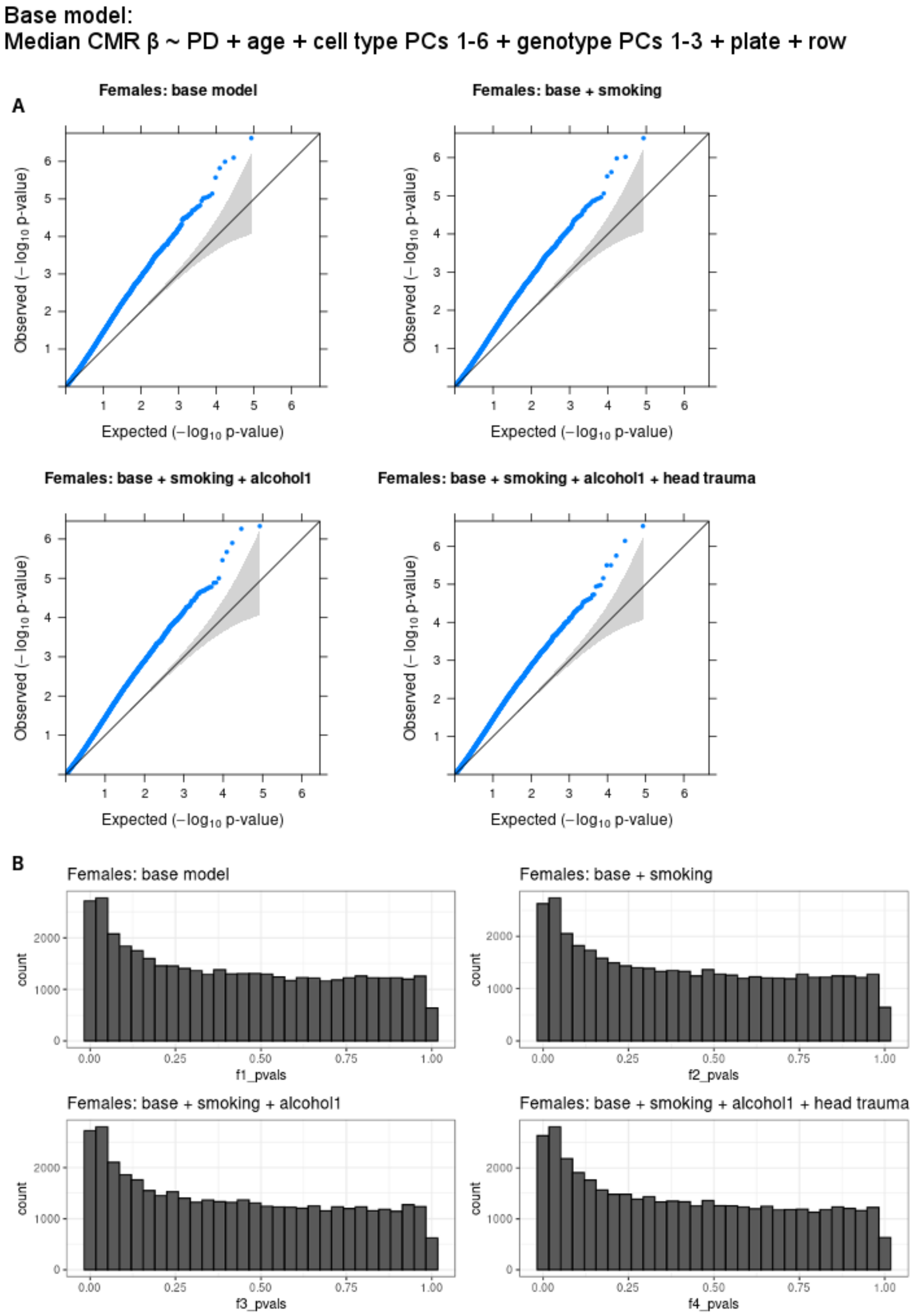

Supplementary Figure 13. Inclusion of smoking, alcohol consumption, and head trauma as covariates had little impact on model diagnostics in females from TERRE. (A) Q-Q plots of $-\log_{10}$ observed vs. expected *p*-values for PD coefficient of each model. (B) *p*-value histograms for PD coefficient of each model. Alcohol1: alcohol consumption.

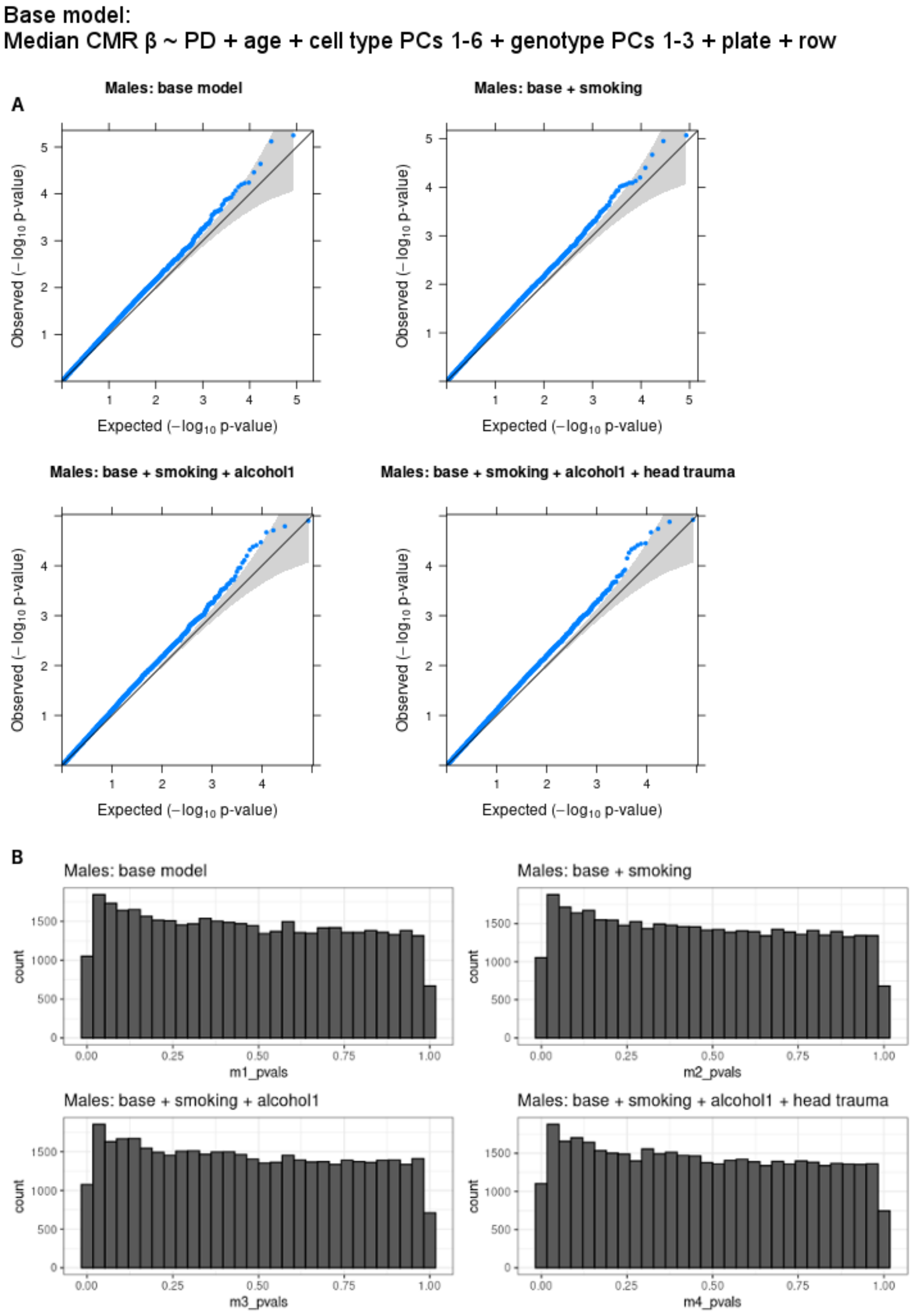

Supplementary Figure 14. Inclusion of smoking, alcohol consumption, and head trauma as covariates had little impact on model diagnostics in males from TERRE. (A) Q-Q plots of –$\log_{10}$ observed vs. expected *p*-values for PD coefficient of each model. (B) *p*-value histograms for PD coefficient of each model. Alcohol1: alcohol consumption.

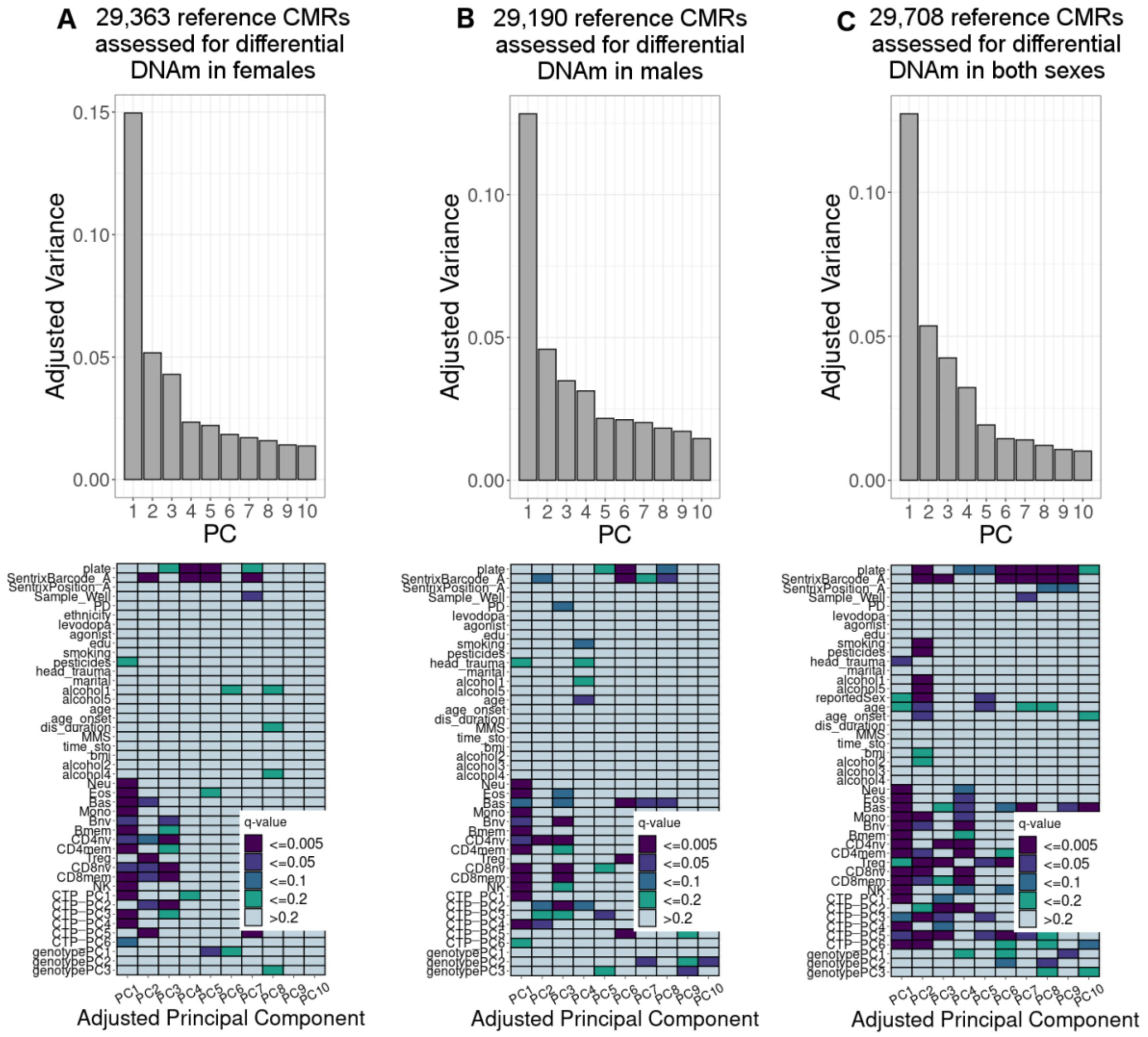

Supplementary Figure 15. Correlation between principal components (PCs) of median reference CMR β values in TERRE, demographic data, technical variables, predicted cell type proportions, and genotype PCs for CMRs variable in (A) only females, (B) only males, or (C) the sex-combined sample. SentrixBarcode_A: sample chip; SentrixPosition_A: position on chip; PD: Parkinson's disease status; alcohol1: alcohol consumption; alcohol2: weekly wine consumption; alcohol3: weekly beer consumption; alcohol4: weekly aperitif consumption; alcohol5: change in alcohol consumption over time; MMS: Mini-Mental State Examination score; time_sto: sample storage time; bmi: body mass index; CTP: cell type proportion; PC: principal component.

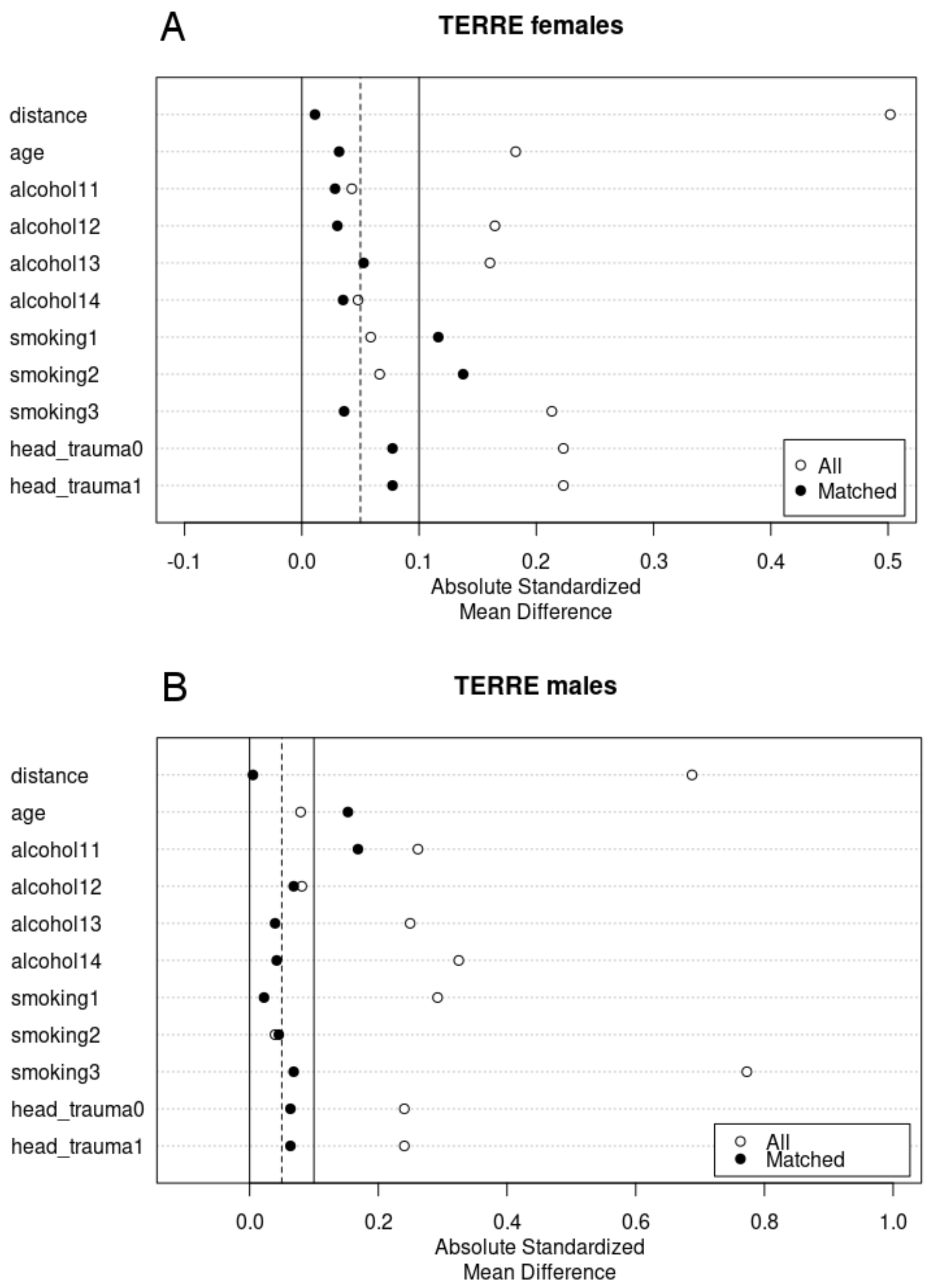

Supplementary Figure 16. Propensity matching in TERRE. Absolute standardized mean differences in age, alcohol consumption (“alcohol1”; 1: never; 2: occasionally; 3: regularly; 4: daily), smoking (1: never smoker; 2: former smoker; 3: current smoker), and history of head trauma (0: no; 1: yes) before and after matching are shown for: (A) TERRE female cases (*n* = 33) and controls (*n* = 67), and (B) TERRE male cases (*n* = 38) and controls (*n* = 80). In both instances, “full” matching was performed, using “glm” distance measure and a probit link function.

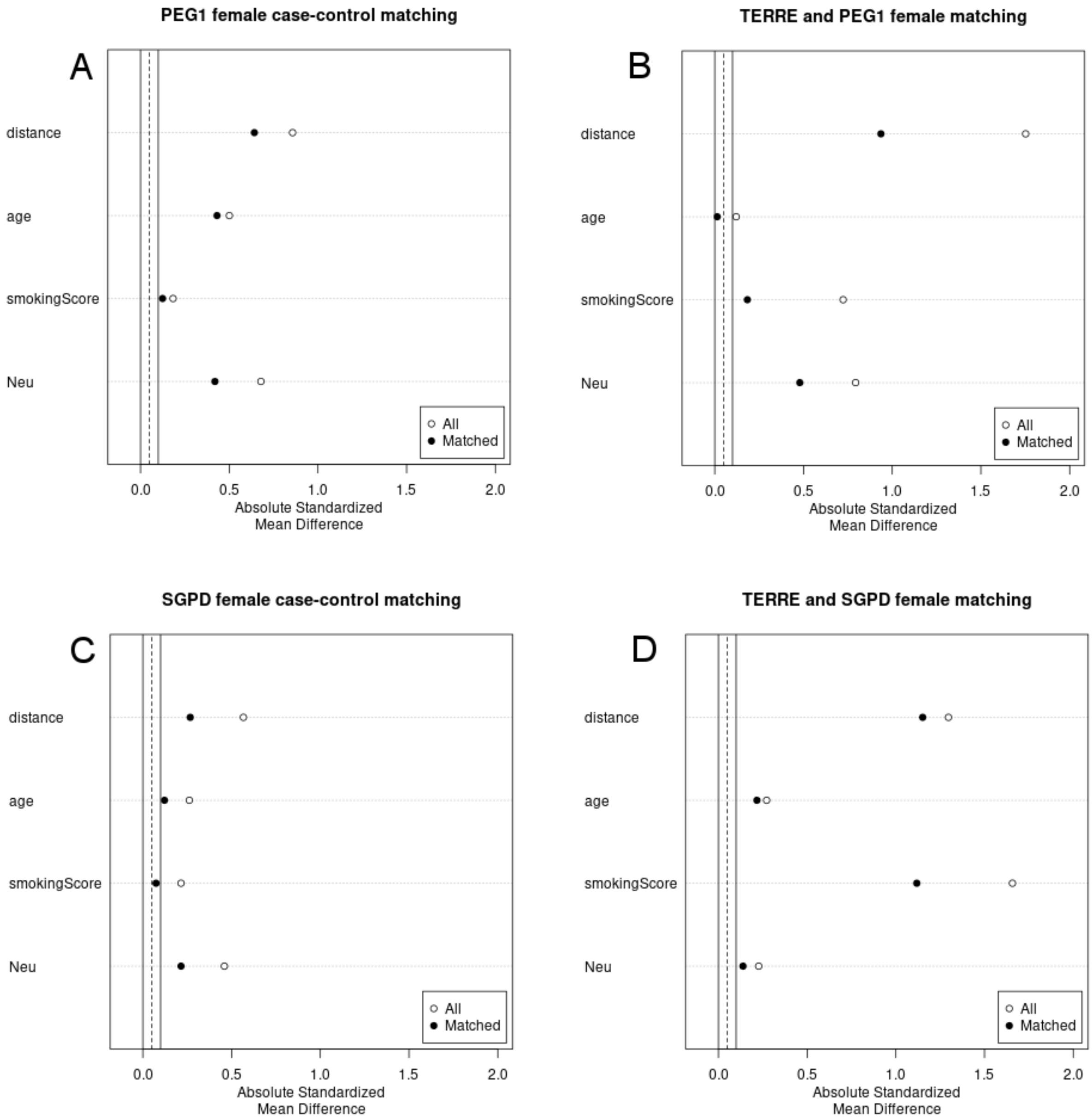

Supplementary Figure 17. Propensity matching in females from PEG1 and SGPD. Absolute standardized mean differences in age, predicted smoking score (5) and predicted neutrophil proportion (6) before and after matching are shown for: (A) PEG1 female PD cases (125 before matching, 107 after matching) and controls (107); (B) TERRE (100) and PEG1 (214 before matching, 100 after matching) females; (C) SGPD female PD cases (265) and controls (347 before matching, 265 after matching); (D) TERRE (100) and SGPD (530 before matching, 400 after matching) females. In all instances, “optimal” matching was performed, using “glm” distance measure and a probit link function. TERRE and SGPD females were matched in a 4:1 ratio.

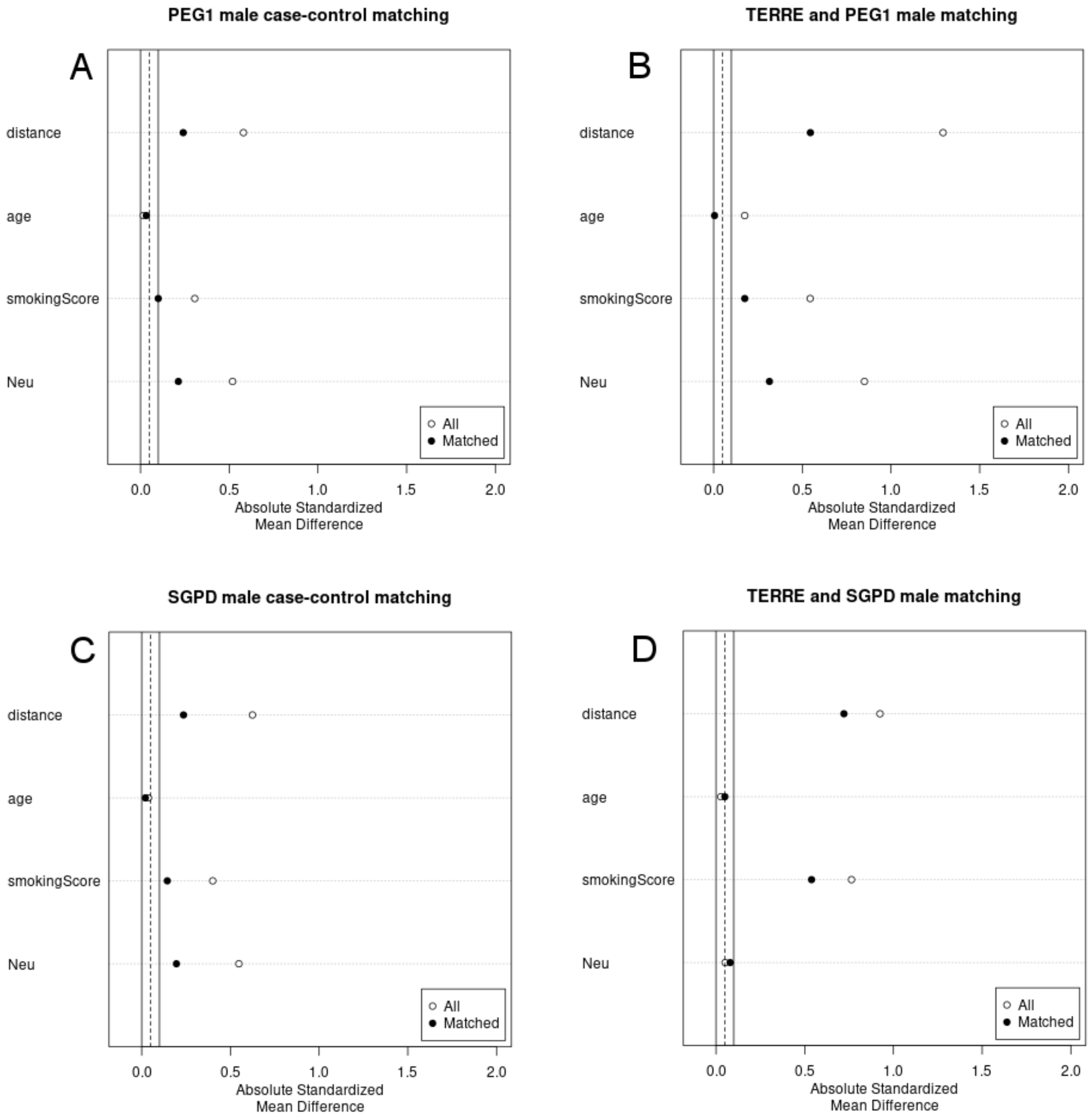

Supplementary Figure 18. Propensity matching in males from PEG1 and SGPD. Absolute standardized mean differences in age, predicted smoking score (5) and predicted neutrophil proportion (6) before and after matching are shown for: (A) PEG1 male PD cases (187 before matching, 120 after matching) and controls (120); (B) TERRE (118) and PEG1 (240 before matching, 118 after matching) males; (C) SGPD male PD cases (467 before matching, 311 after matching) and controls (311); (D) TERRE (118) and SGPD (622 before matching, 472 after matching) males. In all instances, “optimal” matching was performed, using “glm” distance measure and a probit link function. TERRE and SGPD males were matched in a 4:1 ratio.

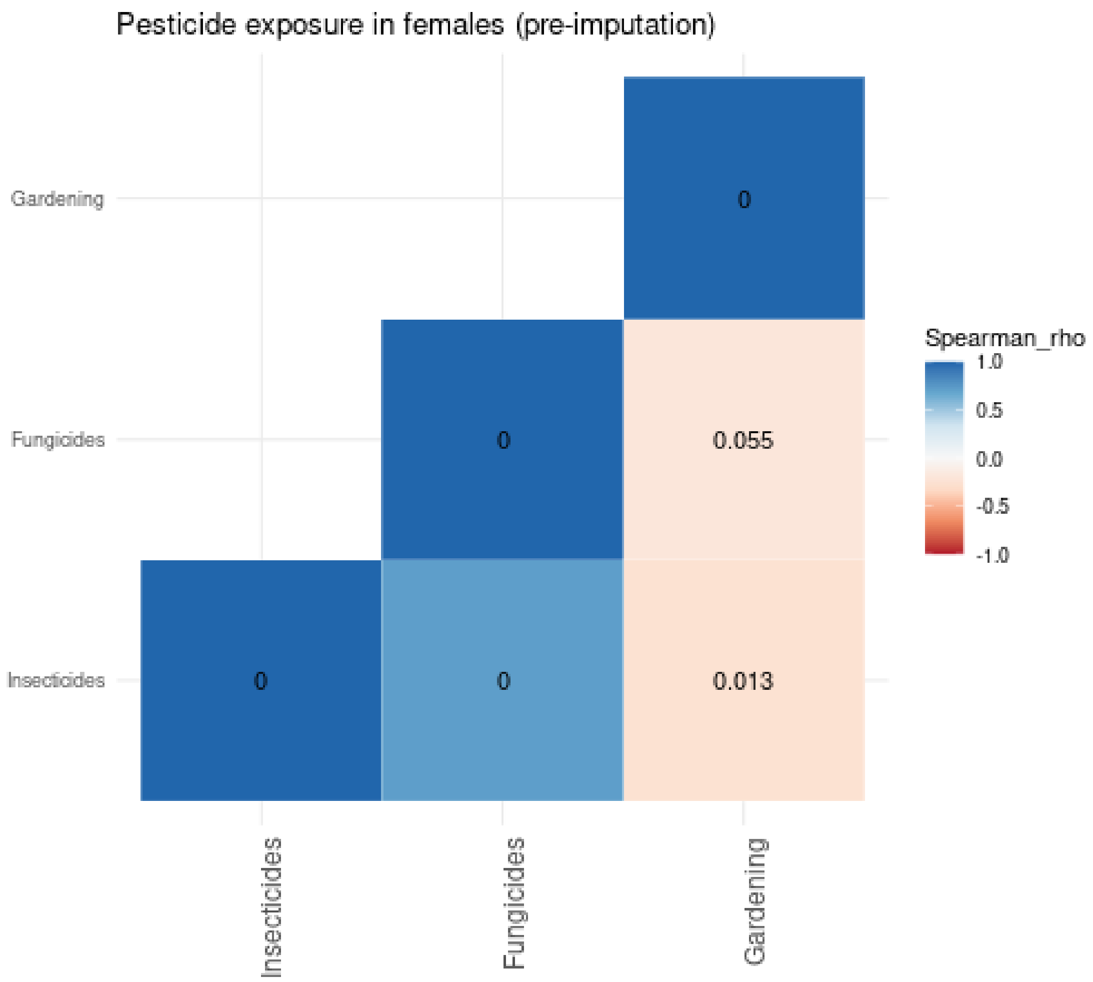

Supplementary Figure 19. Correlation between nonimputed pesticide exposures in females from TERRE. Cell fill color: Spearman's rho. Cell text: Spearman correlation *p*-value ("0": $p < 0.001$). $n = 96$ individuals with complete pesticide data (pre-imputation).

Supplementary Tables

Supplementary Table 1. Full List of Differentially Methylated CMRs in Females from TERRE

CMR coordinates	CMR length (bp)	Number of EPIC probes in CMR	Gene(s)	Gene feature(s)	*p*-value	BH-adjusted *p*-value	Adjusted Δβ
chr15:100913934-100913949	15	2			4.02×10^{-7}	0.003	-0.031
chr13:25506131-25506384	253	4	*TPTE2P1, LOC646405*	Body	1.36×10^{-6}	0.003	-0.071
chr16:1217652-1217858	206	2	*CACNA1H*	Body	1.51×10^{-6}	0.003	0.047
chr11:22454152-22454662	510	6			6.46×10^{-6}	0.007	0.039
chr11:19529541-19530094	553	2	*NAV2, NAV2-AS5*	TSS1500, Body	5.97×10^{-6}	0.007	0.031
chr20:62865953-62866093	140	3	*MYT1*	Body	6.44×10^{-6}	0.007	0.034
chrX:47419673-47419691	18	2	*ARAF*	TSS1500	6.83×10^{-6}	0.007	0.048
chr7:25608634-25608674	40	2			1.03×10^{-5}	0.009	0.046
chr10:3282437-3282651	214	3			1.42×10^{-5}	0.010	0.093
chr18:77280264-77280587	323	3	*NFATC1*	Body	1.89×10^{-5}	0.010	0.064
chr19:57630202-57630662	460	10	*USP29*	TSS1500	2.34×10^{-5}	0.011	0.054
chr19:57630691-57630711	20	2	*USP29*	TSS1500	3.12×10^{-5}	0.013	0.049
chr3:137228231-137228637	406	3			3.47×10^{-5}	0.013	0.046
chr14:99641017-99641152	135	2	*BCL11B*	Body	3.94×10^{-5}	0.014	0.078
chr6:160023581-160024145	564	6			4.11×10^{-5}	0.014	0.107
chr16:80351660-80351734	74	2	*LOC102724084*	Body	4.13×10^{-5}	0.014	0.061
chrX:145509155-145509522	367	2			4.24×10^{-5}	0.014	0.035
chr7:100701511-100701518	7	2	*MUC17*	3'UTR	4.90×10^{-5}	0.015	0.072
chr10:1531243-1531530	287	4	*ADARB2*	Body	5.73×10^{-5}	0.016	0.034
chr5:34494278-34494484	206	2			5.81×10^{-5}	0.016	0.038
chr11:75142012-75142450	438	2	*KLHL35*	TSS1500	7.51×10^{-5}	0.018	0.038
chr12:95226786-95226994	208	2	*MIR492*	TSS1500	9.03×10^{-5}	0.019	-0.082
chr15:33023237-33023587	350	2	*GREM1*	Body	9.60×10^{-5}	0.019	0.067
chr19:18888081-18889004	923	3	*CRTC1*	Body	1.03×10^{-4}	0.019	0.094
chrX:107306969-107307863	894	3	*VSIG1*	Body	1.20×10^{-4}	0.021	0.034
chr15:35086890-35086986	96	4	*ACTC1*	Body	1.37×10^{-4}	0.022	-0.049
chr19:4769206-4769241	35	3	*MIR7-3HG, C19orf30*	TSS1500, Body	1.35×10^{-4}	0.022	0.045
chr7:54955929-54956420	491	6			1.37×10^{-4}	0.022	-0.035
chr5:125694354-125694419	65	2	*GRAMD3*	TSS1500	1.42×10^{-4}	0.022	0.033
chr17:7517217-7517259	42	3	*FXR2, SHBG*	Body, TSS200	1.46×10^{-4}	0.023	0.034
chr12:133179338-133180239	901	4	*LRCOL1*		1.58×10^{-4}	0.024	0.058
chr6:88182107-88182162	55	2	*SLC35A1, CFAP206*	TSS1500	1.61×10^{-4}	0.024	0.054
chr13:88328009-88330615	2606	12	*SLITRK5*	Body	1.68×10^{-4}	0.024	0.068
chr11:62160859-62161094	235	3	*ASRGL1*	3'UTR	1.78×10^{-4}	0.025	0.151
chr6:27569167-27570549	1382	5			2.00×10^{-4}	0.027	-0.079
chr2:213400697-213400870	173	2	*ERBB4*	Body	2.21×10^{-4}	0.028	-0.044
chr20:55904856-55904876	20	2	*SPO11*	5'UTR, 1stExon	2.21×10^{-4}	0.028	0.054
chr8:1156483-1156730	247	2			2.50×10^{-4}	0.029	0.038
chr13:100630170-100630317	147	2			2.54×10^{-4}	0.030	-0.033
chrX:133209900-133210326	426	3			2.58×10^{-4}	0.030	0.047
chr11:8832690-8832743	53	3	*ST5*	TSS1500, 5'UTR	2.59×10^{-4}	0.030	0.042

CMR coordinates	CMR length (bp)	Number of EPIC probes in CMR	Gene(s)	Gene feature(s)	*p*-value	BH-adjusted *p*-value	Adjusted Δβ
chr12:101988604-101988804	200	3	*MYBPC1*	TSS200	2.67×10^{-4}	0.030	-0.045
chr2:10637974-10638073	99	2			2.84×10^{-4}	0.031	0.035
chr17:5290760-5290776	16	2	*NUP88*	Body	2.82×10^{-4}	0.031	-0.044
chr11:27076803-27076820	17	2	*BBOX1*, *BBOX1-AS1*	5'UTR, Body	2.90×10^{-4}	0.031	0.035
chr6:170450803-170452271	1468	4			2.99×10^{-4}	0.031	0.044
chr1:53557455-53558067	612	2	*SLC1A7*	Body	3.60×10^{-4}	0.034	0.031
chr16:89690088-89690803	715	3	*DPEP1*	5'UTR	3.82×10^{-4}	0.035	-0.059
chr6:31126864-31127864	1000	13	*CCHCR1*, *TCF19*	TSS1500, 5'UTR	3.89×10^{-4}	0.035	0.033
chr2:19911427-19911446	19	2			3.81×10^{-4}	0.035	0.035
chr13:112860420-112862113	1693	7			4.16×10^{-4}	0.036	-0.051
chr20:61340827-61340886	59	2	*NTSR1*	1stExon	4.30×10^{-4}	0.037	0.042
chr8:8775232-8775311	79	2			4.38×10^{-4}	0.037	0.036
chr7:155832831-155832993	162	3			4.47×10^{-4}	0.037	0.057
chr5:2334885-2335317	432	3			4.54×10^{-4}	0.037	-0.056
chr22:18049457-18049543	86	2	*SLC25A18*	5'UTR	4.86×10^{-4}	0.038	0.031
chr17:36577690-36577901	211	2			5.23×10^{-4}	0.040	0.040
chr17:7486551-7486875	324	7	*MPDU1*	TSS1500	5.46×10^{-4}	0.041	0.052
chr19:17404960-17404978	18	2	*ABHD8*	Body	5.76×10^{-4}	0.042	0.035
chrX:15584867-15586144	1277	4	*ACE2*	Body	5.93×10^{-4}	0.042	0.033
chr20:49626064-49626862	798	5	*KCNG1*	Body	6.28×10^{-4}	0.044	0.041
chr7:142012611-142012989	378	2			6.59×10^{-4}	0.045	-0.040
chr5:120966065-120966289	224	2			6.80×10^{-4}	0.045	-0.036
chr8:51306508-51306694	186	2	*SNTG1*	5'UTR	6.70×10^{-4}	0.045	0.075
chr18:3624189-3624430	241	2	*DLGAP1*	Body	7.14×10^{-4}	0.046	0.043
chr17:74075092-74075196	104	4	*ZACN*	TSS200	7.46×10^{-4}	0.047	0.051
chr10:3235465-3235570	105	3			7.69×10^{-4}	0.048	0.033
chr5:2008188-2008365	177	4			7.80×10^{-4}	0.048	-0.034
chr2:13147594-13147705	111	2			7.90×10^{-4}	0.048	0.038

n = 33 cases, 67 controls.

BH, Benjamini–Hochberg; bp, base pairs; CMR, comethylated region; PD, Parkinson’s disease.

Supplementary Table 2. Standardized Mean Difference (SMD) in Overall Distance Before and After Propensity Matching Replication Samples

Sample	Sex	Comparison	SMD (distance) before matching	SMD (distance) after matching
DIGPD	F	Case-control	0.567	n/a
DIGPD	M	Case-control	0.834	n/a
PEG1	M	Case-control	0.580	0.241
PEG1	F	Case-control	0.857	0.642
SGPD	F	Case-control	0.567	0.267
SGPD	M	Case-control	0.624	0.236
DIGPD	F	With TERRE	0.785	n/a
DIGPD	M	With TERRE	1.257	n/a
PEG1	M	With TERRE	1.488	0.545
PEG1	F	With TERRE	1.908	0.936
SGPD	M	With TERRE	1.408	0.721
SGPD	F	With TERRE	2.367	1.151

“distance” calculated by “glm” using a probit link function. Two rounds of matching were performed: 1) between cases and controls (on predicted smoking, predicted neutrophil proportion, and age, separately within each sex), followed by 2) between TERRE and each replication sample (on predicted smoking, predicted neutrophil proportion, and age, separately within each sex). “Before” indicates SMD before any matching; “after” indicates SMD after both rounds of matching.

Supplementary Table 3. PD Effect Sizes for Differentially Methylated CMRs Discovered in TERRE Females and Females From PEG1, DIGPD, and SGPD

CMR coordinates	Gene(s)	PD case-control adjusted Δβ				Replicated? (Yes/No)
		TERRE (EPIC)	PEG1 (450K)	DIGPD (EPIC)	SGPD (450K)	
chr13:112860420-112862113		-0.051	n/a	-0.042		Y
chr17:7486551-7486875	*MPDU1*	0.052	0.019	0.058	-0.016	Y
chr18:77280264-77280587	*NFATC1*	0.064	0.032	-0.017	-0.013	Y
chr6:27569167-27570549		-0.079	-0.036	-0.025	0.006	Y
chr5:120966065-120966289		-0.036	n/a	-0.032	n/a	Y
chr1:53557455-53558067	*SLC1A7*	0.031	-0.013	-0.002	0.000	N
chr10:1531243-1531530	*ADARB2*	0.034	0.002	0.001	0.003	N
chr10:3235465-3235570		0.033	0.007	0.010	-0.010	N
chr10:3282437-3282651		0.093	0.025	0.007	-0.003	N
chr11:22454152-22454662		0.039	0.015	0.010	0.002	N
chr11:75142012-75142450	*KLHL35*	0.038	-0.004	-0.021	-0.006	N
chr12:95226786-95226994	*MIR492*	-0.082	-0.013	-0.015	0.002	N
chr12:101988604-101988804	*MYBPC1*	-0.045	n/a	0.024	n/a	N
chr12:133179338-133180239	*LRCOL1*	0.058	-0.019	0.006	0.009	N
chr13:25506131-25506384	*TPTE2P1, LOC646405*	-0.071	-0.014	-0.020	-0.004	N
chr13:88328009-88330615	*SLITRK5*	0.068		-0.005		N
chr13:100630170-100630317		-0.033	0.017	-0.015	-0.003	N
chr14:99641017-99641152	*BCL11B*	0.078	-0.001	-0.039	-0.009	N
chr15:33023237-33023587	*GREM1*	0.067	0.016	-0.019	-0.020	N
chr15:35086890-35086986	*ACTC1*	-0.049	-0.015	-0.015	-0.002	N
chr15:100913934-100913949		-0.031	0.008	-0.002	-0.003	N
chr16:1217652-1217858	*CACNA1H*	0.047	0.002	0.000	0.002	N
chr16:89690088-89690803	*DPEP1*	-0.059	n/a	0.015	n/a	N
chr17:7517217-7517259	*FXR2, SHBG*	0.034	0.009	0.003	0.002	N
chr17:36577690-36577901		0.040	0.002	0.030	0.012	N
chr17:74075092-74075196	*ZACN*	0.051	0.024	0.019	-0.007	N
chr19:4769206-4769241	*MIR7-3HG, C19orf30*	0.045	0.021	0.001	0.000	N
chr19:17404960-17404978	*ABHD8*	0.035	0.004	0.001	-0.006	N
chr19:18888081-18889004	*CRTC1*	0.094	-0.011	-0.018	-0.010	N
chr19:57630202-57630662	*USP29*	0.054	0.025	-0.023	0.003	N
chr19:57630691-57630711	*USP29*	0.049	-0.004	-0.015	0.001	N
chr2:10637974-10638073		0.035	0.000	0.006	0.002	N
chr2:13147594-13147705		0.038	0.001	-0.006	-0.004	N
chr2:213400697-213400870	*ERBB4*	-0.044	-0.007	0.005	-0.001	N
chr20:49626064-49626862	*KCNG1*	0.041	0.000	-0.010	-0.009	N
chr20:55904856-55904876	*SPO11*	0.054	0.017	-0.022	-0.006	N
chr20:61340827-61340886	*NTSR1*	0.042	-0.004	-0.005	0.001	N
chr3:137228231-137228637		0.046	-0.014	0.008	0.004	N
chr5:2008188-2008365		-0.034	0.001	-0.002	-0.001	N
chr5:2334885-2335317		-0.056	-0.012	-0.073	0.008	N
chr5:125694354-125694419	*GRAMD3*	0.033	n/a	-0.019	n/a	N
chr6:31126864-31127864	*TCF19, CCHCR1*	0.033	-0.011	0.002	0.001	N
chr6:160023581-160024145		0.107	-0.003	0.002	0.015	N
chr6:170450803-170452271		0.044	-0.012	-0.030	0.004	N
chr7:25608634-25608674		0.046	0.010	-0.012	-0.001	N
chr7:54955929-54956420		-0.035	0.019	-0.030	0.003	N

CMR coordinates	Gene(s)	PD case-control adjusted Δβ				Replicated? (Yes/No)
		TERRE (EPIC)	PEG1 (450K)	DIGPD (EPIC)	SGPD (450K)	
chr7:142012611-142012989		-0.040	-0.011	0.020	-0.006	N
chr7:155832831-155832993		0.057	-0.035	-0.007	0.001	N
chr8:1156483-1156730		0.038	n/a	0.000	n/a	N
chr11:8832690-8832743	*ST5*	0.042	n/a	0.006	n/a	N
chr11:19529541-19530094	*NAV2, NAV2-AS5*	0.031	n/a	0.011	n/a	N
chr11:27076803-27076820	*BBOX1, BBOX1-AS1*	0.035	n/a	0.009	n/a	N
chr11:62160859-62161094	*ASRGL1*	0.151	n/a	0.021	n/a	N
chr16:80351660-80351734	*LOC102724084*	0.061	n/a	0.017	n/a	N
chr17:5290760-5290776	*NUP88*	-0.044	n/a	-0.001	n/a	N
chr18:3624189-3624430	*DLGAP1*	0.043	n/a	0.030	n/a	N
chr2:19911427-19911446		0.035	n/a	-0.001	n/a	N
chr20:62865953-62866093	*MYT1*	0.034	n/a	-0.010	n/a	N
chr22:18049457-18049543	*SLC25A18*	0.031	n/a	-0.009	n/a	N
chr5:34494278-34494484		0.038	n/a	-0.022	n/a	N
chr6:88182107-88182162	*SLC35A1,CFAP206*	0.054	n/a	-0.018	n/a	N
chr7:100701511-100701518	*MUC17*	0.072	n/a	-0.046	n/a	N
chr8:8775232-8775311		0.036	n/a	-0.025	n/a	N
chr8:51306508-51306694	*SNTG1*	0.075	n/a	0.008	n/a	N
chrX:15584867-15586144	*ACE2*	0.033	n/a	-0.013	n/a	N
chrX:47419673-47419691	*ARAF*	0.048	n/a	-0.029	n/a	N
chrX:107306969-107307863	*VSIG1*	0.034	n/a	-0.019	n/a	N
chrX:133209900-133210326		0.047	n/a	-0.030	n/a	N
chrX:145509155-145509522		0.035	n/a	0.025	n/a	N

CMR: comethylated region. PD: Parkinson’s disease. n/a: not applicable (CMR not present on 450K array). TERRE adjustment covariates: age, smoking, alcohol consumption, head trauma, cell type principal components (PCs) 1-6 (89% of variance), ancestry PCs 1-3, plate, row. PEG1 adjustment covariates: age, ethnicity, predicted smoking (18), cell type PCs 1-3 (87% of variance). DIGPD adjustment covariates: age, predicted smoking, cell type PCs 1-4 (89% of variance), genotype PCs 1-3. SGPD adjustment covariates: age, predicted smoking, cell type PCs 1-3 (83% of variance).

Supplementary Table 4. PD Effect Sizes for Differentially Methylated CMRs Discovered in TERRE Males and Males From PEG1, DIGPD, and SGPD

CMR coordinates	Gene(s)	PD case-control adjusted Δβ				Replicated? (Yes/No)
		TERRE (EPIC)	PEG1 (450K)	DIGPD (EPIC)	SGPD (450K)	
chr19:17433773-17434268	*ANO8,DDA1*	0.047	-0.030	-0.013	-0.002	N
chr6:32294470-32295230	*C6orf10*	0.038	0.014	0.009	-0.003	N

CMR: comethylated region. PD: Parkinson’s disease. n/a: not applicable (CMR not present on 450K array). TERRE adjustment covariates: age, smoking, alcohol consumption, head trauma, cell type principal components (PCs) 1-6 (89% of variance), genotype PCs 1-3, plate, row. PEG1 adjustment covariates: age, ethnicity, predicted smoking (18), cell type PCs 1-3 (87% of variance). DIGPD adjustment covariates: age, predicted smoking, cell type PCs 1-4 (89% of variance), genotype PCs 1-3. SGPD adjustment covariates: age, predicted smoking, cell type PCs 1-3 (83% of variance).

Supplementary Table 5. Sex-Stratified Epigenome-Wide Association Analyses in TERRE with Pesticide Exposure as the Main Effect

Sex	Input	Exposure Variable	Number of DM-CpGs/CMRs $p_{adj} \leq 0.05$, $\lvert\Delta\beta_{adj}\rvert \geq 0.03$
Female	29,363 variable reference CMRs	Gardening	1
		Occupational	0
	433,816 variable CpGs	Gardening	0
		Occupational	0
Male	29,190 variable reference CMRs	Gardening	2
		Occupational	3
	425,581 variable CpGs	Gardening	16
		Occupational	33

CMR median β or CpG β ~ exposure variable + age + smoking + head trauma + cell type PCs 1-6 + genotyping PCs 1-3 + plate + row

Variable CMRs/CpGs: range > 0.05 between the 10th and 90th percentiles of median β values for each CMR, or β values for each CpG

DM: differentially methylated

Supplementary Table 6. Pesticide Exposures with ≥ 10% of Individuals Exposed within Females in TERRE

Pesticide	Average Number of Females Exposed (10 Imputations)	Average Percent of Females Exposed (10 Imputations)
Gardening	28	29%
Insecticides	13	14%
Fungicides	10	10%

Supplementary Table 7. Summary Statistics of the Minimum AIC Model for Each PD-Associated CMR in Females

CMR coordinates	CMR gene(s)	SNP	Nearest Gene to SNP	Exposure	Δβ (E)	Δβ (G)	Δβ (G×E)	AIC	ΔAIC vs. base model	Change in $\lvert\Delta\beta_{adj}\rvert$ (PD) vs. base model	Adjusted *p*-value (F-test)	Repl? (Yes/No)
chr18:3624189-3624430	*DLGAP1*	rs12604364	*DLGAP1-AS2*	n/a	n/a	0.104	n/a	-300.079	-110.212	-0.033	1.41×10^{-15}	Y
chr18:77280264-77280587	*NFATC1*	rs79426764	*DLGAP1-AS2*	Fungicides	1.273	-0.137	-0.631	-164.449	-50.195	-0.018	1.22×10^{-7}	Y
chr6:27569167-27570549	n/a	rs10807026	*RARS2*	Fungicides	0.091	-0.029	-0.142	-261.237	-28.566	0.002	6.57×10^{-6}	Y

CMR coordinates	CMR gene(s)	SNP	Nearest Gene to SNP	Exposure	Δβ (E)	Δβ (G)	Δβ (G×E)	AIC	ΔAIC vs. base model	Change in $\|\Delta\beta_{adj}\|$ (PD) vs. base model	Adjusted *p*-value (F-test)	Repl? (Yes/No)
chr5:2334885-2335317	n/a	rs2628156	*GRAMD2B*	n/a	n/a	-0.037	n/a	-243.621	-14.905	0.004	2.65×10^{-3}	Y
chr13:112860420-112862113	n/a	rs200658161	*TPTE2P1*	n/a	n/a	-0.058	n/a	-199.745	-12.344	-0.010	2.61×10^{-2}	Y
chr5:120966065-120966289	n/a	rs72793312	*GRAMD2B*	n/a	n/a	0.030	n/a	-333.664	-9.688	-0.002	4.30×10^{-2}	Y
chr11:62160859-62161094	*ASRGL1*	rs1043789	*RPS3*	n/a	n/a	-0.318	n/a	-240.611	-221.665	-0.148	6.83×10^{-30}	N
chr17:5290760-5290776	*NUP88*	rs1058398	*NUP88*	n/a	n/a	-0.092	n/a	-338.503	-150.346	0.055	2.23×10^{-24}	N
chr7:142012611-142012989	n/a	rs9648678	*LOC646588*	n/a	n/a	-0.141	n/a	-317.738	-117.306	0.030	2.00×10^{-19}	N
chr1:53557455-53558067	*SLC1A7*	rs7524881	*SLC1A7*	n/a	n/a	-0.091	n/a	-353.394	-126.728	-0.016	1.14×10^{-18}	N
chr8:1156483-1156730	n/a	rs2044924	*LOC286083*	n/a	n/a	0.089	n/a	-330.190	-120.898	-0.057	2.50×10^{-17}	N
chr12:95226786-95226994	n/a	rs7294480	*P2RX2*	n/a	n/a	-0.102	n/a	-421.356	-112.560	0.008	5.87×10^{-17}	N
chr19:17404960-17404978	*ABHD8*	rs35586766	*ANKLE1*	n/a	n/a	0.106	n/a	-356.678	-87.989	-0.011	3.38×10^{-16}	N
chr20:62865953-62866093	*MYT1*	rs2427616	*PCMTD2*	n/a	n/a	0.167	n/a	-298.540	-74.314	0.001	3.68×10^{-12}	N
chr5:125694354-125694419	n/a	rs11738463	*GRAMD2B*	n/a	n/a	-0.049	n/a	-409.434	-52.495	-0.011	1.81×10^{-11}	N
chr8:8775232-8775311	n/a	rs76206107	*LOC286083*	n/a	n/a	-0.069	n/a	-328.379	-64.730	-0.026	1.68×10^{-9}	N
chr15:33023237-33023587	*GREM1*	rs7182522	*GREM1*	n/a	n/a	0.048	n/a	-321.863	-54.679	-0.025	2.76×10^{-8}	N
chr2:213400697-213400870	*ERBB4*	rs13430619	*ODC1-DT*	n/a	n/a	0.076	n/a	-178.712	-36.584	0.025	4.17×10^{-7}	N
chr6:160023581-160024145	n/a	rs4709351	*RARS2*	n/a	n/a	0.115	n/a	-130.047	-24.851	-0.005	4.70×10^{-7}	N
chr16:89690088-89690803	*DPEP1*	rs187720	*CACNA1H*	n/a	n/a	0.061	n/a	-248.184	-50.187	0.012	1.02×10^{-6}	N
chr7:25608634-25608674	n/a	rs17151904	*LOC646588*	n/a	n/a	-0.065	n/a	-265.598	-54.957	-0.031	1.12×10^{-6}	N
chr19:18888081-18889004	*CRTC1*	rs144285351	*ANKLE1*	Gardening	-0.050	0.109	n/a	-183.558	-55.594	-0.028	1.26×10^{-6}	N
chr8:51306508-51306694	*SNTG1*	rs7012871	*LOC286083*	n/a	n/a	-0.054	n/a	-259.962	-35.182	-0.017	6.11×10^{-5}	N
chr16:80351660-80351734	n/a	rs7203873	*CACNA1H*	n/a	n/a	-0.069	n/a	-231.179	-20.806	0.004	3.58×10^{-4}	N
chr11:22454152-22454662	n/a	rs72969039	*RPS3*	Gardening	-0.047	-0.029	0.037	-360.711	-11.980	-0.002	3.95×10^{-4}	N
chr11:19529541-19530094	*NAV2*	rs17594372	*RPS3*	n/a	n/a	-0.027	n/a	-368.991	-24.828	-0.002	6.73×10^{-4}	N
chr6:170450803-170452271	n/a	rs35105151	*RARS2*	n/a	n/a	0.094	n/a	-212.940	-25.931	0.005	7.04×10^{-4}	N

CMR coordinates	CMR gene(s)	SNP	Nearest Gene to SNP	Exposure	Δβ (E)	Δβ (G)	Δβ (G×E)	AIC	ΔAIC vs. base model	Change in $\lvert\Delta\beta_{adj}\rvert$ (PD) vs. base model	Adjusted *p*-value (F-test)	Repl? (Yes/No)
chr19:4769206-4769241	*MIR7-3HG*	rs7257678	*ANKLE1*	Gardening	-0.053	0.012	0.144	-233.055	-32.070	0.010	7.65×10^{-4}	N
chr13:88328009-88330615	*SLITRK5*	rs954384	*TPTE2P1*	n/a	n/a	-0.121	n/a	-118.417	-20.143	0.016	1.52×10^{-3}	N
chr13:25506131-25506384	*TPTE2P1*	rs12864497	*TPTE2P1*	n/a	n/a	-0.031	n/a	-354.430	-17.433	0.007	1.84×10^{-3}	N
chr11:75142012-75142450	n/a	rs534812	*RPS3*	n/a	n/a	0.030	n/a	-292.174	-18.743	0.004	1.89×10^{-3}	N
chr6:88182107-88182162	*SLC35A1,CFAP206*	rs551472764	*RARS2*	n/a	n/a	-0.054	NA	-185.254	-13.156	-0.023	3.13×10^{-3}	N
chr7:100701511-100701518	*MUC17*	rs112404953	*LOC646588*	Gardening	-0.230	-0.094	0.143	-187.439	-18.563	0.029	4.01×10^{-3}	N
chr15:35086890-35086986	*ACTC1*	rs518506	*GREM1*	n/a	n/a	-0.072	n/a	-131.254	-18.790	0.004	4.15×10^{-3}	N
chr2:10637974-10638073	n/a	rs56184404	*ODC1-DT*	Gardening	0.017	0.043	n/a	-407.095	-22.263	0.004	4.60×10^{-3}	N
chr12:133179338-133180239	*LRCOL1*	rs6560888	*P2RX2*	n/a	n/a	0.023	n/a	-406.537	-18.107	-0.001	5.50×10^{-3}	N
chr11:27076803-27076820	*BBOX1*	rs10742165	*RPS3*	Gardening	-0.021	-0.023	0.076	-287.656	-25.125	0.011	9.24×10^{-3}	N
chr10:3282437-3282651	n/a	rs34781940	*LOC105376353*	n/a	n/a	-0.072	n/a	-140.549	-15.186	0.0	1.16×10^{-2}	N
chr5:34494278-34494484	n/a	rs113375591	*GRAMD2B*	Gardening	0.604	0.024	-0.301	-219.616	-13.313	-0.007	1.55×10^{-2}	N
chr19:57630691-57630711	n/a	rs11880915	*ANKLE1*	Fungicides	-0.518	0.033	0.284	-171.674	-19.419	-0.002	1.63×10^{-2}	N
chr12:101988604-101988804	*MYBPC1*	rs12300992	*P2RX2*	n/a	n/a	-0.026	n/a	-373.017	-11.007	0.004	1.70×10^{-2}	N
chr10:1531243-1531530	*ADARB2*	rs5024862	*LOC105376353*	n/a	n/a	-0.031	n/a	-281.055	-13.978	-0.003	1.90×10^{-2}	N
chr7:54955929-54956420	n/a	rs10499751	*LOC646588*	Gardening	0.182	0.0	-0.103	-267.036	-22.031	-0.003	2.03×10^{-2}	N
chr5:2008188-2008365	n/a	rs35973145	*GRAMD2B*	n/a	n/a	0.031	n/a	-230.326	-12.272	0.004	2.47×10^{-2}	N
chr11:8832690-8832743	*ST5*	rs199956041	*RPS3*	n/a	n/a	-0.070	n/a	-282.387	-4.748	0.001	3.24×10^{-2}	N
chr22:18049457-18049543	*SLC25A18*	rs5747205	*CECR2*	n/a	n/a	0.063	n/a	-327.286	-9.881	0.004	3.52×10^{-2}	N
chr16:1217652-1217858	*CACNA1H*	rs11864888	*CACNA1H*	n/a	n/a	-0.077	n/a	-275.213	-5.012	0.004	3.83×10^{-2}	N
chrX:47419673-47419691	n/a	rs3748517	*LINC01560*	Insecticides	0.312	0.044	-0.209	-216.609	-23.891	-0.019	4.48×10^{-2}	N

n/a, An effect not estimated in the minimum AIC model for a given CMR. Repl: replicated CMR.

Supplementary Table 9. Distribution of Demographic Variables by Sex and PD Status in TERRE

Variable	Female Controls<br>*n* = 67	Female Cases<br>*n* = 33	Male Controls<br>*n* = 80	Male Cases<br>*n* = 38
Self-reported race				
White	64 (96%)	33 (100%)	80 (100%)	38 (100%)
North African	3 (4.5%)	0 (0%)	0 (0%)	0 (0%)
Levodopa				
Yes	0 (0%)	23 (70%)	0 (0%)	30 (79%)
No	67 (100%)	10 (30%)	80 (100%)	8 (21%)
Dopamine agonist				
Yes	0 (0%)	26 (79%)	0 (0%)	27 (71%)
No	67 (100%)	7 (21%)	80 (100%)	11 (29%)
Education level				
None to secondary school (age 13)	48 (72%)	24 (73%)	61 (76%)	33 (87%)
High school (age 18)	17 (25%)	8 (24%)	17 (21%)	5 (13%)
University	2 (3.0%)	1 (3.0%)	2 (2.5%)	0 (0%)
Smoking status				
Current	2 (3.0%)	0 (0%)	12 (15%)	1 (2.6%)
Former	3 (4.5%)	2 (6.1%)	31 (39%)	14 (37%)
Never	62 (93%)	31 (94%)	37 (46%)	23 (61%)
Overall pesticide exposure				
Gardening	16 (25%)	12 (38%)	8 (11%)	3 (9.7%)
None	38 (59%)	13 (41%)	3 (4.2%)	4 (13%)
Occupational	10 (16%)	7 (22%)	60 (85%)	24 (77%)
Unknown	3	1	9	7
Head trauma				
No	54 (81%)	29 (88%)	71 (89%)	30 (79%)
Yes	13 (19%)	4 (12%)	9 (11%)	8 (21%)
Alcohol consumption				
Daily	9 (13%)	5 (15%)	53 (66%)	19 (50%)
Never	17 (25%)	9 (27%)	5 (6.2%)	6 (16%)
Occasionally	38 (57%)	16 (48%)	15 (19%)	6 (16%)
Regularly	3 (4.5%)	3 (9.1%)	7 (8.8%)	7 (18%)
Change in alcohol consumption				
Decreased	5 (9.3%)	3 (12%)	21 (28%)	16 (47%)
Increased	1 (1.9%)	1 (3.8%)	3 (3.9%)	0 (0%)

Variable	Female Controls n = 67	Female Cases n = 33	Male Controls n = 80	Male Cases n = 38
No change	48 (89%)	22 (85%)	52 (68%)	18 (53%)
Unknown	13	7	4	4
Marital status				
Divorced, Separated, or Widowed	12 (18%)	5 (15%)	5 (6.2%)	4 (9.1%)
Married	53 (79%)	25 (76%)	70 (88%)	35 (80%)
Single	2 (3.0%)	3 (9.1%)	5 (6.2%)	5 (11%)
Age	67.0 (63.5, 70.4)	66 (62, 70)	70.3 (64.3, 72.9)	69.8 (63.4, 72.1)
Age of onset	n/a	65 (61, 70)	n/a	69 (63, 72)
Disease duration (years)	n/a	0.54 (0.35, 0.79)	n/a	0.63 (0.29, 1.05)
Mini-Mental State Examination	28 (26, 29)	28 (24, 29)	28 (26, 29)	26 (22, 28)
BMI	25.1 (22.6, 28.3)	25.3 (23.2, 28.0)	26.4 (24.3, 30.1)	25.8 (23.6, 28.3)
Unknown	2	1	3	0
Glasses wine per week	8.5 (6.3, 11.0)	7.5 (7.0, 9.5)	14 (8, 27)	14 (7, 30)
Unknown	55	25	22	13
Glasses beer per week	0 (0, 0)	0 (0, 0)	0 (0, 0.5)	0 (0, 0)
Unknown	59	29	41	20
Glasses aperitif per week	1 (1, 1)	1 (0.75, 1.25)	0 (0, 1)	1 (0, 2)
Unknown	58	29	35	18
Predicted neutrophil proportion	0.50 (0.44, 0.56)	0.48 (0.43, 0.58)	0.52 (0.45, 0.57)	0.55 (0.49, 0.59)
Predicted eosinophil proportion	0.000 (0.000, 0.007)	0.000 (0.000, 0.006)	0.000 (0.000, 0.008)	0.000 (0.000, 0.006)
Predicted basophil proportion	0.037 (0.029, 0.044)	0.033 (0.020, 0.040)	0.036 (0.027, 0.044)	0.032 (0.019, 0.041)
Predicted monocyte proportion	0.072 (0.062, 0.085)	0.072 (0.056, 0.081)	0.081 (0.069, 0.093)	0.084 (0.069, 0.094)
Predicted naive B cell proportion	0.034 (0.018, 0.051)	0.035 (0.015, 0.044)	0.029 (0.016, 0.044)	0.025 (0.011, 0.034)
Predicted memory B cell proportion	0.019 (0.007, 0.039)	0.020 (0.014, 0.030)	0.024 (0.010, 0.033)	0.021 (0.010, 0.033)
Predicted $CD4^+$ naive T cell proportion	0.05 (0.02, 0.08)	0.06 (0.03, 0.10)	0.05 (0.02, 0.06)	0.03 (0.02, 0.06)
Predicted $CD4^+$ memory T cell proportion	0.10 (0.07, 0.13)	0.10 (0.08, 0.14)	0.10 (0.07, 0.13)	0.09 (0.06, 0.12)
Predicted regulatory T cell proportion	0.008 (0.000, 0.023)	0.010 (0.001, 0.020)	0.016 (0.003, 0.026)	0.013 (0.000, 0.019)
Predicted $CD8^+$ naive T cell proportion	0.056 (0.045, 0.068)	0.059 (0.042, 0.069)	0.048 (0.036, 0.068)	0.042 (0.027, 0.053)
Predicted $CD8^+$ memory T cell proportion	0.008 (0.000, 0.036)	0.016 (0.000, 0.047)	0.005 (0.000, 0.031)	0.02 (0.00, 0.04)
Predicted NK cell proportion	0.059 (0.050, 0.074)	0.070 (0.063, 0.081)	0.061 (0.054, 0.075)	0.070 (0.054, 0.082)

Categorical variables: *n* (%). Continuous variables: median (IQR). Alcohol consumption was

assessed at physician-conducted interviews according to the following questions: "Do you currently (or have you had in the past used to) consume alcoholic beverages?" (if yes, participants asked to enumerate frequency (occasional consumption at special events/dinners; regular, but not daily consumption; or daily consumption) and average glasses of wine, beer, and aperitifs consumed per week), "Have you always had the same type of consumption of alcoholic beverages?."

Supplementary Table 10. Demographic Comparisons Between TERRE and Replication Samples

	**TERRE (*n* = 218)**	**PEG1 (*n* = 539)**	**DIGPD (*n* = 222)**	**SGPD (*n* = 1751)**
PD cases (*n*, %)	71 (33%)	312 (58%)	110 (50%)	893 (51%)
Age (mean, IQR)	67 (63, 71)	69 (63, 77)	62 (56, 69)	67 (61, 74)
Female (*n*, %)	100 (46%)	232 (43%)	93 (42%)	795 (45%)
Ever pesticide exposure (*n*, %)				
Total	140 (64%)	Unknown	24 (11%)	18%†
Gardening	39 (18%)	39%*	23 (10%)	Unknown
Occupational	101 (46%)	14%*	1 (0.5%)	Unknown
Ever smoker (*n*, %)	65 (30%)	52%±	46 (21%)	53%†

n, The subset from each sample analyzed in this study after quality control on DNAm data and before propensity matching, unless otherwise indicated.

*Based on 1187 participants from the PEG1 study with reported pesticide exposure (19).

±Based on 572 participants from the PEG1 study with DNA methylation data (5).

†Based on 1638 participants from the SGPD study (8).

Supplementary Table 11. Detailed Demographic Comparisons Between TERRE and DIGPD PD Cases (Including Potential Contributors to DNAm Variance Only Available for Cases in DIGPD: Overall Pesticide Exposure, Education Level, Smoking, Head Trauma, and Alcohol Consumption), Stratified by Sex

	**TERRE (*n* = 71 cases)**		**DIGPD (*n* = 110 cases)**	
	**Females (*n* = 33)**	**Males (*n* = 38)**	**Females (*n* = 47)**	**Males (*n* = 63)**
Self-reported race				
White	33 (100%)	38 (100%)	43 (91%)	61 (97%)
North African	0 (0%)	0 (0%)	2 (4%)	1 (1.5%)
Hispanic	0 (0%)	0 (0%)	0 (0%)	0 (0%)
Other (Black, Asian, or Latino)	0 (0%)	0 (0%)	2 (4%)	1 (1.5%)
Levodopa				
Yes	23 (70%)	30 (79%)	19 (40%)	33 (52%)
No	10 (30%)	8 (21%)	28 (60%)	30 (48%)
Dopamine agonist				
Yes	26 (79%)	27 (71%)	27 (57%)	38 (60%)
No	7 (21%)	11 (29%)	20 (43%)	25 (40%)
Education level				
None to secondary school (age 13)	24 (73%)	33 (87%)	11 (23%)	8 (13%)
High school (age 18)	8 (24%)	5 (13%)	19 (40%)	23 (37%)
University	1 (3%)	0 (0%)	17 (36%)	30 (48%)
Unknown	0 (0%)	0 (0%)	0 (0%)	2 (3%)
Smoking status				
Current	0 (0%)	1 (3%)	3 (6%)	7 (11%)
Former	2 (6%)	14 (37%)	9 (19%)	27 (43%)
Never	31 (94%)	23 (61%)	33 (70%)	27 (43%)
Unknown	0 (0%)	0 (0%)	2 (4%)	2 (3%)
Overall pesticide exposure				
None	13 (39%)	4 (11%)	33 (70%)	40 (63%)
Gardening	12 (36%)	3 (8%)	6 (13%)	17 (27%)
Occupational	7 (21%)	24 (63%)	0 (0%)	1 (2%)
Unknown	1 (3%)	7 (18%)	8 (17%)	5 (8%)
Head trauma				

	TERRE (*n* = 71 cases)		DIGPD (*n* = 110 cases)	
	Females (*n* = 33)	Males (*n* = 38)	Females (*n* = 47)	Males (*n* = 63)
Yes	4 (12%)	8 (21%)	5 (11%)	13 (21%)
No	29 (88%)	30 (79%)	40 (85%)	48 (76%)
Unknown	0 (0%)	0 (0%)	2 (4%)	2 (3%)
Alcohol consumption				
Never	9 (27%)	6 (16%)	10 (21%)	3 (5%)
Occasionally	16 (48%)	6 (16%)	22 (47%)	22 (35%)
Regularly	8 (24%)	26 (68%)	12 (26%)	36 (57%)
Unknown	0 (0%)	0 (0%)	3 (6%)	2 (3%)
Disease duration (years)	0.54 (0.35, 0.79)	0.63 (0.29, 1.05)	1.16 (0.71, 1.58)	1.17 (0.69, 1.65)
Mini-Mental State Examination	28 (24, 29)	26 (22, 28)	28 (27, 30)	28 (28, 30)

Alcohol consumption was assessed at physician-conducted interviews according to the question: "Do you currently (or have you had in the past used to) consume alcoholic beverages?" (if yes, participants asked to enumerate frequency (occasional consumption at special events/dinners; regular, but not daily consumption; or daily consumption).

Supplementary Table 12. Summary of Subject Filtering in All Data Sets, Based on DNAm QC Checks

Sample	TERRE	DIGPD		PEG1	SGPD
		Total	Visit 1		
**Total *n* before filtering**	**265**	**523**	**342**	**567**	**1889**
ewastools control metrics (failing 2 or more) (20)	26	19	4	1	10
minfi M/U channel intensity (21)	0	0	0	1 (0 additional)	9 (8 additional)
Sex mismatches (Xchr PCA, Ychr missingness, minfi, conumee) (21,22)	3 (2 additional)	7 (6 additional)	5 (4 additional)	6 (5 additional)	3 (1 additional)
SNP control probes clustering	2	0	0	1	2
ewastools SNP outlier calling (contamination check) (20)	3 (1 additional)	14 (13 additional)	9 (8 additional)	1	5 (4 additional)
minfi detection p-value (> 0.01 at > 5% of probes) (21)	1 (0 additional)	1	0	7 (5 additional)	15 (3 additional)
Bead count < 3 at > 1% of probes	0	2	2	0	1
Average intensity < mean intensity - 2 × standard deviation	1 (0 additional)	22	17	8 (7 additional)	43
Lumi outlier detection (23)	3 (2 additional)	33 (27 additional)	30 (25 additional)	16 (8 additional)	82 (59 additional)
wateRmelon outlier detection (24)	1 (0 additional)	21 (0 additional)	21 (0 additional)	6 (0 additional)	16 (3 additional)

Intersample correlation ($r < 0.95$)	0	28<br>(0 additional)	28<br>(0 additional)	4<br>(0 additional)	6<br>(0 additional)
PCA clustering	0	29<br>(0 additional)	29<br>(0 additional)	5<br>(0 additional)	0
Horvath DNAm age < 20 years (25)	0	2<br>(0 additional)	2<br>(0 additional)	5<br>(0 additional)	3
β distribution	4<br>(1 additional)	13<br>(8 additional)	9<br>(4 additional)	6<br>(0 additional)	1
**Total subjects removed**	**34**	**98**	**64**	**28**	**138**
**Total *n* after filtering**	**231**	**425**	**278**	**539**	**1751**

For each row, "additional" indicates the number of subjects not already flagged in the checks listed above. All time points of the longitudinal DIGPD study were processed together ("Total" column); the numbers of subjects removed from the first time point of DIGPD presented in this manuscript are indicated in the "Visit 1" column.

Supplementary Table 13. Summary of DNAm Array Probe Filtering in All Data Sets

Sample	TERRE	DIGPD	PEG1	SGPD
**Total probes before filtering**	**865,859**	**865,859**	**485,512**	**485,512**
> 5% missing values<br>(bead count < 3 or detection $p > 0.01$)	9,506	9,615	1,462	812
Cross-hybridizing (26)	43,177<br>(42,766 additional)	43,177<br>(42,749 additional)	41,937<br>(41,808 additional)	41,937<br>(41,865 additional)
Polymorphic (26)	11,681<br>(9810 additional)	11,681<br>(9761 additional)	20,869<br>(17,979 additional)	20,869<br>(18,136 additional)
**Total probes removed**	**62,082**	**62,125**	**61,249**	**60,813**
**Total probes after filtering**	**803,777**	**803,734**	**424,263**	**424,699**

For each row, "additional" indicates the number of probes not already flagged in the checks listed above.

Supplementary Table 14. Model Fit Assessment with Akaike Information Criterion (AIC)

Model	Females		Males	
	**AIC**	**Delta AIC (vs. base)**	**AIC**	**Delta AIC (vs. base)**
Base	−430.67	n/a	−509.57	n/a
Base + smoking	−427.90	2.77	−507.74	1.83
Base + smoking + alcohol consumption	−428.12	2.55	−507.22	2.35
Base + smoking + alcohol consumption + head trauma	−427.53	3.14	−507.40	2.17

AIC values and delta AIC compared to the base model for each sex are shown.

Base model: Median CMR β ~ PD + age + cell type PCs 1–6 + genotype PCs 1–3 + plate + row

Supplementary Table 15. Variance Inflation Factors for each Covariate in Final Models

Covariate	Variance inflation factor	
	Females	Males
PD	1.30	1.41
Age	1.66	1.48
Alcohol consumption	1.54	1.55
Smoking	1.18	1.25
Head trauma	1.53	1.62
Cell type PC 1	1.75	1.54
Cell type PC 2	2.29	1.60
Cell type PC 3	3.48	1.61
Cell type PC 4	2.38	1.71
Cell type PC 5	1.81	1.77
Cell type PC 6	1.99	1.52
Genotype PC 1	1.70	1.55
Genotype PC 2	1.70	1.40
Genotype PC 3	1.65	1.42
Plate	1.50	1.47
Row	1.38	1.40

References

1. Elbaz A, Clavel J, Rathouz PJ, Moisan F, Galanaud JP, Delemotte B, et al. Professional exposure to pesticides and Parkinson disease. Ann Neurol. 2009;66(4):494–504.
2. Bower JH, Maraganore DM, McDonnell SK, Rocca WA. Incidence and distribution of parkinsonism in Olmsted County, Minnesota, 1976-1990. Neurology. 1999 Apr 12;52(6):1214–20.
3. Corvol JC, Artaud F, Cormier-Dequaire F, Rascol O, Durif F, Derkinderen P, et al. Longitudinal analysis of impulse control disorders in Parkinson disease. Neurology. 2018 Jul 17;91(3):e189–201.
4. Gibb WR, Lees AJ. The relevance of the Lewy body to the pathogenesis of idiopathic Parkinson's disease. J Neurol Neurosurg Psychiatry. 1988 Jun;51(6):745–52.
5. Chuang YH, Paul KC, Bronstein JM, Bordelon Y, Horvath S, Ritz B. Parkinson's disease is associated with DNA methylation levels in human blood and saliva. Genome Med. 2017 Dec;9(1):76.
6. Gelb DJ, Oliver E, Gilman S. Diagnostic criteria for Parkinson disease. Arch Neurol. 1999 Jan;56(1):33–9.
7. Costello S, Cockburn M, Bronstein J, Zhang X, Ritz B. Parkinson's disease and residential exposure to maneb and paraquat from agricultural applications in the central valley of California. Am J Epidemiol. 2009 Apr 15;169(8):919–26.
8. Vallerga CL, Zhang F, Fowdar J, McRae AF, Qi T, Nabais MF, et al. Analysis of DNA methylation associates the cystine-glutamate antiporter SLC7A11 with risk of Parkinson's disease. Nat Commun. 2020 Dec 1;11(1):1238.
9. Calne DB, Snow BJ, Lee C. Criteria for diagnosing Parkinson's disease. Ann Neurol. 1992;32 Suppl:S125-127.
10. Lam M, Awasthi S, Watson HJ, Goldstein J, Panagiotaropoulou G, Trubetskoy V, et al. RICOPILI: Rapid Imputation for COnsortias PIpeLIne. Bioinformatics. 2020 Feb 1;36(3):930–3.
11. Chang CC, Chow CC, Tellier LCAM, Vattikuti S, Purcell SM, Lee JJ. Second-generation PLINK: rising to the challenge of larger and richer datasets. GigaScience. 2015 Feb 25;4:7.
12. Galinsky KJ, Bhatia G, Loh PR, Georgiev S, Mukherjee S, Patterson NJ, et al. Fast Principal-Component Analysis Reveals Convergent Evolution of ADH1B in Europe and East Asia. Am J Hum Genet. 2016 Mar 3;98(3):456–72.
13. Danecek P, Bonfield JK, Liddle J, Marshall J, Ohan V, Pollard MO, et al. Twelve years of SAMtools and BCFtools. GigaScience. 2021 Feb 16;10(2):giab008.
14. Ho D, Imai K, King G, Stuart EA. MatchIt: nonparametric preprocessing for parametric causal inference. J Stat Softw. 2011 Jun 14;42:1–28.
15. Hansen BB, Klopfer SO. Optimal Full Matching and Related Designs via Network Flows. J Comput Graph Stat. 2006 Sep 1;15(3):609–27.
16. Modern Applied Statistics with S, 4th ed [Internet]. [cited 2023 Mar 24]. Available from: https://www.stats.ox.ac.uk/pub/MASS4/
17. Koestler DC, Jones MJ, Usset J, Christensen BC, Butler RA, Kobor MS, et al. Improving cell mixture deconvolution by identifying optimal DNA methylation libraries (IDOL). BMC Bioinformatics. 2016 Mar 8;17:120.

18. Bollepalli S, Korhonen T, Kaprio J, Anders S, Ollikainen M. EpiSmokEr: a robust classifier to determine smoking status from DNA methylation data. Epigenomics. 2019 Oct;11(13):1469–86.
19. Narayan S, Liew Z, Bronstein JM, Ritz B. Occupational pesticide use and Parkinson's disease in the Parkinson Environment Gene (PEG) study. Environ Int. 2017 Oct;107:266–73.
20. Heiss JA, Just AC. Identifying mislabeled and contaminated DNA methylation microarray data: an extended quality control toolset with examples from GEO. Clin Epigenetics. 2018;10:73.
21. Aryee MJ, Jaffe AE, Corrada-Bravo H, Ladd-Acosta C, Feinberg AP, Hansen KD, et al. Minfi: a flexible and comprehensive Bioconductor package for the analysis of Infinium DNA methylation microarrays. Bioinformatics. 2014 May 15;30(10):1363–9.
22. Hovestadt V, Zapatka M. conumee: Enhanced copy-number variation analysis using Illumina DNA methylation arrays [Internet]. Bioconductor version: Release (3.16); 2023 [cited 2023 Jan 25]. Available from: https://bioconductor.org/packages/conumee/
23. Du P, Kibbe WA, Lin SM. lumi: a pipeline for processing Illumina microarray. Bioinformatics. 2008 Jul;24(13):1547–8.
24. Pidsley R, Wong CCY, Volta M, Lunnon K, Mill J, Schalkwyk LC. A data-driven approach to preprocessing Illumina 450K methylation array data. BMC Genomics. 2013 May 1;14(1):293.
25. Horvath S. DNA methylation age of human tissues and cell types. Genome Biol. 2013 Oct 21;14(10):R115.
26. Pidsley R, Zotenko E, Peters TJ, Lawrence MG, Risbridger GP, Molloy P, et al. Critical evaluation of the Illumina MethylationEPIC BeadChip microarray for whole-genome DNA methylation profiling. Genome Biol. 2016 Oct 7;17(1):208.
