# Supplementary material for: Genetic variation and pesticide exposure influence blood DNA methylation signatures in females with early-stage Parkinson’s disease
Source: NPJ Parkinsons Dis. 2024 May 7;10:98. doi: 10.1038/s41531-024-00704-3 (PMC11076573; doi:10.1038/s41531-024-00704-3)
Supplement: Supplementary file 3 — Reporting Summary [file 41531_2024_704_MOESM3_ESM.pdf]

Corresponding author(s): Dr. Michael Kobor

Last updated by author(s): Feb 29, 2024

Reporting Summary

Nature Portfolio wishes to improve the reproducibility of the work that we publish. This form provides structure for consistency and transparency in reporting. For further information on Nature Portfolio policies, see our Editorial Policies and the Editorial Policy Checklist.

Statistics

For all statistical analyses, confirm that the following items are present in the figure legend, table legend, main text, or Methods section.

n/a	Confirmed	
☐	☒	The exact sample size (*n*) for each experimental group/condition, given as a discrete number and unit of measurement
☐	☒	A statement on whether measurements were taken from distinct samples or whether the same sample was measured repeatedly
☐	☒	The statistical test(s) used AND whether they are one- or two-sided<br>*Only common tests should be described solely by name; describe more complex techniques in the Methods section.*
☐	☒	A description of all covariates tested
☐	☒	A description of any assumptions or corrections, such as tests of normality and adjustment for multiple comparisons
☐	☒	A full description of the statistical parameters including central tendency (e.g. means) or other basic estimates (e.g. regression coefficient) AND variation (e.g. standard deviation) or associated estimates of uncertainty (e.g. confidence intervals)
☐	☒	For null hypothesis testing, the test statistic (e.g. *F*, *t*, *r*) with confidence intervals, effect sizes, degrees of freedom and *P* value noted<br>*Give P values as exact values whenever suitable.*
☒	☐	For Bayesian analysis, information on the choice of priors and Markov chain Monte Carlo settings
☒	☐	For hierarchical and complex designs, identification of the appropriate level for tests and full reporting of outcomes
☐	☒	Estimates of effect sizes (e.g. Cohen's *d*, Pearson's *r*), indicating how they were calculated

Our web collection on statistics for biologists contains articles on many of the points above.

Software and code

Policy information about availability of computer code

Data collection	GEO data was downloaded using the GEOquery R package, v2.7.0. Code is available at github.com/samschaf/TERRE_GxE.
Data analysis	Data analysis was conducted in R v3.6.2, with relevant packages and functions described in the Methods section of the manuscript. Code is available at github.com/samschaf/TERRE_GxE.

For manuscripts utilizing custom algorithms or software that are central to the research but not yet described in published literature, software must be made available to editors and reviewers. We strongly encourage code deposition in a community repository (e.g. GitHub). See the Nature Portfolio guidelines for submitting code & software for further information.

Data

Policy information about availability of data

All manuscripts must include a data availability statement. This statement should provide the following information, where applicable:

- Accession codes, unique identifiers, or web links for publicly available datasets
- A description of any restrictions on data availability
- For clinical datasets or third party data, please ensure that the statement adheres to our policy

The TERRE and DIGPD DNAm and genotyping data analyzed in the present study are subject to access restrictions via the European Union General Data Protection Regulation (GDPR) and to maintain participant privacy. Requests for access can be directed to alexis.elbaz@inserm.fr, including the proposed purpose for data use,

and are subject to governance constraints and privacy re-strictions. The PEG1 and SGPD DNAm data analyzed in this study are available on GEO (GSE111629, GSE145361).

Research involving human participants, their data, or biological material

Policy information about studies with human participants or human data. See also policy information about sex, gender (identity/presentation), and sexual orientation and race, ethnicity and racism.

Reporting on sex and gender	Participant sex was self-reported, and sex was confirmed using DNA methylation patterns on the sex chromosomes and with DNA methylation-based sex predictors. There was no available information on gender, and as such, any individuals with discordance between predicted and reported sex were excluded from the analysis. We conducted sex-stratified data analysis and refer to sex throughout the manuscript, with some mention of potential gender effects in the Discussion.
Reporting on race, ethnicity, or other socially relevant groupings	Participant race/ethnicity was self-reported. This can associate with DNA methylation and genotype patterns. In the TERRE sample, the few non-European individuals clustered with European individuals on genotyping PCs, and there were not enough non-European individuals to adjust for ethnicity as a covariate in models for the TERRE or DIGPD samples (<6%). We adjusted for genotype PCs in our models, which are a continuous measure that can be correlated with ethnicity, and were more feasible to correct for in all samples. In the replication analysis with PEG1, CMR effect sizes were adjusted for ethnicity. The SGPD sample included only European-descent individuals.
Population characteristics	Population demographics for each sample used in this study are presented in Table S10. In brief, the TERRE sample was 46% female, mean age 67, 99% self-reported White; the DIGPD sample was 42% female, mean age 62, 94% self-reported White; the PEG1 sample was 43% female, mean age 69, 89% self-reported White; and the SGPD sample was 45% female, mean age 67, 100% self-reported White.
Recruitment	Participant recruitment is described in detail in the manuscript and in Additional File 1, p.3. In brief, TERRE participants were recruited through the Mutualité Sociale Agricole healthcare system; DIGPD participants were recruited at 8 French hospitals; PEG1 participants were recruited through three counties in California; and SGPD participants were recruited from hospitals and communities based in Australia and New Zealand.
Ethics oversight	The research protocol of the TERRE study was approved by the ethics committee of Hôpital du Kremlin-Bicêtre, and all subjects provided written informed consent. The research protocol of the DIGPD study was approved by the ethics committee of University of Paris VI, and all subjects provided written informed consent.

Note that full information on the approval of the study protocol must also be provided in the manuscript.

Field-specific reporting

Please select the one below that is the best fit for your research. If you are not sure, read the appropriate sections before making your selection.

☒ Life sciences ☐ Behavioural & social sciences ☐ Ecological, evolutionary & environmental sciences

For a reference copy of the document with all sections, see nature.com/documents/nr-reporting-summary-flat.pdf

Life sciences study design

All studies must disclose on these points even when the disclosure is negative.

Sample size	Samples were selected from TERRE participants with early disease duration (<=1.5 years) and of male or female sex. Power calculations and null simulations were used to determine whether sample sizes were sufficient for detection of PD-associated DNA methylation patterns using the approaches described in this manuscript.
Data exclusions	All individuals from each sample (TERRE, DIGPD, PEG1, SGPD) passing DNA methylation quality control checks were included in analyses. Individuals from DIGPD with disease duration > 2 years at baseline or with familial PD mutations were excluded, as the scope of this study was focused on early-stage PD, and as familial PD has a unique molecular profile compared with sporadic PD.
Replication	We attempted to replicate PD-associated DNA methylation patterns detected in TERRE by comparing the case-control DNA methylation differences at the same regions in individuals from the DIGPD, PEG1, and SGPD samples, within each sex. A small number of CMRs in females replicated in at least one other sample. Despite adjustment for confounders to the best of our ability (through propensity matching and covariate adjustment), we believe differences in sample demographics, lifestyle/exposures, and/or blood cell type composition may have contributed to failure to replicate some of regional DNA methylation changes observed in TERRE. However, we note our replication rate was comparable to or greater than previous epigenome-wide association studies of PD and other phenotypes.
Randomization	TERRE and DIGPD DNA samples were randomized on EPIC BeadChip arrays by disease status. During data analysis, samples were sex-stratified, and confounders (genetic ancestry, blood cell type composition, smoking, age, head trauma, alcohol consumption, batch effects) were accounted for via propensity-matched weights, covariate adjustment, and by using a robust linear regression approach.
Blinding	Blinding to disease status was not possible during biosample collection. However, technicians running samples on the DNA methylation and genotyping arrays were blinded, as samples were pre-randomized by disease status and tubes were labelled according to de-identified study IDs. Disease status was not taken into consideration during inclusion/exclusion based on the DNA methylation sample quality control metrics.

Reporting for specific materials, systems and methods

We require information from authors about some types of materials, experimental systems and methods used in many studies. Here, indicate whether each material, system or method listed is relevant to your study. If you are not sure if a list item applies to your research, read the appropriate section before selecting a response.

Materials & experimental systems

n/a	Involved in the study
☒	☐ Antibodies
☒	☐ Eukaryotic cell lines
☒	☐ Palaeontology and archaeology
☒	☐ Animals and other organisms
☒	☐ Clinical data
☒	☐ Dual use research of concern
☒	☐ Plants

Methods

n/a	Involved in the study
☒	☐ ChIP-seq
☒	☐ Flow cytometry
☒	☐ MRI-based neuroimaging

Plants

Seed stocks

Report on the source of all seed stocks or other plant material used. If applicable, state the seed stock centre and catalogue number. If plant specimens were collected from the field, describe the collection location, date and sampling procedures.

Novel plant genotypes

Describe the methods by which all novel plant genotypes were produced. This includes those generated by transgenic approaches, gene editing, chemical/radiation-based mutagenesis and hybridization. For transgenic lines, describe the transformation method, the number of independent lines analyzed and the generation upon which experiments were performed. For gene-edited lines, describe the editor used, the endogenous sequence targeted for editing, the targeting guide RNA sequence (if applicable) and how the editor was applied.

Authentication

Describe any authentication procedures for each seed stock used or novel genotype generated. Describe any experiments used to assess the effect of a mutation and, where applicable, how potential secondary effects (e.g. second site T-DNA insertions, mosiacism, off-target gene editing) were examined.
